# One-Pot Cyclization and Cleavage of Peptides with *N*-Terminal Cysteine via the *N,S*-Acyl Shift of the *N*-2-[Thioethyl]glycine Residue

**DOI:** 10.1021/acs.joc.1c01045

**Published:** 2021-08-06

**Authors:** Magdalena Wierzbicka, Mateusz Waliczek, Anna Dziadecka, Piotr Stefanowicz

**Affiliations:** Faculty of Chemistry, University of Wroclaw, F. Joliot-Curie 14, 50-383 Wroclaw, Poland

## Abstract

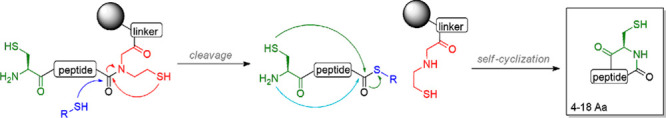

We developed a one-pot method for peptide cleavage from a solid support *via* the *N,S*-acyl shift of *N*-2-[thioethyl]glycine and transthioesterification using external thiols to produce cyclic peptides through native chemical self-ligation with the *N*-terminal cysteine. The feasibility of this methodology is validated by the syntheses of model short peptides, including a tetrapeptide, the bicyclic sunflower trypsin inhibitor SFTI-1, and rhesus Θ-defensin RTD-1. Synthesis of the whole peptide precursor can be fully automated and proceeds without epimerization or dimerization.

Cyclic peptides are an important class of biologically active natural products. Their conformational stability enables selective binding to their receptors. The biological significance makes them interesting targets for drug development, which in turn necessitates the syntheses of their multiple variants. The cyclization of linear precursors is the yield-determining step and can be reached in several ways.^1−3^ One of the most extensively used methodologies is native chemical ligation (NCL)^4^ developed by Kent et al., which is based on the chemoselective reaction between peptide segments containing *N*-terminal cysteine and *C*-terminal thioester groups.^5^ Peptide thioesters with a *N*-terminal cysteine undergo intramolecular NCL to yield homodetic cyclic peptides. However, the synthesis of peptide thioesters by Fmoc-based solid-phase peptide synthesis is challenging due to the instability of thioesters under the piperidine-mediated Fmoc deprotection step and requires the so-called safety-catch linkers, for example, sulfonamide,^6−8^ trithioortho esters,^9^ or aryl and hydrazine linkers.^10,11^ The peptide thioesters also can be formed by *N,S*-acyl shift and transthioesterification with external thiols. Selenocysteine and *N*- and α-alkyl cysteines are widely known as thioester precursors and surrogates.^12,13^ Additionally, bis(2-sulfanylethyl)amido (SEA),^14,15^*N*-sulfanylethylanilides (SEAlides),^16^ and thioethylalkylamido (TEA)^17^ surrogates are commonly used.^18,19^ They were applied in the synthesis of cyclic peptides, including SFTI-1,^20^ cyclopsychotride,^21^ conotoxin MVII,^13^ kalata 1,^11^ and McoTI-II.^22^ One of the recent additions to the above-mentioned class of thioesterification devices is the *N*-(2-hydroxybenzyl)cysteine,^23^ which is inspired by the natural intein splicing process. The cyclization in native conditions is the method of choice for the preparation of bicyclic and cysteine-rich peptides due to the spontaneous oxidative formation of disulfide bridges. Currently, some on-resin peptide NCL cyclization methods are being developed. This approach reduces the number of synthetic steps and simplifies peptide purification from excess reagents, thus combining the advantages of both NCL and solid-phase peptide synthesis (SPPS).^8,11^ Recently, Dawson’s *o*-amino(methyl)aniline (MeDBz) linker was applied for the on-resin one-pot preparation of Kalata B1 and McoTI-II,^24^ SFTI-1,^25,26^ and cyclotetrapeptides.^26^ Another example of this approach is *N*-ethylcysteine-mediated ligation.^27^ However, this method is limited by the less-efficient *N*-acylation of *N*-ethylcysteine, which necessitates the coupling of a preformed in-solution dipeptide to the solid support. In this article, we employed the novel building block *N*-2-[thioethyl]glycine that can be synthesized from commercially available substrates, loaded on the solid support by a submonomeric approach, and is susceptible to amide–thioester rearrangement, allowing intramolecular native chemical ligation with the *N*-terminal cysteine (Scheme 1). The simplicity of the synthesis of the peptide precursor, which can be automated, and the one-pot cyclization were the main advantages of the proposed methods over described protocols.

**Scheme 1:**
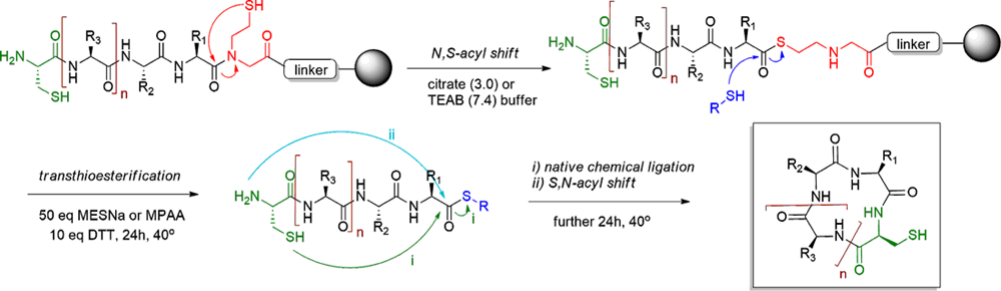
Mechanism of the One-Pot Cyclization

Our methodology is validated by the synthesis of diverse cyclic peptides containing cysteine residues, (Figure 1). TentaGel-NH_2_ resin, which is compatible with both organic and aqueous reaction conditions, was chosen. For the final deprotection that maintained the peptide precursor on a solid support, we used an orthogonal AAM linker that could be cleaved by BrCN. For longer polypeptides, we replaced alanine residues with either β-alanines or polyethylene glycol to increase the distance between the solid support and the peptide chain. They were tested on the model sequence CAKPGG-*N*-2-[thioethyl]glycine, and we found that the type of linker had no impact on the product formation (SI 4). For the synthesis of our device, we used commercially available 2-(thio)ethamine (cysteamine) hydrochloride and performed the thiol protection using both trityl (trt) and 4-methoxytrityl (mmt) chlorides to synthesize (trt)cysteamine **6** (Scheme 2a) and (mmt)cysteamine (**6a**, SI 1.2). Their syntheses were previously reported by O’Neil^28^ and Riddoch,^29^ but herein we adapted the procedure from Barlos,^30^ proceeding in mild DMF conditions. To insert the *S*-protected cysteamine, the resin that was preloaded with the AAM sequence was coupled with chloroacetic acid (Scheme 2a). Then, the *S*-protected cysteamine was introduced through S_N_2 substitution. The target peptide precursors (Scheme 2b) were then assembled on the solid support either manually via a sonication-assisted Fmoc-SPPS protocol^31^ or automatically. The solid-phase synthesis of peptide precursors was monitored by BrCN cleavage, followed by LC-UV-MS. We compared several conditions for the manual, partially automated, and fully automated (with or without microwave heating) syntheses of peptide precursors with different durations for (trt)cysteamine incorporation (Figure S6). We observed that the reaction of (trt)cysteamine with the solid support can be completed in 1–2 h instead of overnight when using microwave-assisted heating at 75 °C. The microwave heating should not be applied for the coupling of chloroacetic acid.

**Figure 1:**
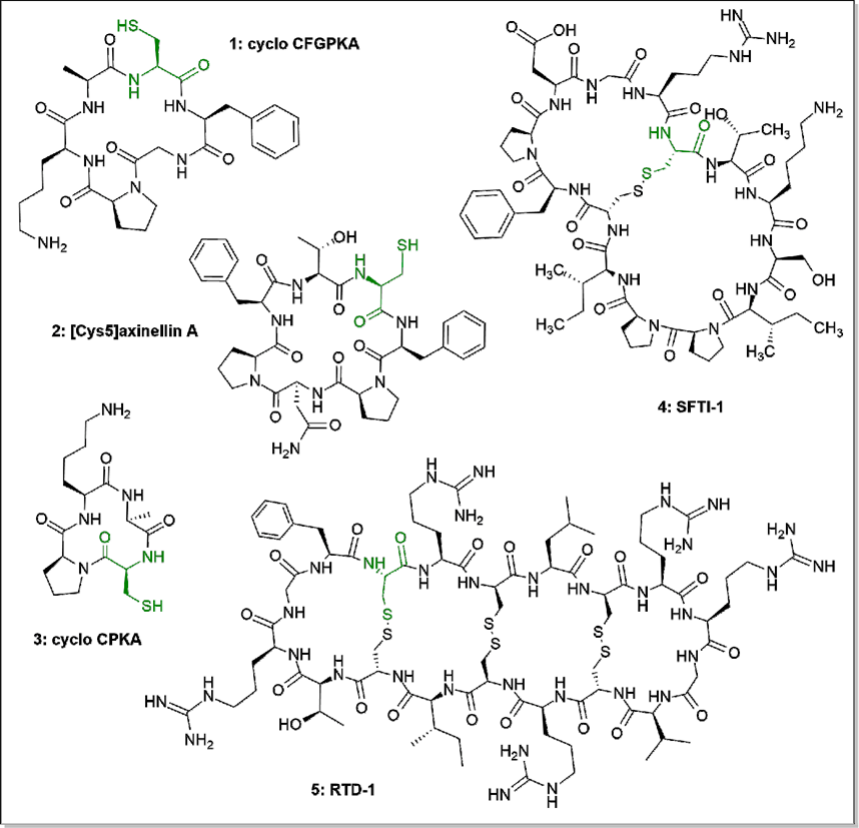
Structures of cyclic peptides **1**–**5** obtained by the presented method. The *N*-terminal cysteine moiety is marked in green.

**Scheme 2:**
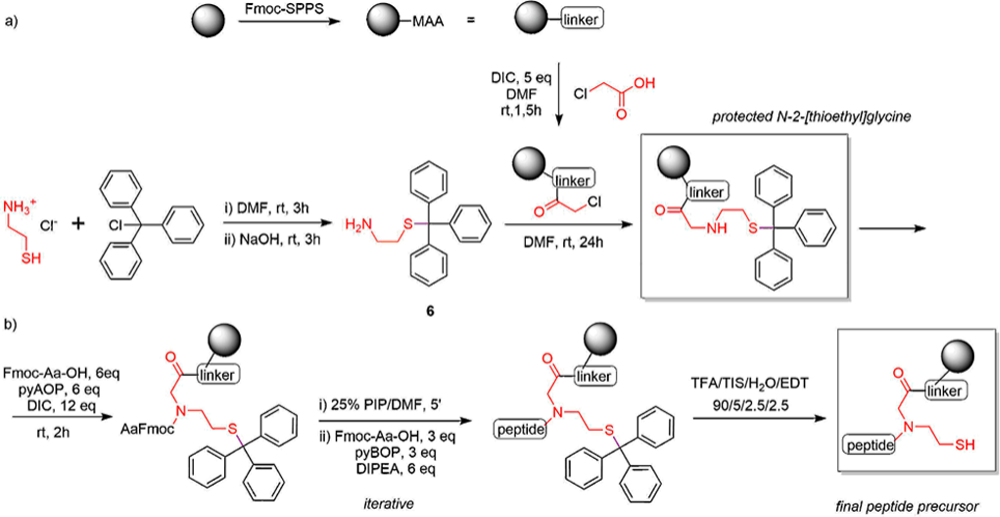
Synthesis of *S*-Trityl Cysteamine **6**, (a) Its Incorporation into the Solid Support, and (b) Further Peptide Precursor Synthesis and Deprotection

After the Fmoc-SPPS of the peptide precursors, we tested multiple conditions to study the rates of the *N,S*-acyl shift, transthioesterification, and intramolecular ligation in neutral and acidic conditions in the presence of various thiol additives. They are listed in Table 2 in the Peptide Cyclization Procedures section and are described in detail. The peptidyl resin was incubated in the buffer containing 50 equiv of an external thiol in the presence of a reducing agent at 40–50 °C for 48 h to promote the *N,S*-acyl shift and transthioesterification, leading to the release of the peptide-active thioester from a solid support. The final step involves the intramolecular NCL reaction between the *N*-terminal cysteine residue and the *C*-terminal thioester, which affords the desired head-to-tail cyclic product. Cyclization was initially performed with MESNa in citrate buffer, which was then followed by a second transthioesterification with MPAA in phosphate buffer to enhance the rate of NCL (procedure A). These reaction conditions were adopted from Taichi^11^ and were subsequently shortened to a one-pot procedure with either MESNa or MPAA as external thiols (procedures B and C, respectively). We employed the thioesterification method with MESNa (procedure B) for short peptides as it was easier to remove its excess by SPE. Furthermore, the incubation of the peptide precursor in phosphate buffer containing MPAA and TCEP for 48 h leads to the desulphurization of the cyclized product. However, procedure B has not worked for the long precursors of **4** and **5**; therefore, we switched to the more reactive MPAA (procedure C) and replaced phosphate with TEAB (triethylammonium bicarbonate) buffer. In procedure B1 we did not add any thiol. Additionally, the one-pot peptide cyclization was performed in solution (procedure D). The best results were obtained for procedures A–C, which were applied for the syntheses of target peptides **1**–**5**, and the corresponding cyclization yields are listed in Table 1. The obtained peptides were desalted, purified by preparative RP-HPLC, and subsequently analyzed by LC-MS. The cyclization yields were calculated by comparing the amount of the purified peptide to the precursor substitution level determined spectroscopically.^32^ The overall yields were calculated, corresponding to the initial resin loadings.

**Table 1:**
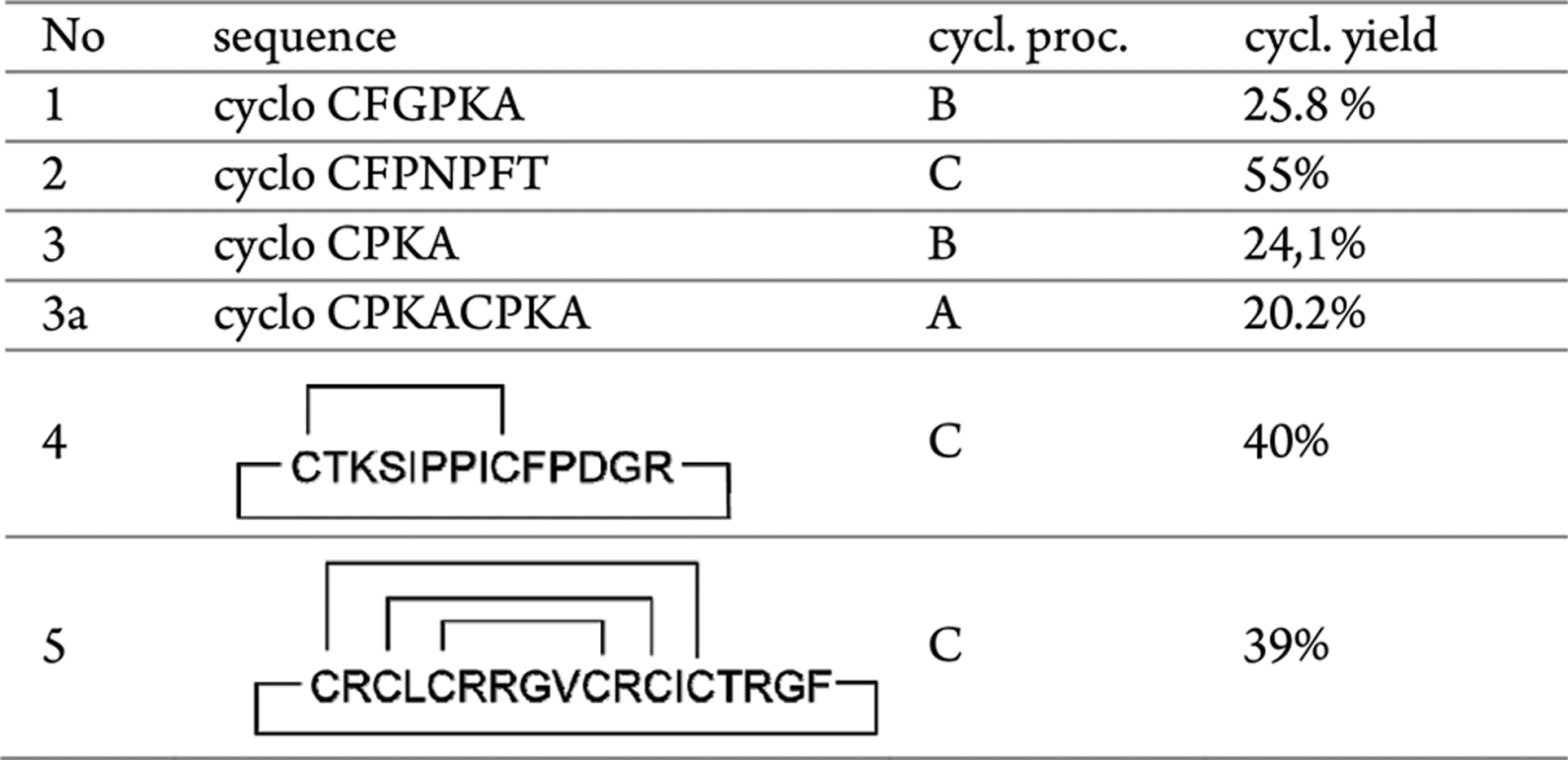
Characterization of Synthesized Peptides

First, we performed the syntheses of model cyclic peptides with 4–7 amino acid residues. When studying the cyclization conditions for the model peptide CFGPKA, we observed that not only the intermediate H–CFGPKA–MESNa (**1a**) was produced after the precursor treatment with MESNa in citrate buffer at pH 3 overnight but also the final cyclized product (**1**). This proved that in the case of peptide precursors with *N*-terminal cysteine, the cyclization is a one-pot process. The isolated thioester dissolved in water underwent self-ligation to **1** and hydrolysis to linear H–CFGPKA–OH **1b** (Figure S24), while when incubated in phosphate buffer (pH 7.4) with DTT at 40 °C it underwent a complete transformation into **1** (Figure S25). Eventually, we established procedure B for the full cyclization of **1**, where the peptidyl resin was first incubated in citrate buffer with 50 equiv of MESNA at 40 °C for 24 h. Then, 10 equiv of DTT in phosphate buffer was added, and the mixture was incubated in the same conditions, which provided the cyclization yield of 26%. The corresponding LC-UV-MS data are presented in SI 6.1. The next investigated peptide was the cysteine analog of axinellin A (**2**), a cytotoxic peptide isolated from marine sponges^33^ (Figure 1). Here we report the data for the synthesis with MPAA in the TEAB buffer medium (procedure C) that yielded 55% of **2**. The final workup involved the acidification with TFA and an extraction with diethyl ether to remove the excess MPAA and DTT. Moreover, because of the usage of a volatile buffer, there was no need for additional desalting, and only lyophilization was sufficient.

Native chemical ligation is considered a racemization-free process. To evaluate epimerization in the *N*-2-[thioethyl]glycine system, we have synthesized [d-Thr^1^,Cys^5^]axinellin A (**2a**) and compared its retention time in LC-MS with that of its l-Thr isomer. This configuration change altered the retention time by more than 1 min, allowing the detection of the epimerized product, which in our case was below 1% (Figure S30*)*.

The cyclic hexa- and heptapeptides were relatively easy to synthesize; however, the formation of the cyclic tetrapeptide was accompanied by the formation of the cyclic octapeptide. In the case of CPKA, a large volume of solvent in the second reaction step (10 mL for 5 mg of peptidyl resin) allowed the formation of cycloCPKA **3**, while a lower volume (2 mL) favored the cyclodimerization toward the cyclic octapeptide with a doubled sequence, cycloCPKACPKA **3a**. The separation and analysis of the cyclic monomer and dimer were challenging since both peptides underwent fast oxidation, producing inter- and intramolecular disulfide bonds. **3a** and oxidized **3** are structural isomers with the same *m*/*z* value and similar retention times (rts); thus, during purification and analysis, a 10 mM TCEP solution was added to maintain the reduced form. Therefore, in section SI 6.4.1 we present the analytical data for both forms. Both peptides were obtained with yields of around 20%. To exclude the possible formation of a cyclodimer, we tested the self-ligation without any additional thiol. This approach gave the final product **3** (Figure S36), thus confirming the direct on-resin self-ligation. This result brought us to reconsider the role of an external thiol in cyclization. Therefore, we compared the susceptibility for cleavage in two sequences: AAAAA, which has no *N*-terminal cysteine, and CPKA. This experiment demonstrates that our device works well for peptide cyclization even without an additional thiol, although it is less efficient for thioester formation in heterogeneous conditions (Figure S18).

Switching to longer bioactive peptides, we synthesized the sunflower trypsin inhibitor SFTI-1 (**4**, Figure 1).^34^ This bicyclic peptide contains two cysteine residues and can be synthesized using two different *N*-terminal cysteine peptide precursors. We chose the precursor sequence CTKSIPPICFPDGR-*N*-2-[thioethyl]glycine-linker to avoid the less-reactive *C*-terminal Ile in a sequence. Additionally, the proline residues located in the middle of the chain facilitate the β-turn formation. The purified monocyclic **4** was then subjected to air oxidation for 48 h to form the disulfide bridge. The natively oxidized product is predominant; however, we also observed traces of intermolecular disulfide bond formation (Figures S41–S42). In addition, we also synthesized the SFTI-1 precursor using ChemMatrix resin with a Rink linker. This allowed for the TFA-based cleavage of the whole construct, including the linker. Then, we performed the cyclization in solution according to the aforementioned procedure D. This experiment, in turn, enabled an insight into the degree of substrate conversion. LC-MS analysis revealed relatively high conversion (80%) of the substrate toward the cyclic product (Figure S45). The identity of the obtained bicyclic SFTI-1 was confirmed by a direct LC-MS comparison with a reference sample provided by Rolka,^35^ and both LC-MS and MS^2^ spectra gave identical fragmentation patterns (Figure S44).

Following the presented approach, we also synthesized the rhesus Θ-defensin RTD-1 (**5**, Figure 1), which is a cysteine-rich antimicrobial peptide that plays a significant role in the mammalian innate immune system. We chose to form a Phe–Cys peptide bond through NCL and therefore located the Phe residue on the *C*-terminus of the linear precursor (CRCLCRRGVCRCICTRGF-*N*-2-[thioethyl]glycine-linker). It was proven that ligations at the *C*-terminal Phe proceed efficiently.^36^ The cyclization yield of peptide **5** was almost 40%. The last step involved oxidation to form three disulfide bonds. The LC-MS data of RTD-1 are presented in Figure 2. An intramolecular disulfide bond arrangement for the oxidized defensin has not been tested, however, literature data clearly show that the oxidation of the cyclic precursor of Θ-defensin results in the formation of the native configuration of disulfide bridges. We applied the oxidation method of Conibear^37^ that provided a one sharp chromatographic peak corresponding to the molecular mass of oxidized Θ-defensin. Therefore, we assumed that the structure of the obtained product was identical to that of the native one.

**Figure 2:**
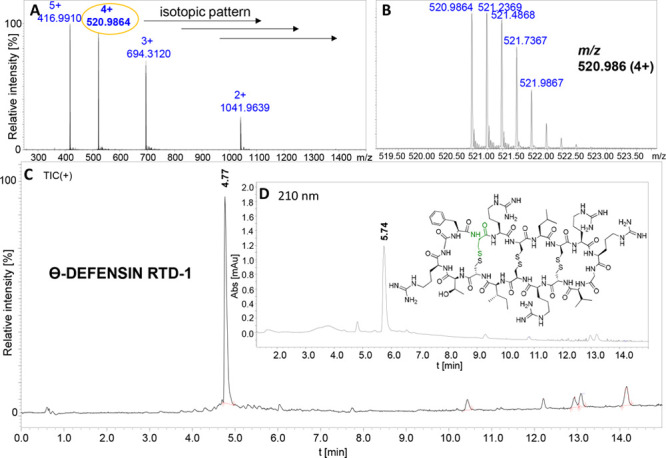
Analytical data obtained for purified and oxidized Θ-defensin RTD-1 **5**. (A) LC-ESI-MS spectrum. (B) MS spectrum with the expanded isotopic pattern of [M + 4H]^4+^: *m*/*z* 520.9864. (C) LC-MS chromatogram (total ion current, TIC), LC-ESI-qTOF-MS instrument. (D) HPLC chromatogram at 210 nm, rt = 4.77 min, LC-UV-ESI-IT-TOF instrument.

To conclude, we have demonstrated that the *N*-2-[thioethyl]glycine moiety, which undergoes a *N,S*-acyl shift, can be applied for the synthesis of cyclic peptides containing cysteine residues. Through the one-pot cyclization, the yield of the synthesis can be improved as it avoids the isolation and purification of unstable thioesters. We also confirmed that the proposed method is essentially epimerization-free. In addition, the synthesis of *N*-2-[thioethyl]glycine is straightforward and can be easily assembled directly on a polymeric support using inexpensive and commercially available reagents. Our linker can be further efficiently derivatized directly on the resin both manually and automatically. Presented studies suggest that both the following mechanisms take place: in the first step, the peptide thioester is released from the solid support as a result of the *N,S*-acyl transfer and transthioesterification, which next undergoes the intramolecular NCL reaction to result in the formation of a homodetic cyclic peptide. The second mechanism proceeds by direct *N*-terminal cysteine self-ligation without an external thiol. Our methodology simplifies the synthesis of biologically relevant cyclic peptides over protocols described in the literature.

## Experimental Section

### General

Solid-phase peptide synthesis according to the FMOC strategy (Fmoc SPPS) was performed manually with an ultrasound-assisted coupling method developed in our research group^31^ in syringe reactors equipped with filters (Intavis). Automated peptide synthesis was performed on a Microwave Peptide Synthesizer Initiator+ Alstra (Biotage, Sweden) instrument. For desalting and solid-phase extraction, either Sep-Pak C18 Plus short cartridges (360 mg sorbent, 55–105 μm particle size, 125 Å pore size, pH 2–8, Waters) or OMIX C18 pipet tips for microextraction (10–100 μL, Varian) for microscale monitoring were used. The synthesized peptides were purified using preparative HPLC on a Varian ProStar (Palo Alto, CA) spectrometer equipped with the UV detector at 210 and 280 nm, with the column TSKgel ODS-120T (215 × 30 mm, 150 Å, 10 μm), a flow rate of–7 mL/min, and the following eluents: A = 0.1% TFA in H_2_O and B = 0.1% TFA in 80% MeCN/H_2_O, with a gradient of 0–40% B/A in 40 min. For peptide characterization, an analytical Thermo Separation HPLC system with UV detection (210 nm) was used with a Vydac Protein RP C18 column (4.6 × 250 mm, 5 μm) and a gradient elution of 0%–80% B/A in 40 min (A = 0.1% TFA/H_2_O and B = 0.1% TFA in 80% MeCN/H_2_O, flow rate of 1 mL/min) as well as HRMS via an ESI-FT-ICR Apex-Qe 7T instrument (Bruker) in the positive ion mode. For fragmentation, the collision-induced dissociation (CID) technique was used with argon as the collision gas. The potential between the spray needle and the orifice was set to 4.5 kV. The MS was calibrated with a Tunemix mixture (Bruker Daltonics) following a quadratic method. Samples were dissolved in 0.1% HCOOH in 50% MeCN/H_2_O. Additionally, for **6** and **6a**, a NaCl solution was added to the final concentration of 10^–4^ mmol. LC-MS experiments were performed on Shimadzu LCMS-9030 and Shimadzu LC-UV-IT-TOF instruments in the positive ion mode with electrospray ionization. The LCMS-9030 instrument was equipped with a UHPLC Nexera X2 system and a hybrid mass analyzer, which was a single quadrupole coupled with time-of-flight mass analyzer (qTOF) at the *m*/*z* range of 100–1500 and a range of 100–2000 for MS/MS. The LC-UV-IT-TOF instrument is a hybrid system consisting of a liquid chromatograph, a PDA detector, an ion trap, and a time-of-flight mass analyzer. In both instruments, CID fragmentation (with Argon) was used, and the potential between the spray needle and the orifice was set to 4.5 kV. The LC systems were operated with the following mobile phases: A = 0.1% HCOOH in H_2_O and B = 0.1% HCOOH in MeCN in a gradient separation from 0 to 60% B/A in 15 min at a 0.2 mL/min flow rate and a 2 μL injection. For highly hydrophilic peptides, the isocratic conditions were applied. The separations were performed on an Aeris Peptide XB-C18 column (100 mm × 2.1 mm, 1.7 μm bead diameter). If mentioned, the column was thermostated. More hydrophobic peptide samples (**4**, **5**, and their derivatives) were dissolved in 400 μL of a water/acetonitrile mixture (95:5). ^1^H NMR and ^13^C{^1^H} NMR spectra were recorded on a high field Bruker 500 MHz spectrometer equipped with a broadband inverse gradient probe head. Spectra were referenced to the residual solvent signal (CDCl_3_ at 7.24 ppm or MeOD at 4.87 ppm). The deuterated solvents were purchased from Sigma-Aldrich. For the measurements of the substitution level, a UV–vis Plate reader Tecan infinite M200 Pro instrument was used (Tecan Group Ltd., Männedorf, Switzerland) in the cuvette measurement mode with blanking in the range of 230–700 nm.

### Reagents and Materials

All commercially available reagents were used without further purification, and water was deionized by the reverse osmosis system (Hydrolab, Poland). Solvents for building block synthesis and peptide synthesis are as follows: chloroform (stabilized with amylene), CDCl_3_; dimethylformamide, DMF; dichloromethane, DCM; methanol, MeOH; tetrahydrofuran, THF; diethyl ether, Et_2_O; *N*,*N*-diisopropylethylamine, DIPEA; piperidine, PIP; HCOOH; trifluoroacetic acid, TFA; cyanogen bromide, BrCN; triisopropylsilane, TIS; and 1,2-ethanedithiol, EDT. Solvents in the analytical grade were obtained from Sigma-Aldrich, and acetic anhydride was obtained from Lachner. Fmoc-amino acid derivatives for peptide synthesis were purchased from PeptideWeb, except for Fmoc–Cys(Mmt)–OH and Fmoc–Lys(Mtt)–OH (Sigma-Aldrich). Coupling reagents are as follows: TBTU, *O*-(benzotriazole-1-yl)-*N,N,N*′,*N*′-tetramethyluronium tetrafluoroborate; PyBop, benzotriazole-1-yl-oxytripyrrolidinophosphonium hexafluorophosphate; and PyAOP, 7-azabenzotriazol-1-yloxytrispyrrolidinophosphonium hexafluorophosphate. Reagents were purchased from Navoabiochem, *N,N*′-diisopropylcarbodiimide (DIC) was purchased from Fluka and Oxyma Pure was purchased from Iris Biotech. The resins for SPPS are as follows: H-Rink amide ChemMatrix resin (0.40–0.60 mmol/g) was purchased from Sigma-Aldrich, and TentaGel HL NH2 (0.56 mmol/g and 0.26 mmol/g) and TentaGel MB NH2 (0.23 mmol/g) resins were purchased from RAPP Polymers GmbH. For the *N*-2-[thioethyl]glycine synthesis, cysteamine chloride, 4-methoxytrityl chloride (mmt-Cl), trityl chloride (trt-Cl), chloroacetic acid, and bromoacetic acid were purchased from Sigma-Aldrich. For peptide cyclization, sodium 2-mercaptoethanesulfonate, MESNa, 4-mercaptophenylacetic acid, MPAA, tris(2-carboxyethyl)phosphine, TCEP, dithiothreitol, DTT, triethylammonium bicarbonate buffer, and TEAB (1M, pH 8.5) were purchased from Sigma-Aldrich. Other chemicals, including citric acid (monohydrate), hydrated disodium phosphate, POCH, hydrated monosodium phosphate, were purchased from AppliChem, NaOH was purchased from Stanlab, and analytical grade HCl was purchased from Sigma-Aldrich. Solvents for LC-MS are as follows: MeCN and HCOOH in HPLC grade were purchased from Sigma-Aldrich, and MeOH and HPLC grade H_2_O in HPLC were purchased from J. T. Baker.

### Synthesis of Protected Cysteamines **6** and **6a**

Both *S*-protected cysteamines with trityl and 4-methoxytrityl groups were synthesized according to the procedure adopted from Barlos et al.^30^ as described for 4-methoxytrityl-*S*-cysteine in a mild DMF medium.

#### Synthesis of 2-[(Triphenylmethyl)sulfanyl]ethan-1-amine **6**

Cysteamine hydrochloride (5.68 g 50 mmol) was dissolved in 50 mL of DMF in an Erlenmeyer flask equipped with a magnetic stirrer , and to the flask was added 13.94 g (50 mmol) of trityl hydrochloride. The mixture was stirred at room temperature for 3 h. The resulting solution was concentrated under the stream of nitrogen, then neutralized with KOH/MeOH to a pH of 9 and evaporated overnight under the stream of nitrogen. The resulting mixture was extracted four times with DCM (4 × 50 mL), then all fractions were collected, washed with water (2 × 25 mL) and brine (2 × 25 mL), dried over MgSO_4_, and evaporated on a rotary evaporator. The final product was crystallized from EtOAc by adding small portions of hexane. Crystals were left for growth overnight at 4 °C; yield: 8.07 g (25.27 mmol −50.54%) of a yellowish powder. TLC: 5% MeOH/CHCl_3_, rf = 0.29, mp = 92–94 °C. HRMS (ESI-FT-ICR) *m*/*z*: [M + Na]^+^ Calcd for C_21_H_21_NSNa^+^ 342.129, found 342.125. ^1^H NMR (MeOD, 500 MHz): δ 7.43 (dt, 6H, *J* = 8.6, 2.4 Hz), 7.31 (t, 6H, *J* = 7.6 Hz), 7.24 (t, 3H, *J* = 7.3 Hz), 2.46 (t, 2H, *J* = 7.0 Hz), 2.37 (t, 2H, *J* = 7.3 Hz). ^13^C{^1^H} NMR (CDCl_3_, 150 MHz): δ 145.1, 129.8, 128.0, 126.8, 66.7, 41.2, 36.4.

#### Synthesis of 2-{[(4-Methoxyphenyl)(diphenyl)methyl]sulfanyl}ethan-1-amine **6a**

Cysteamine hydrochloride (1.136 g 10 mmol) was dissolved in 5 mL of DMF in an Erlenmeyer flask equipped with a magnetic stirrer, and to the flask was added 3.08 g (10 mmol) of 4-methoxytrityl hydrochloride. The mixture was stirred at room temperature for 3 h. The resulting solution was concentrated under the stream of nitrogen, then neutralized with a 1 M NaOH water solution to a pH of 9 and evaporated under the stream of nitrogen overnight. The resulting mixture was extracted four times with DCM (4 × 25 mL), then all fractions were collected, washed with water (2 × 25 mL) and brine (2 × 25 mL), dried over MgSO_4_, and evaporated on a rotary evaporator. A very dense and viscous yellow-orange oil was obtained, and several solvents for crystallization were tested (ethyl acetate, acetone, CHCl_3_, DCM, THF, and butanol with *n*-hexane); however, no crystal form appeared. The final mixture was purified by column chromatography on silica using 5% MeOH/CHCl_3_; yield: 1.48 g (4.24 mmol, 42.35%). TLC: 5% MeOH/CHCl_3_, rf = 0.19. HRMS *m*/*z*: [M + Na]^+^ Calcd for C_22_H_23_NOSNa^+^ 372.139, found 372.133. ^1^H NMR (MeOD, 500 MHz): δ 7.45–7.42 (m, 4H), 7.35–7.32 (m, 2H), 7.31–7.28 (m, 4H), 7.24–7.21 (m, 2H), 6.87–6.84 (m, 2H), 3.79 (s, 3H), 2.47 (t, 2H, *J* = 7.2 Hz), 2.39 (t, 2H, *J* = 6.5 Hz); ^13^C{^1^H} was reported by Riddoch.^29^

### Manual On-Resin Formation of *N*-2-[Thioethyl]glycine by the Incorporation of Protected Cysteamines

In a syringe reactor was placed 200 mg of resin, which was swelled for 30 min at room temperature in DMF. For **1**, **2**, and **3a**, TentaGel HL NH_2_ resin (0.56 mmol/g) was used; for **3**, TentaGel HL NH_2_ resin (0.26 mmol/g) was used; and for **4** and **5**, TentaGel S NH_2_ resin (0.23 mmol/g) was used. Additionally, **4** was also synthesized on ChemMatrix resin (0.4–0.6 mmol/g). A linker containing either AAM (for **1**, **3**, and **3a**) or a βAβAM-sequence (for **2**, **4**, and **5**) was synthesized according to the standard Fmoc-SPPS strategy. For the coupling, 3 equiv of TBTU or PyBOP, 6 equiv of DIPEA, and 3 equiv of the corresponding Fmoc-protected amino acid in 1 mL of DMF were poured into the syringe, and the mixture was placed for 20 min in the ultrasonic bath. After filtrating and washing the peptidyl resin five times with DMF, 25% PIP/DMF was poured into the mixture for Fmoc deprotection, and the resin was stirred in the same way for 5 min. After this time, the reagent was filtered out, and the peptidyl resin was washed seven times with DMF. Next, bromo- or chloroacetic acid was added (5 equiv) with 5 equiv of DIC in 1 mL of DMF three times. The mixture was stirred for 30 min on a rotary mixer, followed by filtrations and washing seven times with DMF. Then, 6 equiv of the protected cysteamine was added in 1 mL of DMF, and the mixture was stirred overnight at room temperature on a rotary mixer. Next, the peptidyl resin was filtered and washed five times with DMF. The reaction steps were monitored with both Kaiser and chloranil tests. After synthesis, the peptidyl resin was washed with DMF/DCM, DCM, DCM/THF, THF, and THF/Et_2_O and dried in a desiccator. For monitoring, 10 mg of *N*-2-[(trt)thioethyl]glycine-AAM-TentaGel was incubated in 20 μL of 3 M BrCN/DCM plus 200 μL of 70% HCOOH/H_2_O overnight. The filtrate was evaporated under the stream of nitrogen, dissolved in water with 10% MeCN, and directly measured by LC-UV-MS. rt for *N*-2-[(trt)thioethyl]glycine-AA-HL: 11.8–12 min (1–60% B/A in 15 min). HRMS (ESI-IT-TOF) *m*/*z*: [M + H]^+^ Calcd for C_33_H_39_N_4_O_5_S 603.2635, found 603.2639. rt for [N-2-[thioethyl]glycineAA-HL]_2_: 3.6–3.8 min, *m*/*z* [M]^2+^ Calcd for C_28_H_48_N_8_O_10_S_2_ 360.1462, found 360.1450 (HL = homoserine lactone).

### Manual Peptide Precursor Synthesis on Peptidyl Resin and Its Activation

The syntheses of peptide precursors were performed on the *N*-2-[(trt)thioethyl]glycine-AAM-resins or *N*-2-[(trt)thioethyl]glycine-βAβAM-resins described above according to the same standard Fmoc strategy. The only exception was the coupling of the next Fmoc-protected amino acid to the *N*-2-[(trt)thioethyl]glycine residue. In this case, 6 equiv of the Fmoc-protected amino acid was added twice with 6 equiv of PyAOP and 12 equiv of DIPEA. After the coupling of the last amino acid residue (cysteine), the Fmoc was not removed, and the peptidyl resin was dried and placed in the desiccator. Then, 5 mg of the resin with the peptide precursor was swelled in 1 mL of 20% PIP/DMF for 20 min to determine the substitution level (see below Fmoc Substitution Level Determination). Next, the dried peptidyl resin was subjected to the final deprotection and cleavage with 20 μL of 3 M BrCN/DCM plus 200 μL of 70% HCOOH/H_2_O overnight. The filtrate was evaporated under the stream of nitrogen and lyophilized. Then, it was dissolved in 0.1% HCOOH in MeCN/H_2_O (1:1) and measured by FT-ICR-MS. After proving the completeness of the synthesis and determining the substitution level, the whole peptidyl resin was subjected to Fmoc deprotection, and side protecting groups were removed using 1 mL of a mixture of TFA/H_2_O/TIS/EDT (94:2:2:2) for 2 h. Then, the solution was filtered out, the peptidyl resin was washed three times with DCM and neutralized with 5% DIPEA/DMF (three washing steps for 5 min each), and the standard drying procedure was applied.

### Automated Microwave-Assisted on-Resin Formation of *N*-2-[Thioethyl]glycine and the Further Peptide Precursor

#### Partially Automated Microwave-Assisted Synthesis

TentaGel MB NH2 resin with incorporated *N*-2-[(trt)thioethyl]glycine-AAM or *N*-2-[(trt)thioethyl]glycine-βAβAM was prepared manually as mentioned above and placed in the microwave reactor of the Biotage Initiator+ Alstra peptide synthesizer. For the first coupling, 6–8 equiv of Fmoc-Aa, DIC, and Oxyma Pure were used two times for 10 min in 75°. For the next couplings, the excess was reduced to 5 equiv and the reaction time to 5 min at once. All reagents were used in a concentration of 0.2 M in DMF. For Fmoc deprotection, 4.5 mL of 20% PIP/DMF was added two times for 3 and 10 min at rt with oscillating mixing.

#### Fully Automated Microwave-Assisted Synthesis

For the fully automated synthesis of peptide precursors with *N*-2-[(trt)thioethyl]glycine with a linker, several conditions for *S*-trityl-cysteamine incorporation were studied and are presented in Table S1. The tested sequence was AAAAA-*N*-2-[(trt)thioethyl]glycine-AAM on a TentaGel MB NH2 solid support, which was cleaved by the reaction with 20 μL of 3 M BrCN/DCM plus 200 μL of 70% HCOOH/H_2_O overnight. For comparison, conditions for the fully manual and partially automated syntheses are also listed.

### Fmoc Substitution Level Determination

For the determination of the ε value for the dibenzofulvene-piperidine adduct, 7.77 mg of Fmoc–Phe–OH (20,05 μmol) was dissolved in 1 mL of 20% PIP/DMF, and the mixture was incubated for 20 min at room temperature. Then, the volume was adjusted to 10 mL, the absorbance spectrum was recorded after dilution, and the reference maximum of absorption was chosen as 290 nm according to the recommendations of Bachem.^32^ For comparison, we also recorded the UV–vis spectra of Fmoc–OSu and Fmoc–Ala–OH, which were treated in the same way. A series of dilutions was prepared, and the absorption was measured for the calibration curve. Pure 20% PIP/DMF was used as a blank. The determined extinction coefficient was ε_290 nm_ = 5597.1 l/mol·cm, and ε_301 nm_ = 6789.5 l/mol·cm. Then, 5 mg of the examined peptidyl resin was swelled directly in 20% piperidine/DMF for 20 min. After filtration, the resin was washed three times with 1 mL of the same solution, and all rinses were collected together and diluted to 10 mL. Then, the sample was diluted 10 more times and measured in a quartz cuvette, with 20% PIP/DMF a blanking. The determination of the substitution level was repeated at least two times. Substitution level was calculated from the ε_290_ value and dilution factor according to the equation:
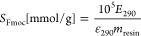


### Peptide Cyclization Procedures

#### Procedure A

Through double on-resin transthioesterification (MESNa then MPAA) (Table 2): 200 mg of resin with an unprotected peptide precursor was swelled in 1 mL of citrate buffer (pH 3). Then, corresponding to the resin loading, 50 equiv of MESNa was added in a volume of citrate buffer that gave the final peptide concentration of 3 mmol. The reaction mixture was incubated in a water bath (40 °C) for 24 h and mixed manually from time to time. Next, an additional solution of 50 equiv of MPAA with 10 equiv TCEP in the same amount of phosphate buffer (pH 7.4) was poured into the mixture. Then, the pH of the final solution was adjusted with 8 M NaOH to the value of 7.4, and the reaction mixture was incubated at 40 °C for the next 24 h. After this time, the syringe content was filtered out and desalted by SPE. The fractions containing the desired product were evaporated under the stream of nitrogen and lyophilized.

**Table 2:** Procedures Tested for the Peptide One-Pot Cyclization

procedure	external thiol	buffer/pH	on-resin or in-solution
A	(1) MESNa	(1) citrate/3	on-resin
	(2)MPAA	(2) phosphate/7.4	
B	MESNa	(1) citrate/3	on-resin
		(2) phosphate/7.4	
B1		citrate/3	on-resin
C	MPAA	TEAB/7.0	on-resin
D	MPAA	TEAB/7.0	in-solution

#### Procedure B

Through a single on-resin transthioesterification with MESNa: 200 mg of resin with an unprotected peptide precursor was swelled in 1 mL of citrate buffer (pH 3). Then, corresponding to the resin loading, 50 equiv of MESNa was added in a volume of citrate buffer that gave the final peptide concentration of 3 mmol. The reaction mixture was incubated in a water bath (40 °C) for 24 h and mixed manually from time to time. To increase the reaction yield, the next 10 equiv of DTT in phosphate buffer was added to the mixture. The pH was adjusted to 7.4 with 8 M NaOH, and the syringe was placed into the water bath for the next 24 h in 40 °C. After this time, the syringe content was filtered out and desalted by SPE. The fractions containing the desired product were evaporated under the stream of nitrogen and lyophilized.

#### Procedure B1

Self-cyclization without any additional thiol: 100 mg of resin with the unprotected peptide precursor was incubated in 10 mL of citrate buffer at pH 3 with 10 equiv of DTT in the water bath for 24 h at 50 °C, and the mixture was mixed manually from time to time. After this time, the syringe content was filtered out and desalted by SPE. The fractions containing the desired product were evaporated under the stream of nitrogen and lyophilized.

#### Procedure C

Through a single on-resin transthioesterification with MPAA: 20 mg of resin with the unprotected peptide precursor was placed in an Eppendorf tube and suspended in a 200 μL solution of 50 equiv of MPAA and 15 equiv of DTT in 0.5 mL of 1 M TEAB buffer, with the final pH adjusted to 7. The obtained mixture was heated at 50 °C over 48 h. After that time, a few droplets of concentrated trifluoroacetic acid were added to acidify the solution. This in turn resulted in the precipitation of MPAA, which was then extracted with diethyl ether (3 × 50 μL). To remove the volatile buffer, the mixture was lyophilized.

#### Procedure D

Through transthioesterification in solution: 2 mg of the linear peptide precursor was placed in an Eppendorf tube. The next stages were carried out analogously as those described for procedure C.

### Synthesis of the Model Peptide Cyclo-CAKPGG Containing Different Linkers and Study on Their Impact on the Final Cyclization

Four different linkers were synthesized manually. The synthesis was carried out only on an analytical scale (20 mg of TentaGel MB NH_2_ resin −0.23 mmol/g). To confirm the designed structures, only 3 mg of peptidyl resins CAKPGG-*N*-2-[thioethyl]glycine-linker-resin were treated with cyanogen bromide (20 μL of 3 M BrCN/DCM plus 200 μL of TFA/H_2_O/TIS (95:2.5:2.5)) to cleave the whole peptide construct from the solid support. In the second experiment, 5 mg of CAKPGG-*N*-2-[thioethyl]glycine-linker-resin was subjected to cyclization Procedure C with no further purification. Each product was then dissolved in the same volume of water and analyzed by LC-MS using identical injection volume. The results are presented in SI 4.

#### Optimization of the Transthioesterification Reaction Time

The AAAAA-*N*-2-[thioethyl]glycine-AAM-TentaGel peptide precursor was synthesized partially automated. After incubating the portions (10 mg) of the peptidyl resin in citrate buffer (pH 3, 300 μL) with MESNa (50 equiv, 8.5 mg) after different periods in a 40 °C water bath, the filtrates were collected, desalted by omix tips, lyophilized, dissolved in 0.5 mL of water, and measured by LC-UV-ESI-MS. Similarly, the remained resins were dried and treated with cyanogen bromide (20 μL of 3 M BrCN/DCM plus 200 μL of 70% HCOOH/H_2_O overnight). The filtrates were evaporated, lyophilized, dissolved in 1 mL of water, and measured in the same way. The graph showing the percentage of the final product in time is shown in Figure S16. For the transthioesterification reaction, the final products are the H-AAAAA-MESNa thioester (LC-UV-IT-TO;, rt = 4.8–4.9 min (1–60% B/A in 15 min), HRMS (IT-TOF) *m*/*z* [M + H]^+^ Calcd for C_17_H_32_N_5_O_8_S_2_ 498.1687, found 498.1693) and the unreacted peptidyl resin AAAAA-*N*-2-[thioethyl]glycine-AA-HL (LC-UV; rt = 5.9–6.1 min).

### Synthesized Peptides

For each peptide, the most efficient synthetic procedure is described in the preparative scale with purification, and the compared procedures are described on an analytical scale. Some co- and byproducts are also described.

#### Synthesis of Cyclo-CFGPKA **1**

CFGPKA-*N*-2-[thioethyl]glycine-AAM-TentaGel HL NH_2_ (400 mg, final loading of 0.16 mmol/g) was subjected to cyclization procedure B. The crude product was purified by HPLC, yielding 10.21 mg of the peptide (white powder, 17 μmol, cyclization yield of 25.8%, final yield of 15.9%). LC-UV-MS: rt = 6.6–6.9 min (5–60% B/A in 15 min). HRMS (ESI-IT-TOF) *m*/*z*: [M + H]^+^ Calcd for C_28_H_42_N_7_O_6_S 604.2911, found 604.2914.

#### Synthesis of the 2-Mercaptoethanesulfonate Thioester of CFGPKA **1a**

CFGPKA-*N*-2-[thioethyl]glycine-AAM-TentaGel HL NH_2_ (5 mg) was subjected to the first step of cyclization procedure B and thus was only incubated with 50 equiv of MESNa in citrate buffer (pH 3) for 24 h at 40 °C and desalted. HPLC: rt = 23.5–24.0 min (gradient: 0–80% B/A in 40 min). HRMS (ESI-FT-ICR) *m*/*z*: [M + H]^+^ for C_30_H_48_N_7_O_9_S_3_ Calcd 746.2670, found 746.2323. One aliquot was incubated in deionized water for several days and measured by Thermo analytical chromatography, and the second one was incubated with 10 equiv of DTT in phosphate buffer (pH 7.4) at 40° at room temperature, desalted, and lyophilized.

#### Synthesis of [Cys^5^]-axinellin A **2**

CFPNPFT-*N*-2-[thioethyl]glycine-AAM-TentaGel HL NH2 (20 mg, final loading of 0.18 mmol/g) was subjected to cyclization procedure C. The crude product was purified by HPLC, yielding 1.37 mg of the peptide (white powder, 1.6 μmol, cyclization yield of 55.0%, final yield of 38.1%). LC-MS: rt = 9.08 min. (5–60% B/A in 15 min). HRMS (ESI-qTOF) *m*/*z*: [M + H]^1+^ Calcd for C_39_H_51_N_8_O_9_S 807.349, found 807.354.

#### Synthesis of [d-Thr^1^,Cys^5^]axinellin A **2a**

The synthesis of the peptide precursor was performed in a partially automated way. For the d-Thr coupling, 6 equivs of Fmoc–d-Thr(OtBu)–OH, DIC, and Oxyma Pure were used and coupled twice within 10 min at 75° C. For cyclization, 90 mg of resin with a precursor was subjected to cyclization procedure C and measured by LCMS-9060 without further purification: rt = 8.72 min. (5–60% B/A in 15 min). HRMS (ESI-qTOF) *m*/*z*: [M + H]^1+^ Calcd for C_39_H_51_N_8_O_9_S 807.349, found 807.354.

#### Synthesis of Cyclo-CPKA **3**

CPKA-*N*-2-[thioethyl]glycine-AAM-TentaGel MB NH_2_ (140 mg, final loading: 0.21 mmol/g) was placed in syringe tubes in 10 mg portions and subjected to cyclization procedure B. The modification was applied in the following amount of phosphate buffer: 10 mL was added to each syringe. After cyclization, all fractions were collected together, desalted, and lyophilized. To the crude product was added 0.5 mg of TCEP, and the mixture was incubated for 2 h at 50° C and purified by preparative HPLC (Varian), yielding in 2.83 mg of the peptide (yellow oil, 7.1 μmol, cyclization yield of 24.1%, final yield of 22.0%). HPLC: rt = 2.8–3.2 min (1% in 8 min, 1–10% in 10 min, 10% in 10 min, 10–100% in 6 min B/A). HRMS (ESI-IT-TOF) *m*/*z*: [M + H]^+^ Calcd for C_17_H_30_N_5_O_4_S 400.2013, found 400.2016.

#### Synthesis of Cyclo-CPKA **3** with No Thiol Addition

100 mg of CPKA-N-[2-thioethyl]glycine-AAM-TentaGel NH2 (final loading of 0.21 mmol/g) was placed in portions in a syringe tube and subjected to the cyclization *Procedure B1*. The modification was applied in the amount of phosphate buffer: 10 mL was added. After 24h incubation, the filtrate was desalted, lyophilized, and analyzed by LCMS 9030 (ESI-qTOF).

#### Synthesis of Bicyclo-CPKACPKA **3a**

CPKA-*N*-2-[thioethyl]glycine-AAM-TentaGel MB NH2 (400 mg, final loading of 0.07 mmol/g) was subjected to cyclization procedure A. The crude product was purified by HPLC, yielding in 2.27 mg of a white powder peptide (28 μmol, cyclization yield of 20.23%, final yield of 6.16%). LC-UV-MS: rt = 5.2–5.5 min (1% in 5 min, 5–10% in 10 min, 10% in 5 min, 10–100% in 3 min B/A). HRMS (ESI-IT-TOF) *m*/*z*: Calcd [M + 2H]^2+^ for C_34_H_58_N_10_O_8_S_2_ 399.1935, found 399.1934.

#### Synthesis of SFTI-1 **4**

CTKSIPPICFPDGR-*N*-2-[thioethyl]glycine-AAM-TentaGel MB NH2 (20 mg, final loading of 0.16 mmol/g) was subjected to cyclization procedure C. The crude product was purified by HPLC, yielding in 1.9 mg of a white powdered peptide (1.2 μmol, cyclization yield of 40%, final yield of 27.8%). LC-UV: rt = 8.05 min. (5–65% B/A). HRMS (ESI-qTOF) *m*/*z*: [M + 2H]^2+^ Calcd for C_67_H_106_N_18_O_18_S_2_ 757.3684, found 757.368.

### Synthesis of SFTI-1 **4** with Cyclization In-Solution

CTKSIPPICFPDGR-*N*-2-[thioethyl]glycine-AAM-ChemMatrix (20 mg) was treated with a freshly prepared TFA/H_2_O/TIS/EDT (90/2.5/5/2.5; v/v/v/v) mixture to cleave the peptide precursor from the solid support and purified by preparative HPLC. The obtained linear precursor of SFTI-1 with the *C*-terminal attached cysteamine residue was subjected to the cyclization according to procedure D. LC-MS: rt = 7.41 min. HRMS (ESI-IT-TOF) *m*/*z* [M+2H]^2+^ Cald for C_67_H_108_N_18_O_18_S_2_ (reduced SFTI) 758.376, found 758.381.

#### Synthesis of Θ-defensin RTD-1 **5**

CRCLCRRGVCRCICTRGF-*N*-2-[thioethyl]glycine-AAM-TentaGel MB NH2 (10 mg, final loading of 0.16 mmol/g) was subjected to cyclization procedure C. The crude product was purified by HPLC, yielding in 1.3 mg of the peptide (0.6 μmol, cyclization yield of 39%, final yield of 27.1%). LC-UV: rt = 5.74 min. (5–60% B/A in 1 min). HRMS (ESI-qTOF) *m*/*z*: [M + 4H]^4+^ Calcd for C_82_H_141_N_33_O_19_S_6_ 520.9864, found 520.9849.

## Supporting Information Available

HPLC, UV, NMR, HR-MS, and MS^*n*^ analysis of synthesized products and their intermediates (PDF)
