# Supplementary material for: One-Pot Cyclization and Cleavage of Peptides with N-Terminal Cysteine via the N,S-Acyl Shift of the N-2-[Thioethyl]glycine Residue
Source: J Org Chem. 2021 Aug 6;86(17):12292–9. doi: 10.1021/acs.joc.1c01045 (PMC8419835; doi:10.1021/acs.joc.1c01045)
Supplement: Supplementary file 1 — jo1c01045_si_001.pdf [file jo1c01045_si_001.pdf]

Supplementary information

One-pot cyclization and cleavage of peptides with *N*-terminal Cysteine *via N,S*-acyl shift of *N*-2-[thioethyl]glycine residue

Magdalena Wierzbicka, Mateusz Waliczek, Anna Dziadecka, Piotr Stefanowicz*

Faculty of Chemistry, University of Wrocław, F. Joliot-Curie 14, 50-383 Wrocław, Poland

*Corresponding author: piotr.stefanowicz@chem.uni.wroc.pl

Table of content

1. Trt and mmt protected Cysteamine 6 and 6a — S2

*1.1. 2-[(triphenylmethyl)sulfanyl]ethan-1-amine **6** — S2*

*1.2. 2-{[(4-methoxyphenyl)(diphenyl)methyl]sulfanyl}ethan-1-amine **6a** — S4*

2. On-resin formation of N-2-[thioethyl]glycine and further peptide precursor synthesis — S5

2.1. Manual synthesis — S5

2.2. Automated microwave-assisted synthesis — S6

3. Fmoc substitution level determination — S8

4. Study on different linkers and their impact on final cyclization of CAKPGG — S9

5. Cleavage of peptide thioesters without N-terminal cysteine — S13

6. Analytical data for the synthesized peptides — S16

*6.1. CycloCFGPKA **1** — S16*

6.2. 2-mercaptoethanesulfonate thioester of CFGPKA **1a** — S20

*6.3. [Cys^5]-axinellin A **2** — S22*

6.3.1. [D-Thr^1,Cys^5]axinellin A **2a** for enantiomerization study — S25

*6.4. Cyclo CPKA **3** — S26*

6.4.1. Cyclization mediated by MESNa transthioesterification — S26

6.4.2. Cyclization without any additional thiol — S30

6.4.3. Bicyclo CPKACPKA **3a** — S31

*6.5. SFTI-1 **4** — S33*

6.5.1. On-resin cyclization — S33

6.5.2. Cyclization in solution — S38

*6.6. Θ-defensin RTD-1 **5** — S39*

1. *Trt and mmt protected Cysteamine **6** and **6a***

*1.1. 2-[(triphenylmethyl)sulfanyl]ethan-1-amine **6***

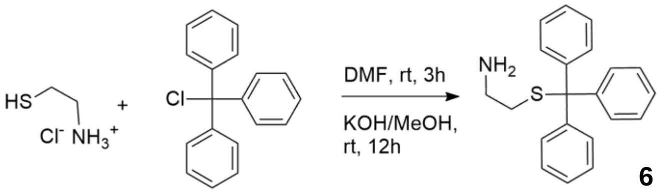

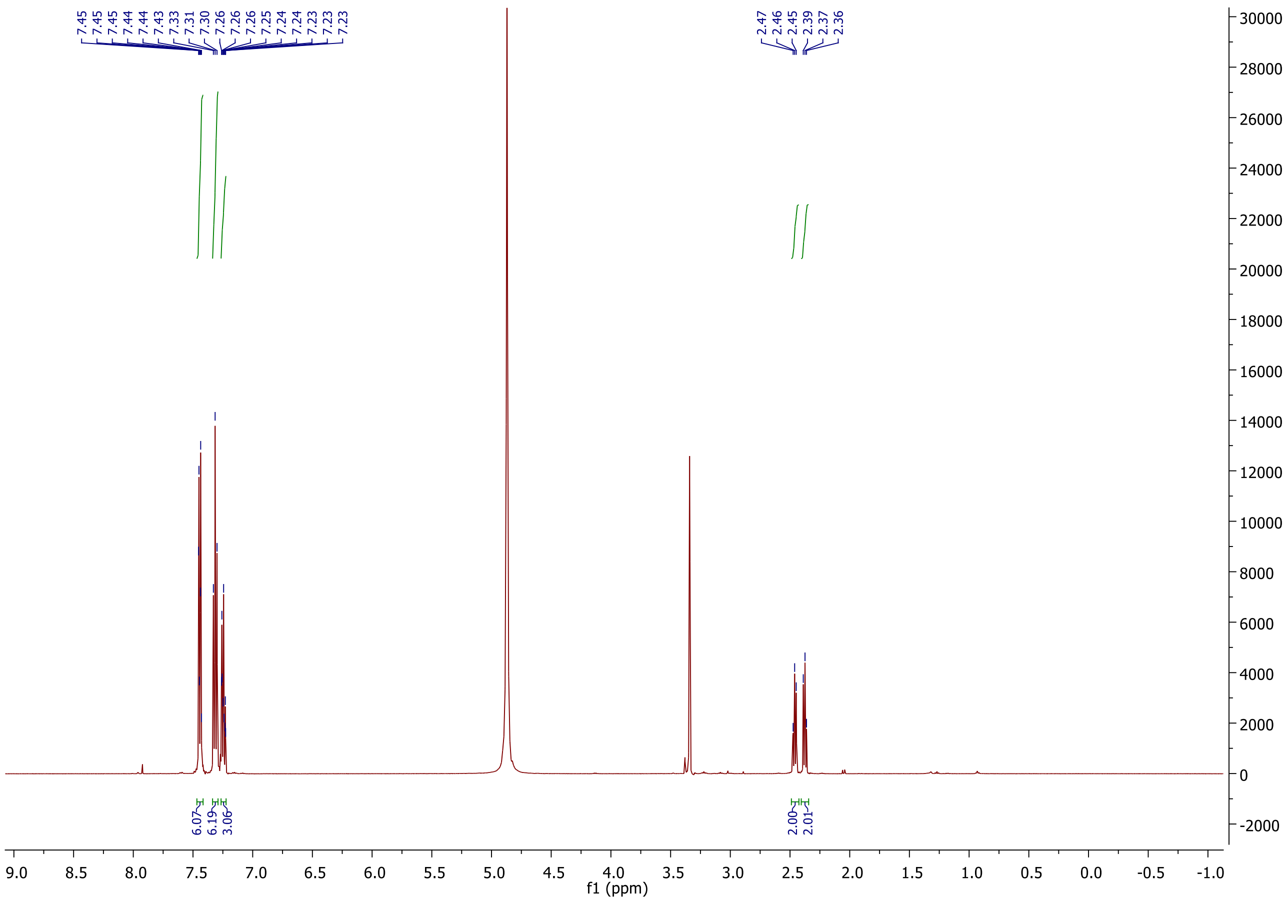

Fig. S 1 ^{1}H NMR spectrum (MeOD, 500 MHz) of S-trityl-cysteamine **6**.

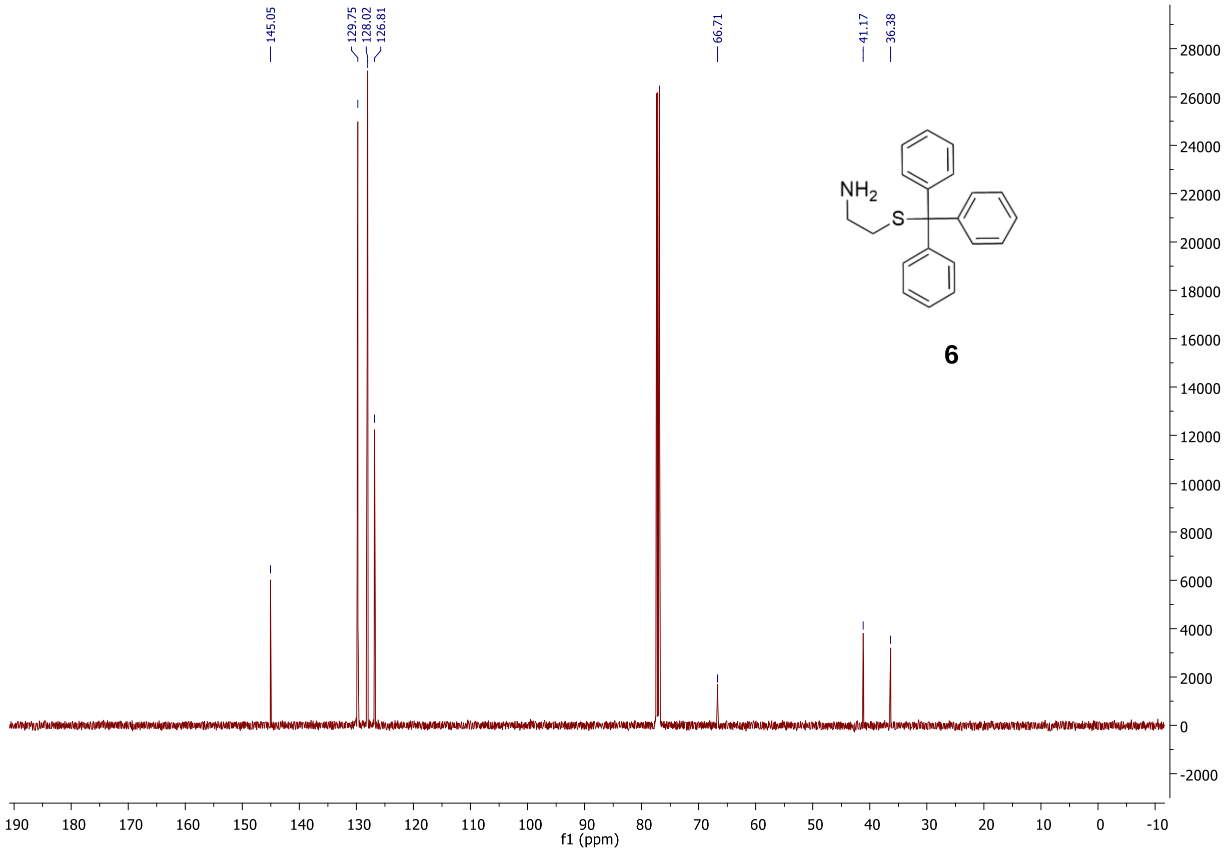

Fig. S 2 $^{13}C\{^1H\}$ NMR spectrum ($CDCl_3$, 150 MHz) of S-trityl-cysteamine **6**.

1.2. 2-{[(4-methoxyphenyl)(diphenyl)methyl]sulfanyl}ethan-1-amine ***6a***

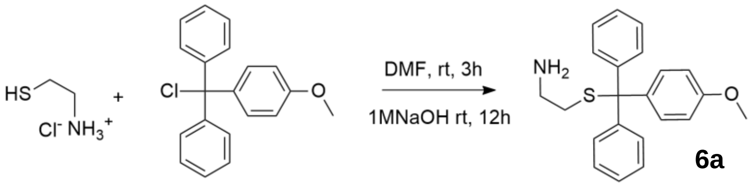

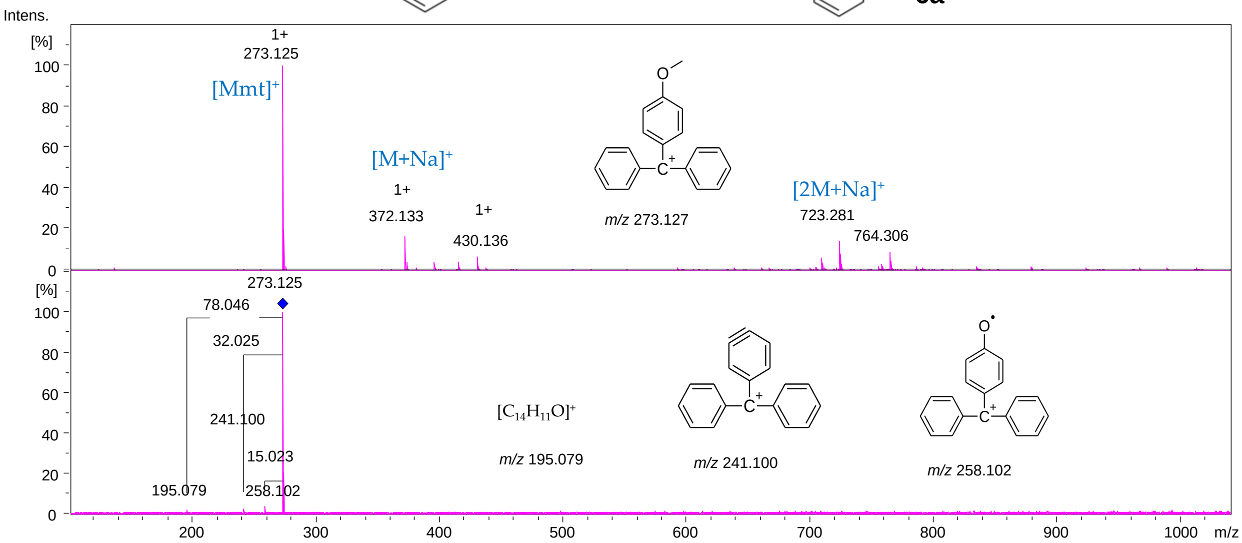

Fig. S 3 ESI-FT-ICR-MS (upper) of S-methoxytrityl-cysteamine **6a** and MS/MS (lower) analysis of [Mmt]$^+$ parent ion (p.i) – 273.125, CE = 15 eV with the presented structures of fragment ions.

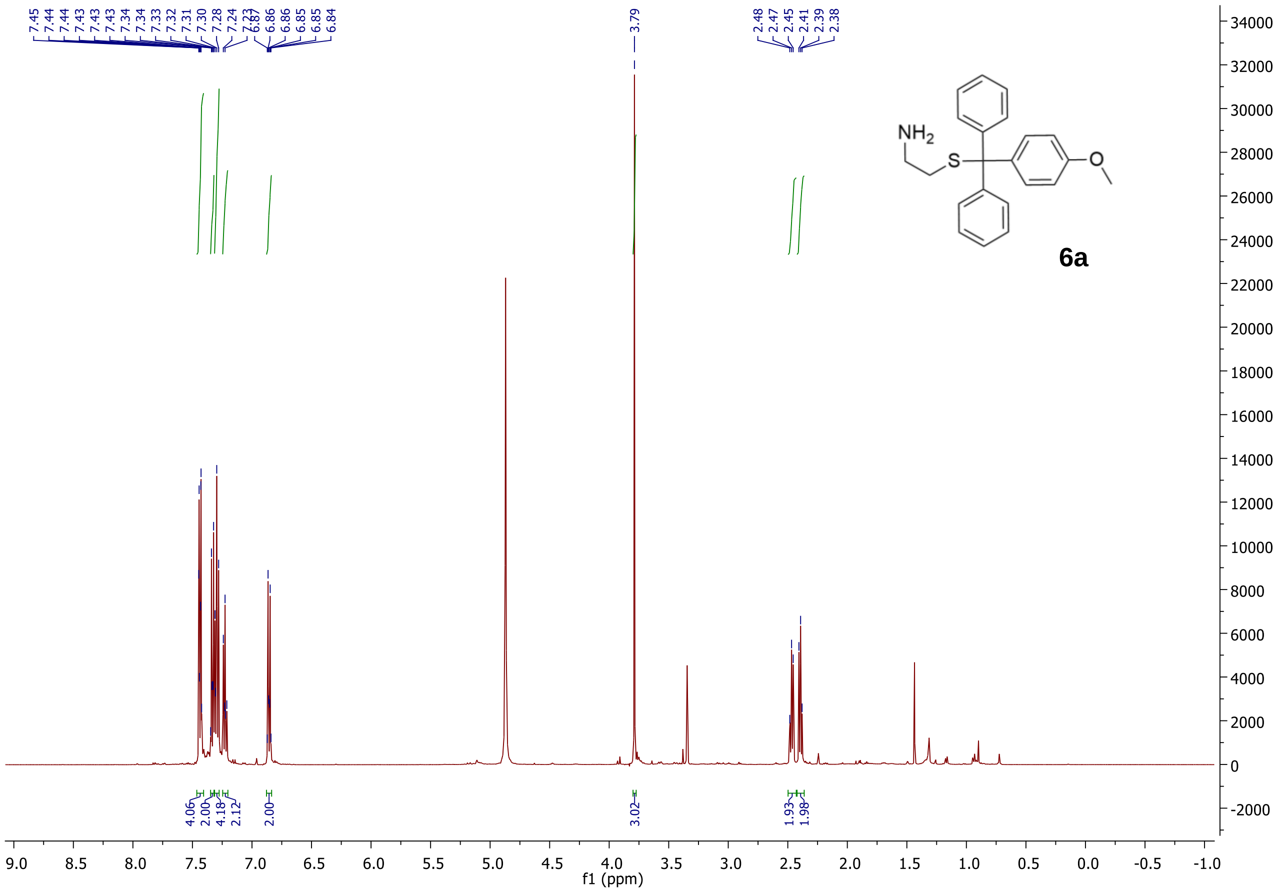

Fig. S 4 ^{1}H NMR spectrum (MeOD, 500 MHz) of S-methoxytrityl-cysteamine **6a**.

2. *On-resin formation of N-2-[thioethyl]glycine and further peptide precursor synthesis*

2.1. Manual synthesis

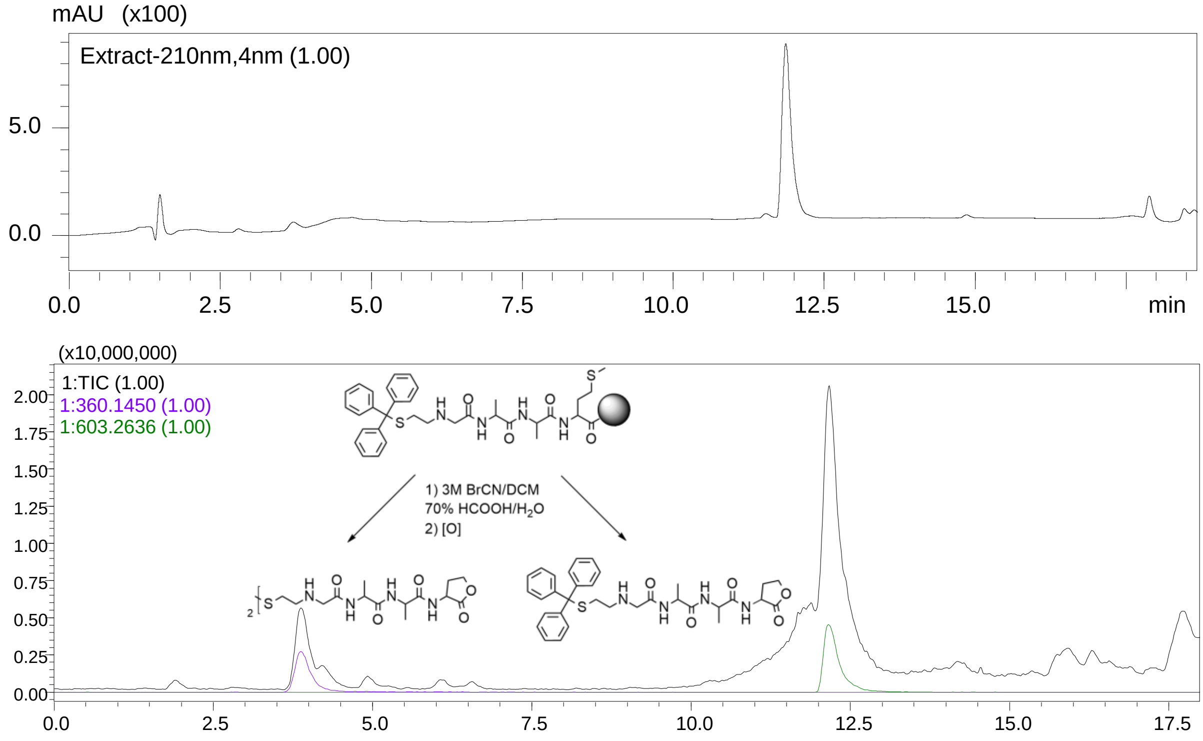

Fig. S 5 LC-UV-ESI-MS of crude AAM linker with incorporated *N*-2-[(trt)thioethyl]glycine after the cleavage by cyanogen bromide and spontaneous air oxidation (reaction scheme). Upon 70% HCOOH partial trityl deprotection is observed. LC: 1-60% B in 15min; Rt for *N*-2-[(trt)thioethyl]glycine-AA-HL (HL = homoserine lactone): 11.8-12 min, *m/z* calcd for $C_{33}H_{38}N_4O_5S$ $[M+H]^+$ 603.2635; Found 603.2639; Rt for *N*-[2-thioethylglycineAA-HL]$_2$ (disulfide linked homodimer): 3.6-3.8 min, *m/z* calcd for $C_{28}H_{46}N_8O_{10}S_2$ $[M+2H]^{2+}$ 360.1462*;* Found 360.1450; LC-UV-ESI-IT-TOF instrument.

2.2. Automated microwave-assisted synthesis

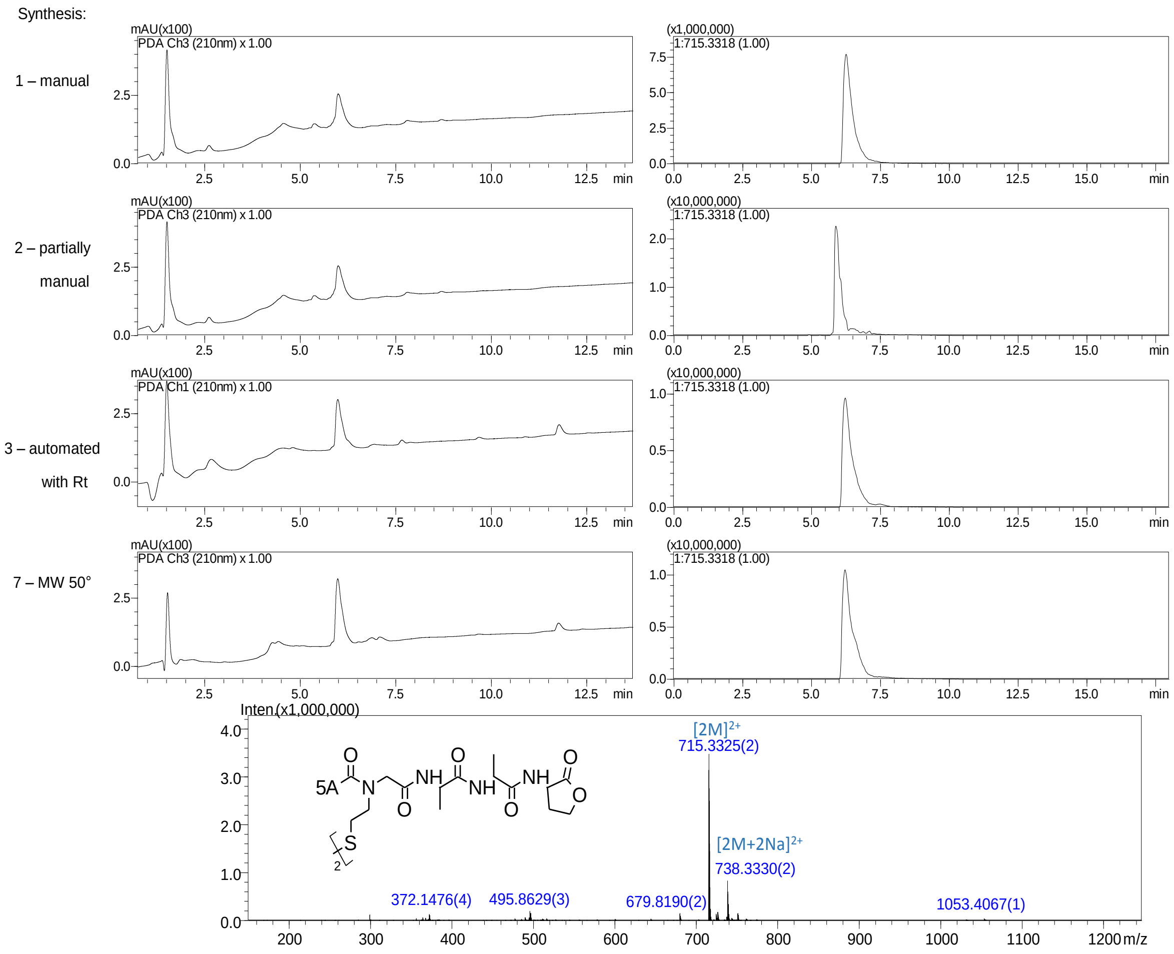

Fig. S 6 LC-UV at 210 nm (left) and LC-MS (right) chromatograms of crude AAAAA-*N*-2-[thioethyl]glycine-AA-HL peptide precursors (up) resulted in BrCN cleavage from TentaGel solid support; 1: fully manual synthesis, 2: partially automated synthesis of peptide precursor (AAAAA automated, *N*-2-[thioethyl]glycine-AAM manually), 3: an automated synthesis with room temperature incorporation of (trt)-cysteamine, 7: an automated synthesis with microwave incorporation of (trt)-cysteamine at 50° C, 2x1h (further description in the table below). LC: 1-60% B/A in 15'; rt 5.9-6.1 min; *m/z* calcd for $[2M]^{2+}$ 715.3318; found 715.3325. The lower part shows the ESI-MS at 6.0 min and the linker structure; LC-UV-ESI-IT-TOF.

Table S1. Tested conditions for manual, partially manual, and automated synthesis of peptide precursors: RT – room temperature, MW – microwave heating, US – ultrasound agitation

No	*Standard conditions*	*Conditions for the coupling with $ClCH_2COOH$*	*Conditions for nucleophilic substitution with (trt)cysteamine*	*Conditions for the coupling of the next FmAa*	*Final product*
1 – manual	3 eq FmAa/TBTU, 6 eq DIPEA, 20’ US<br>25% PIP/DMF 5’US	5 eq $ClCH_2COOH$/DIC, RT, 3x30’	6 eq (trt)cysteamine/DIPEA, RT, overnight (20h)	6 eq FmAa/TBTU, 12 eq DIPEA, 2x30’ US	+
2 – partially manual	5 eq FmAa/DIC/Oxyma, 5’ 75°<br>20% PIP/DMF, RT, 3’ and 10’, oscillating mixing	5 eq $ClCH_2COOH$/DIC, RT, 3x30’	6 eq (trt)cysteamine/DIPEA, Rt, overnight (20h)	5 eq FmAa/DIC/Oxyma<br>2 x 5’ 75°, MW	+
3 – automated with Rt	5eq FmAa/DIC/Oxyma, 5’ 75°, MW<br>20% PIP/DMF, RT, 3’ and 10’, oscillating mixing	5 eq $ClCH_2COOH$/DIC, RT, 3x30’, oscilating mixing	6 eq (trt)cysteamine, 2 eq DIPEA, RT, 10 h, oscillating mixing	5 eq FmAa/DIC/Oxyma<br>2 x 10’ 75°, MW	+
4 – MW3x10’	5eq FmAa/DIC/Oxyma, 5’ 75°, MW<br>20% PIP/DMF, RT, 3’ and 10’, oscillating mixing	7 eq $ClCH_2COOH$/DIC<br>3x10’ 75°, MW	10 eq (trt)cysteamine, 3 eq DIPEA, 2h 75°, MW	5 eq FmAa/DIC/Oxyma<br>2 x 10’ 75°, MW	-
5 – MW2x1h	5eq FmAa/DIC/Oxyma, 5’ 75°, MW<br>20% PIP/DMF, RT, 3’ and 10’, oscillating mixing	7 eq $ClCH_2COOH$/DIC<br>RT, 3x30’, oscilating mixing	10 eq (trt)cysteamine, 2x1h, 75°	7,5 eq FmAa/DIC/Oxyma<br>2 x 10’ 75°, MW	+
6 – MW2x0.5h	5eq FmAa/DIC/Oxyma, 5’ 75°, MW<br>20% PIP/DMF, RT, 3’ and 10’, oscillating mixing	7 eq $ClCH_2COOH$/DIC<br>RT, 3x30’, oscilating mixing	10 eq (trt)cysteamine, 2x0,5h, 75°, MW	7,5 eq FmAa/DIC/Oxyma<br>2 x 10’ 75°, MW	+
7 – MW50°	5eq FmAa/DIC/Oxyma, 5’ 75°, MW<br>20% PIP/DMF, RT, 3’ and 10’, oscillating mixing	7 eq $ClCH_2COOH$/DIC<br>RT, 3x30’, oscilating mixing	10 eq (trt)cysteamine, 2x1h, 50°, MW	7,5 eq FmAa/DIC/Oxyma<br>2 x 10’ 75°, MW	+
8 – MW2x15’	5eq FmAa/DIC/Oxyma, 5’ 75°, MW<br>20% PIP/DMF, RT, 3’ and 10’, oscillating mixing	7 eq $ClCH_2COOH$/DIC<br>RT, 3x30’, oscilating mixing	10 eq (trt)cysteamine, 2x15’ 75°, MW	9 eq FmAa/Oxyma/DIC, 2 x 10’ 75°, MW	-

3. *Fmoc substitution level determination*

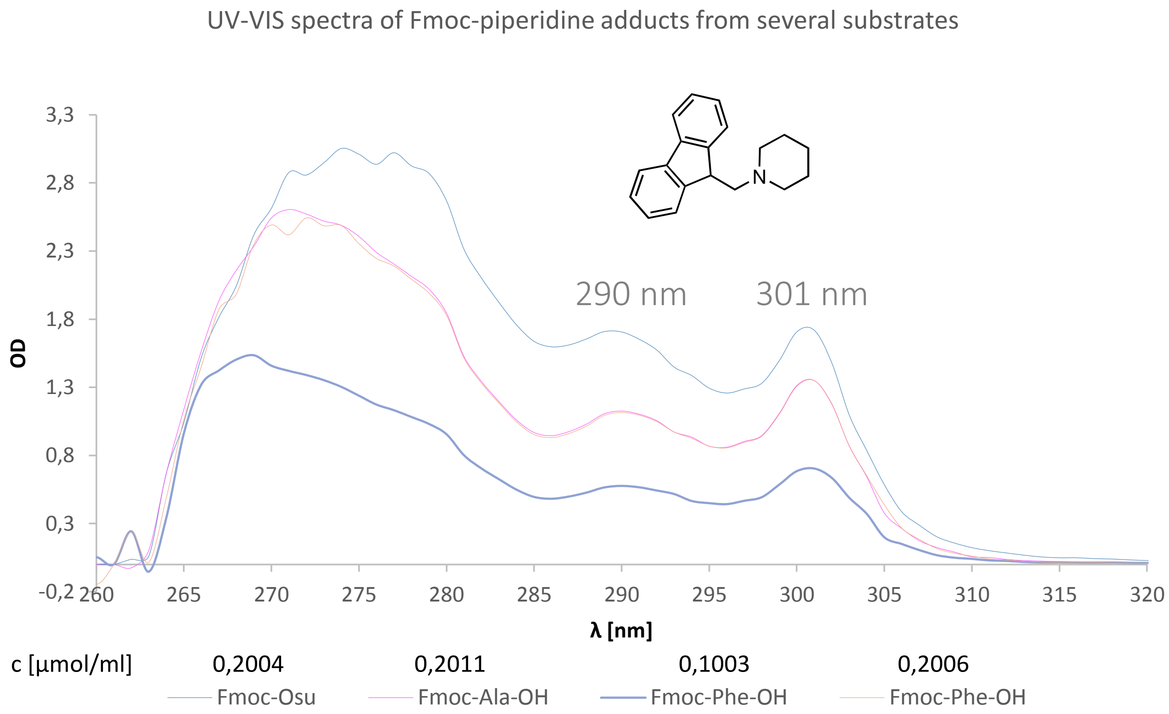

Fig. S 7 UV-VIS spectra of the dibenzofulvene-piperidine adduct (which structure is shown) after incubation for 20’ in 20% PIP/DMF of several substrates: Fmoc-Osu – succinimide ester, Fmoc-alanine, and Fmoc-phenylalanine.

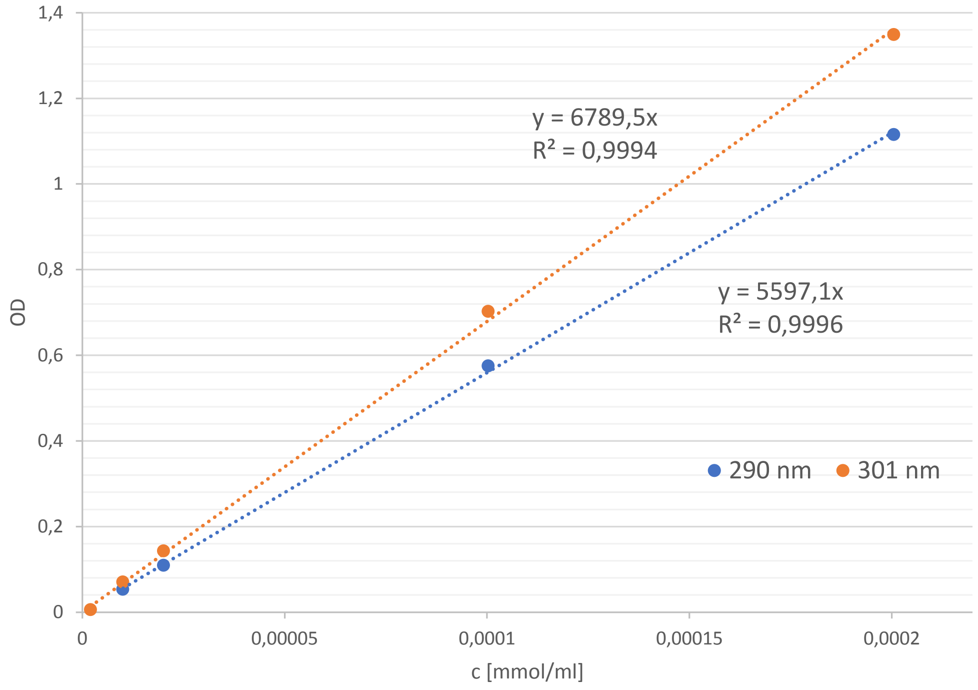

Fig. S 8 Calibration curves for the set of Fmoc-Phe-OH concentrations after incubation with 20% PIP/DMF calculated for two maxima of absorption: 290 and 301 nm.

4. *Study on different linkers and their impact on final cyclization of CAKPGG*

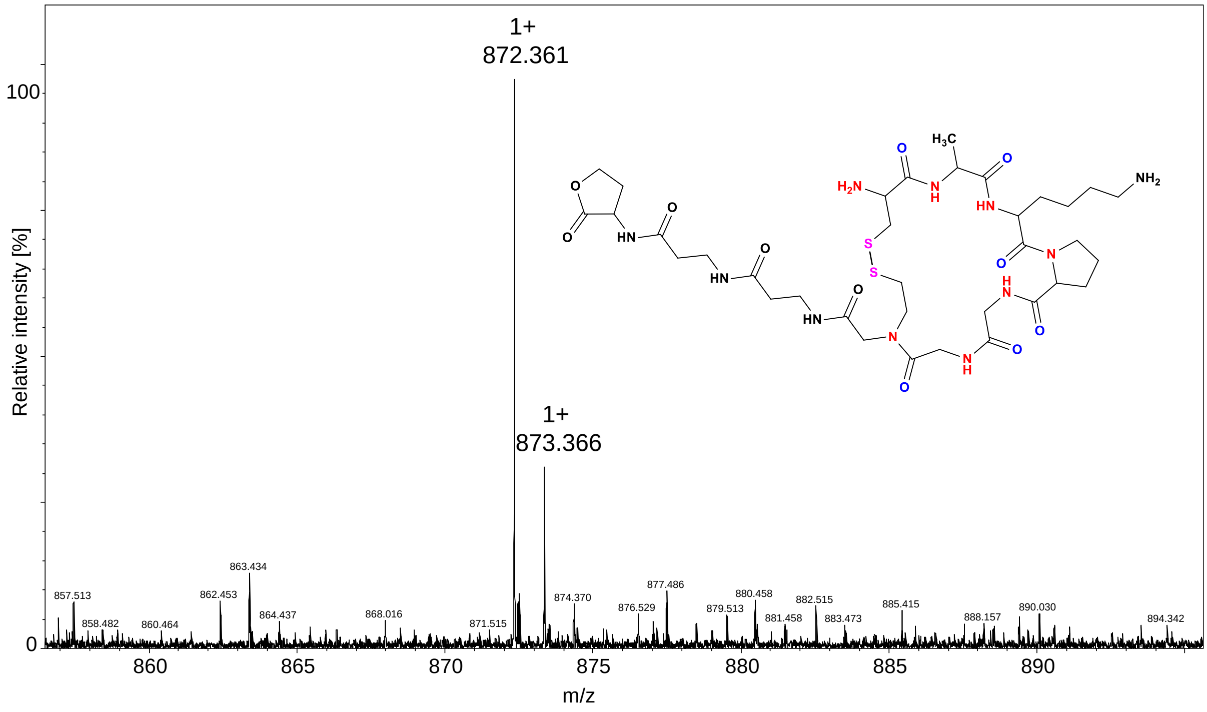

Fig. S 9 ESI-MS spectrum acquired for linear precursor (with linker 1) of cyclo-CAKPGG-N-2-[thioethyl]glycine-βAβA-HL after its cleavage from the resin by cyanogen bromide.

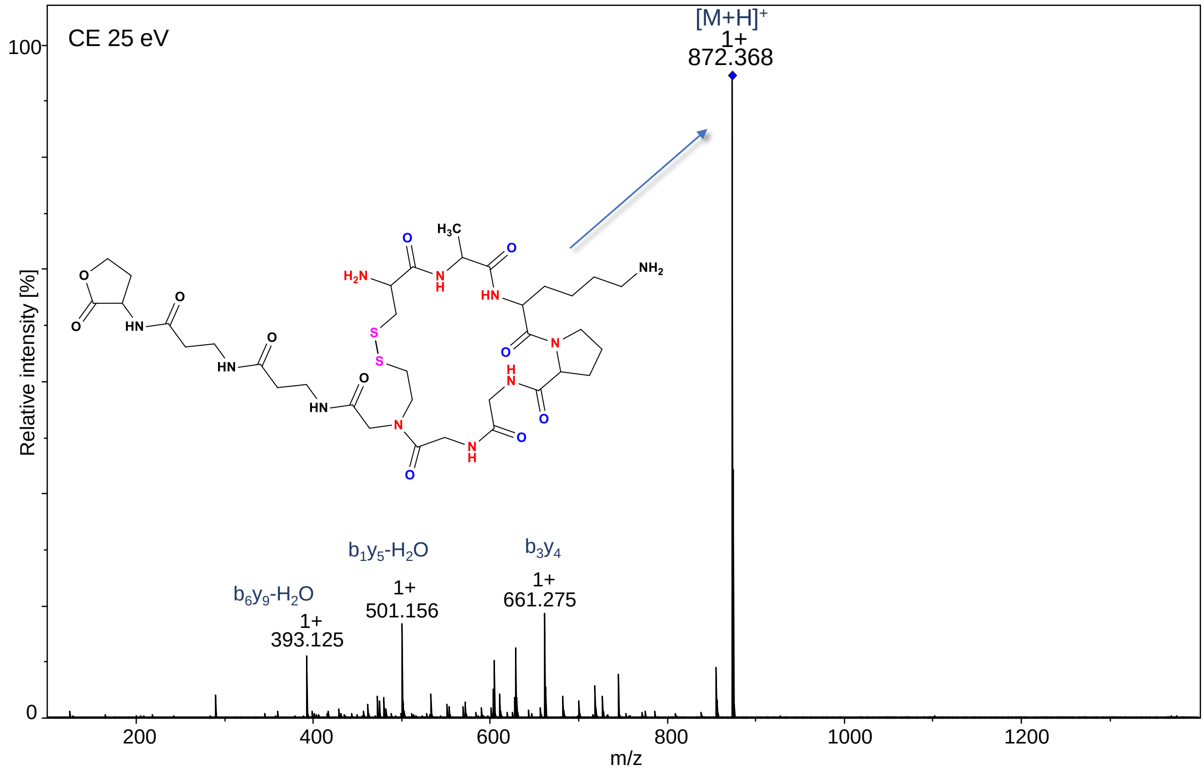

Fig. S 10 ESI-MS/MS (p.i. 872.368 *m/z*) spectra acquired for linear precursor (with linker 1) of CAKPGG-*N*-2-[thioethyl]glycine-βAβA-HL after its cleavage from the resin by cyanogen bromide (CE 25eV).

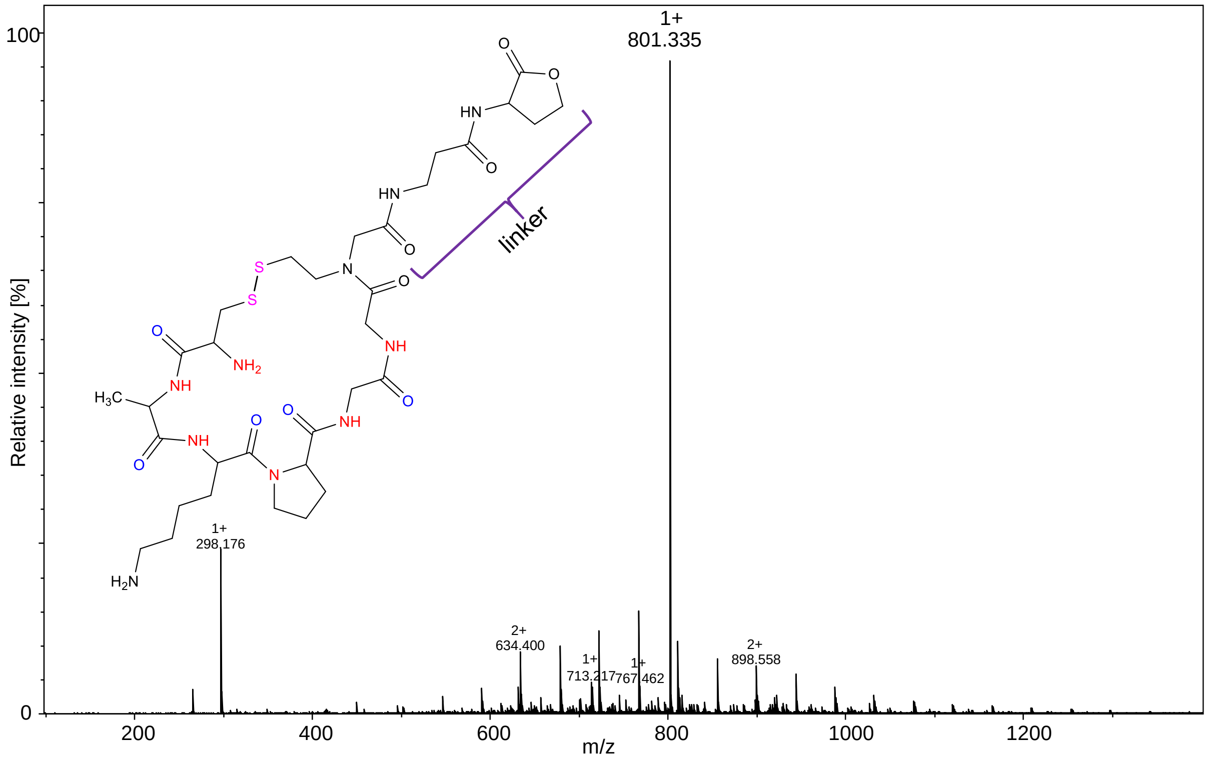

Fig. S 11 ESI-MS spectrum acquired for linear precursor (with linker 2) of CAKPGG-*N*-2-[thioethyl]glycine-βA-HL after its cleavage from the resin by cyanogen bromide.

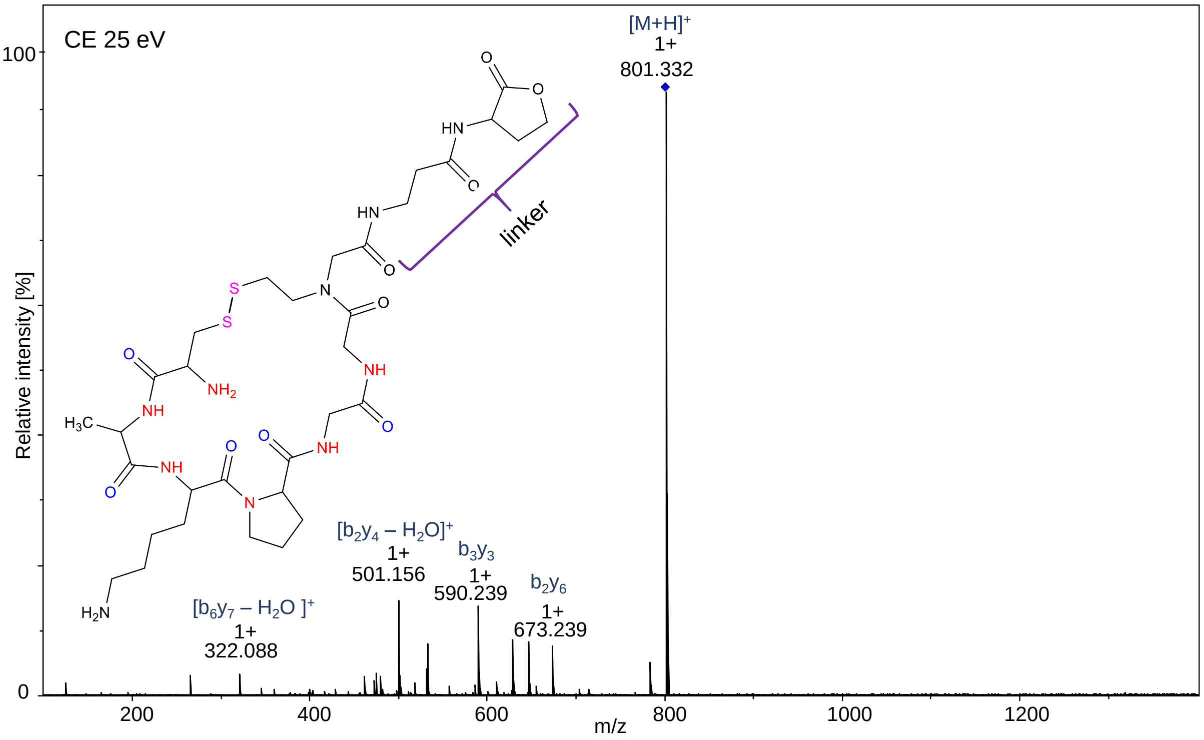

Fig. S 12 ESI-MS/MS (p. i. 801.332 *m/z*) spectrum acquired for linear precursor (with linker 2) of CAKPGG-*N*-2-[thioethyl]glycine-βA-HL after its cleavage from the resin by cyanogen bromide (CE 25eV).

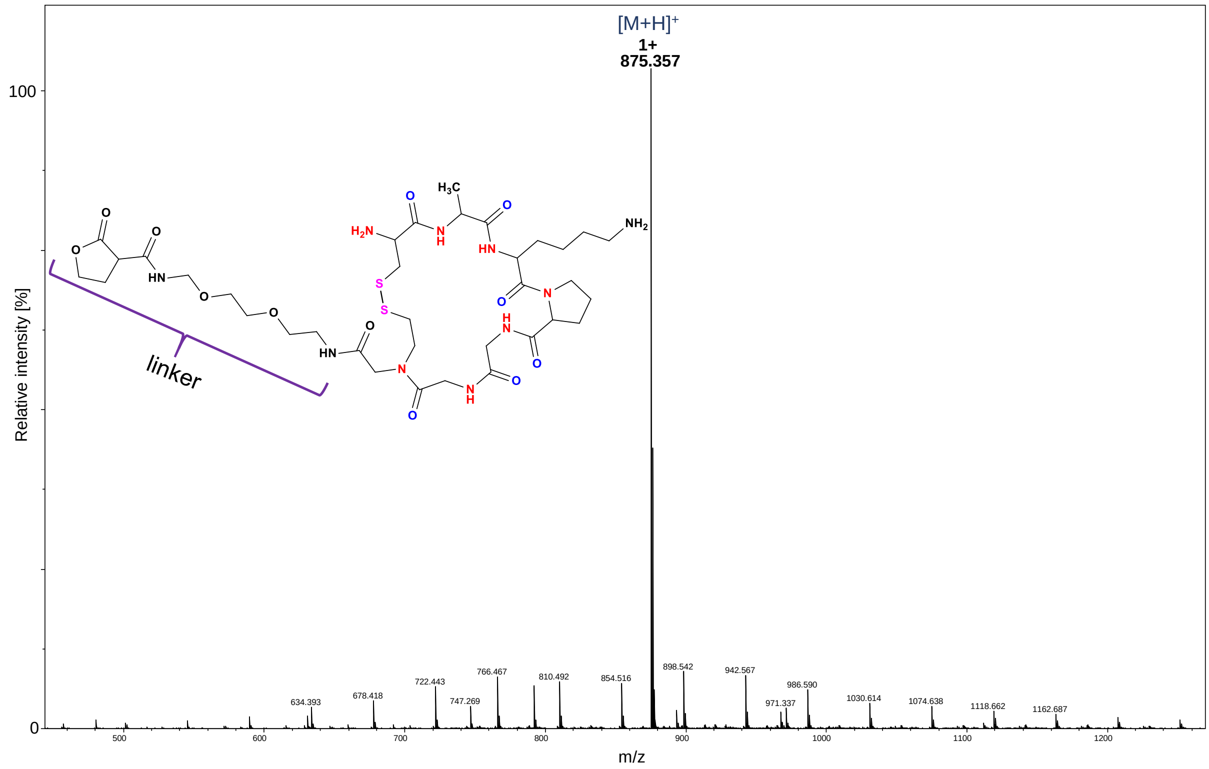

Fig. S 13 ESI-MS spectrum acquired for linear precursor (with linker 4) of CAKPGG-*N*-2-[thioethyl]glycine-PEG-HL after its cleavage from the resin by cyanogen bromide.

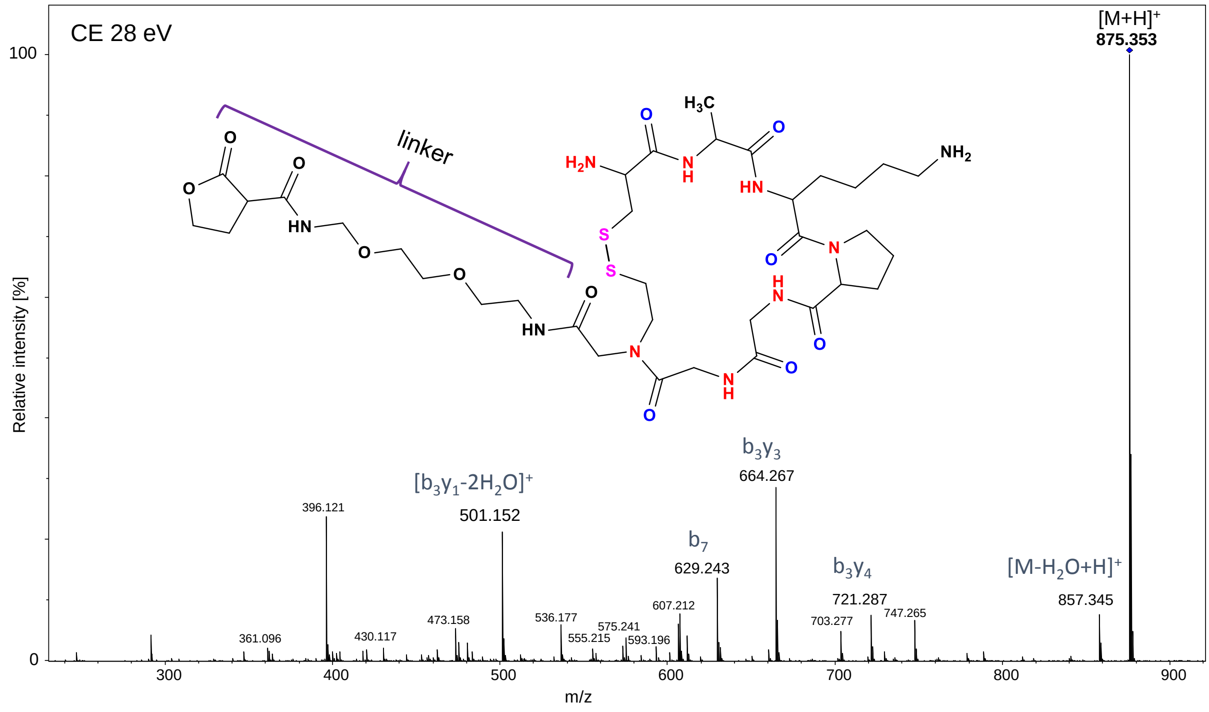

Fig. S 14 ESI-MS/MS (p. i. 875.357 *m/z*) spectrum acquired for linear precursor (with linker 4) of CAKPGG-*N*-2-[thioethyl]glycine-PEG-HL after its cleavage from the resin by cyanogen bromide (CE 25eV).

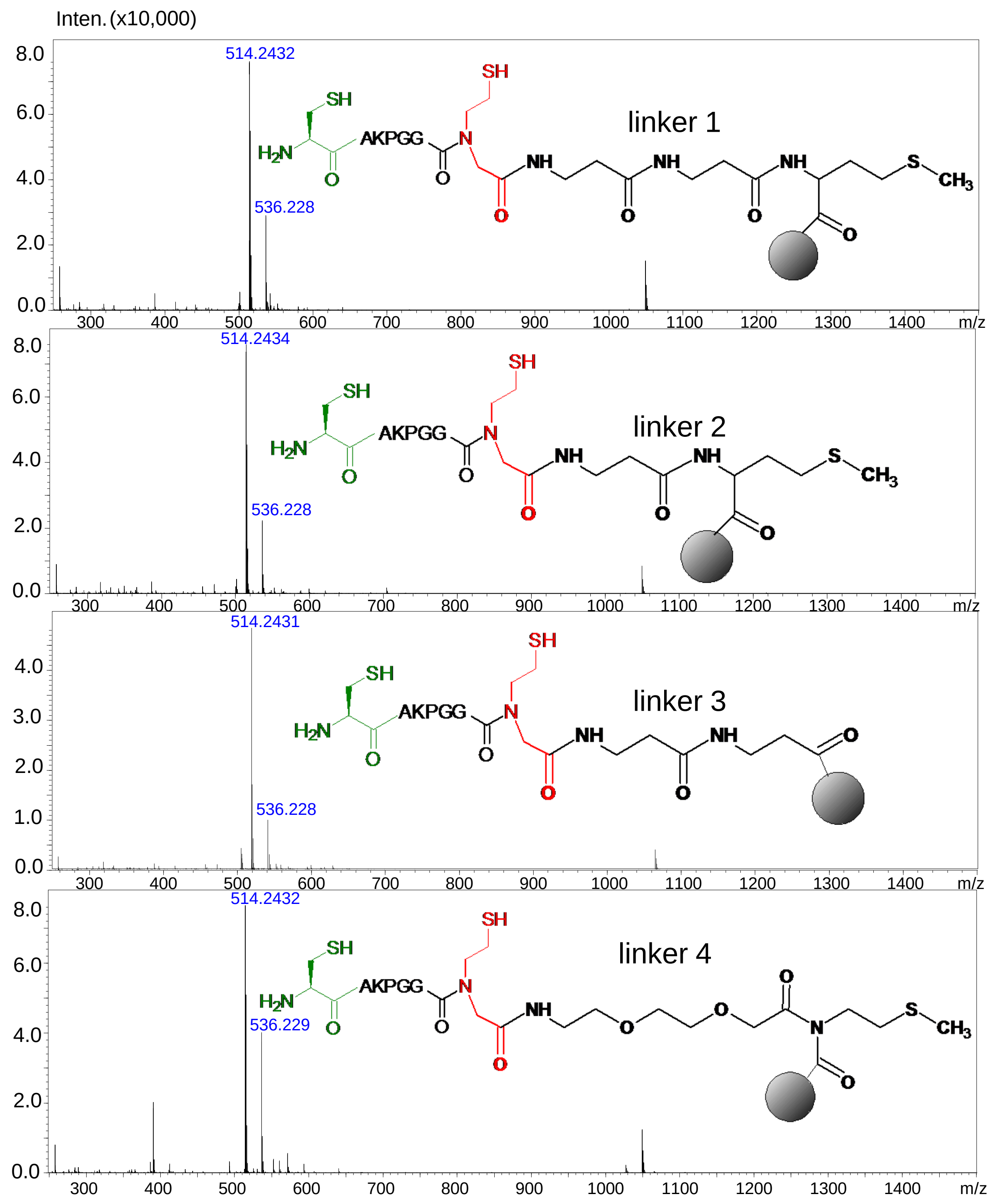

Fig. S 15 Comparison of LC-ESI-MS spectra acquired for model peptides cyclo-CAKPGG (HRMS: ESI-qTOF; *m/z:* $[M+H]^+$ Calcd for $C_{21}H_{36}N_7O_6S$ 514.2442; Found 514.2432 ±0.0002) after their liberation from solid support *via* tandem acyl shift followed by native chemical ligation using four different linkers. For the comparison, the same amount of resin was used. Samples for analysis were prepared using an equal volume of water, and then the same injection volumes during the LCMS analysis were applied.

5. *Cleavage of peptide thioesters without N-terminal cysteine*

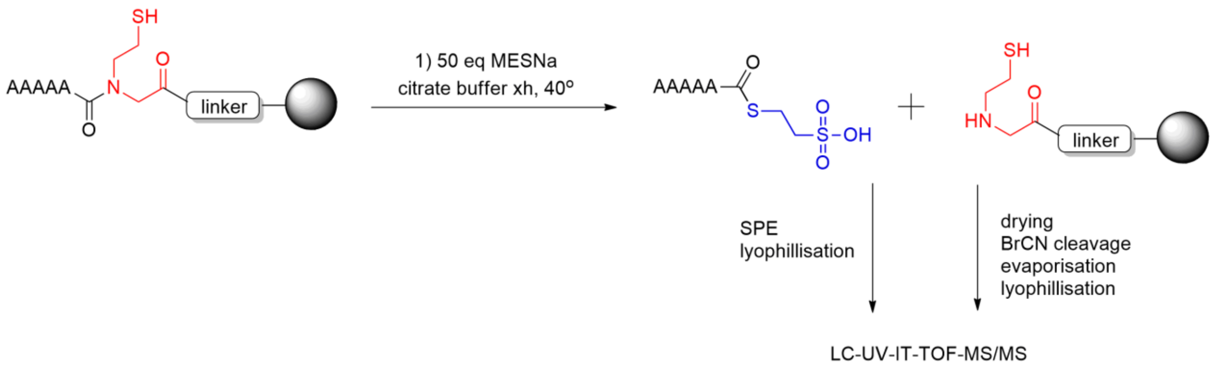

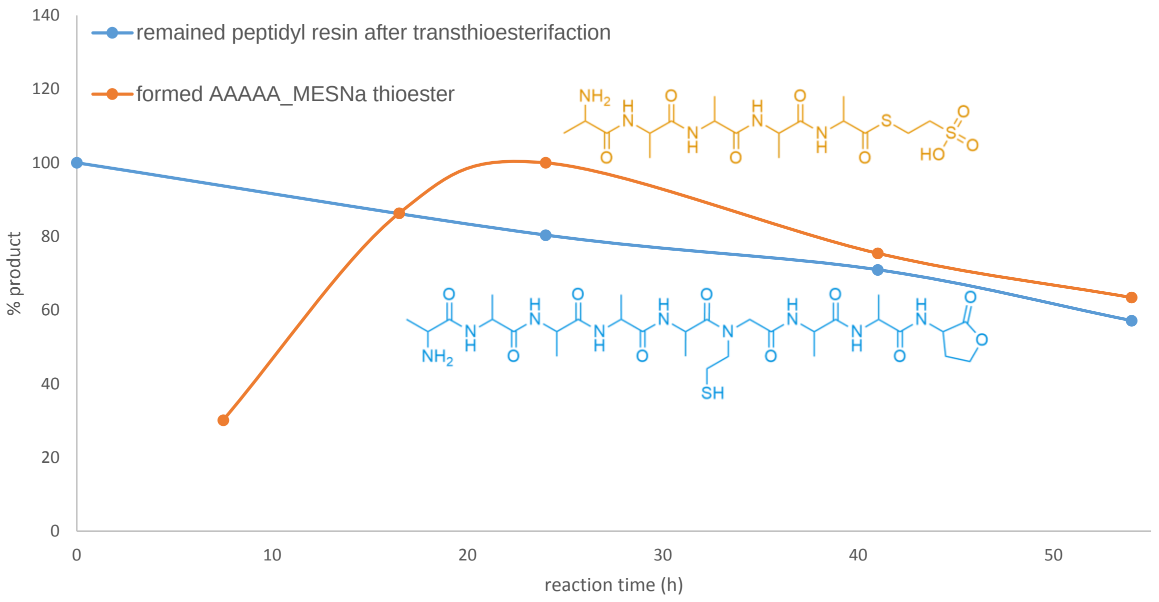

Fig. S 16 Graph of product yields in time for the formed H-AAAAA-MESNa thioester (orange) and the remained on the resin substrate (blue) determined from LC-UV-MS data on ESI-IT-TOF instrument.

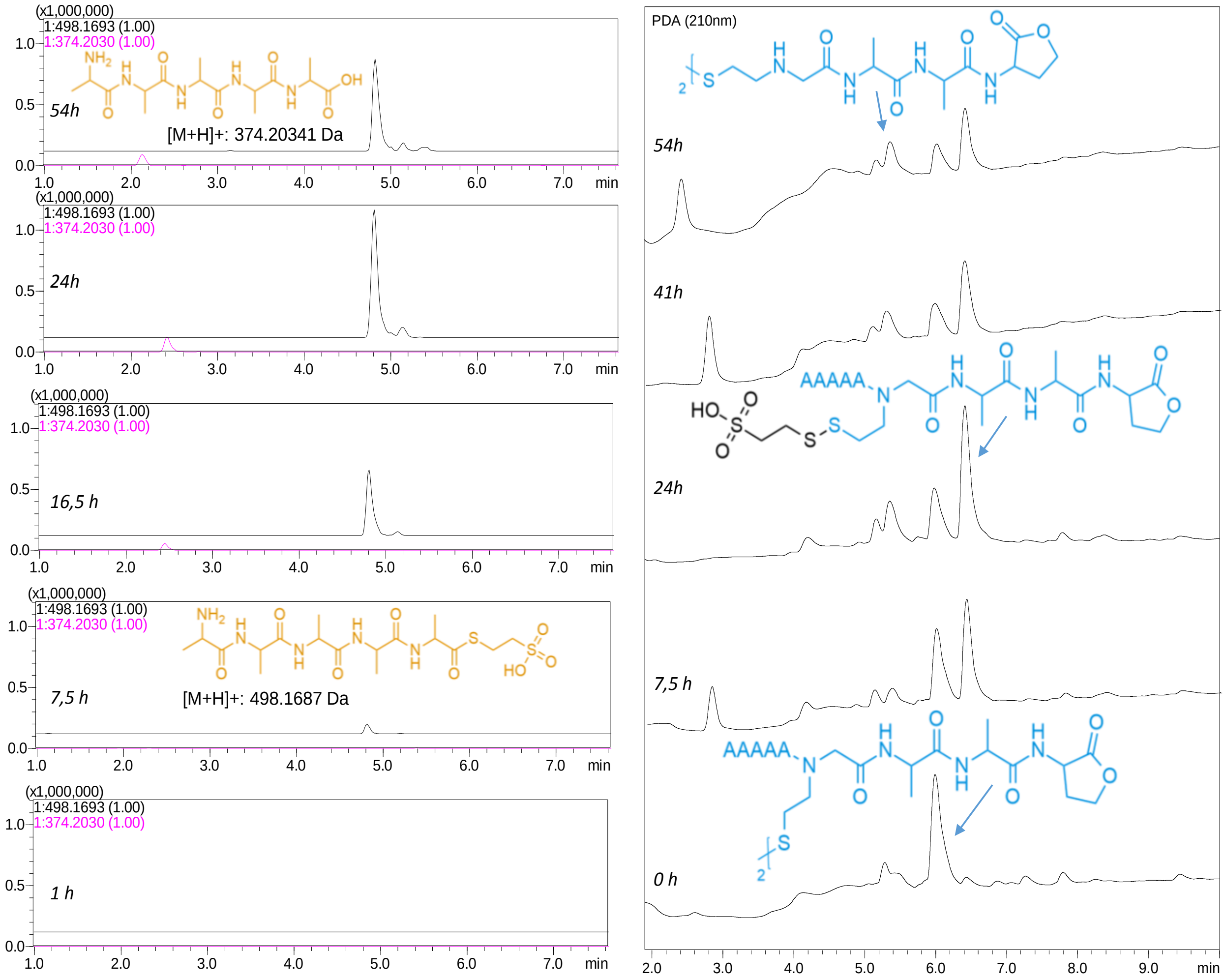

Fig. S 17 LC-MS (left): XIC of the formed H-AAAAA-MESNa thioester after the time (*m/z* calcd. 498.1687, found 498.1693) and hydrolyzed H-AAAAA-OH (*m/z* calc. 374.2034, found 374.2030) and LC-UV at 210 nm (right) of the cleaved by BrCN residue after transthioesterification: Rt of the unreacted substrate: 5.9-6.1 min; side product linked with MESNa by disulfide bridge: 6.35-6.5 min; transthioesterification product – *N*-2-[thioethyl]glycine-AA-HL: 5.4-5.45 min. LC: 1-60% B/A in 15 min, ESI-IT-TOF instrument.

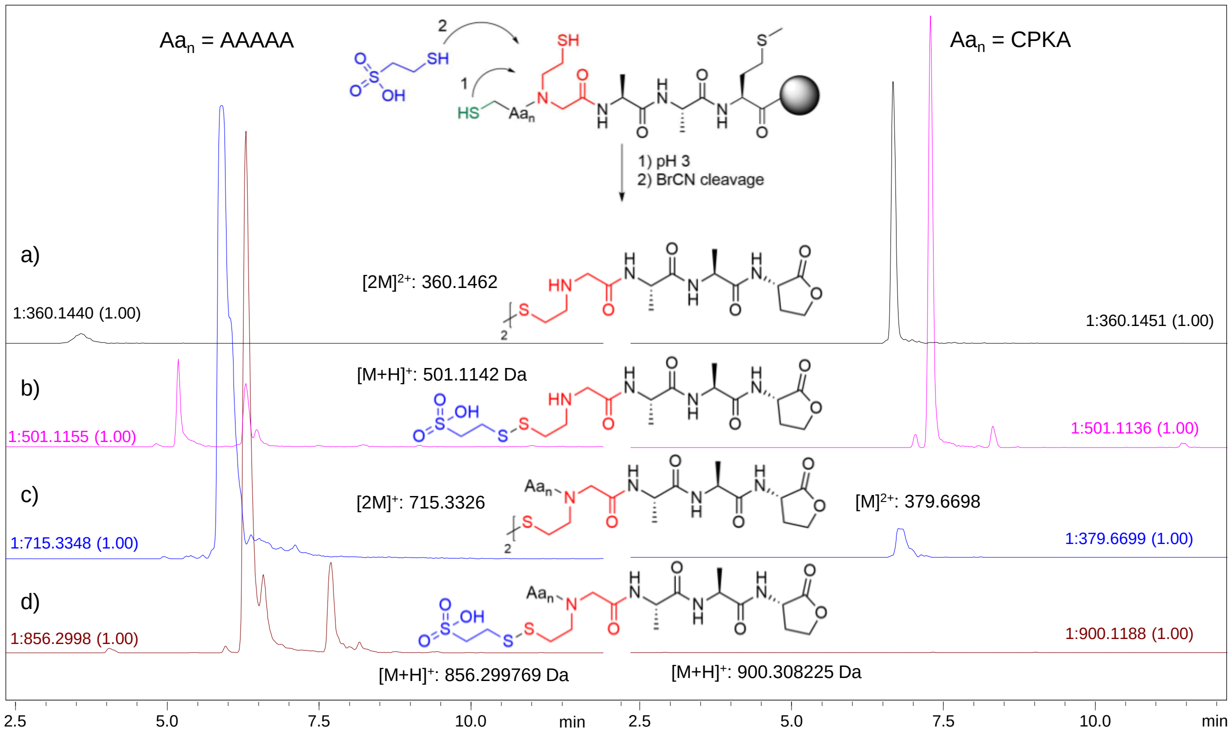

Fig. S 18 Comparison of the transthioesterification of two different peptide sequences in the same conditions (50 eq of MESNa in citrate buffer for 24h in 40°C) by LC-MS: left chromatogram: AAAAA sequence and right chromatogram: CPKA sequence on precursor: *N*-2-[thioethyl]glycine-AAM-TentaGel NH_2. After incubation, the remained peptidyl resin was cleaved by cyanogen bromide to determine the completeness of transthioesterification. Chromatograms a correspond to the XIC of the oxidized *N*-2-[thioethyl]glycine-AA-HL (homoserine lactone), which is the transthioesterification product. Besides, there is also formed a product linked with MESNa by disulfide bridge – b. In case of CPKA, they give the most abundant signal showing that the cleavage procedure was quantitative. However for the AAAAA sequence, the transthioesterification was not satisfactory, and the substrate Aa_n-*N*-2-[thioethyl]glycine-AA-HL signal was the most abundant, which corresponds to the chromatogram c. Unlikely MESNa formed faster a disulfide bridge with *N*-2-[thioethyl]glycine – chromatogram d – and thus completely blocked the substrate. This product is not observed for CPKA, which means that in case of *N*-terminal cysteine sequences, the NCL is faster than disulfide bridge formation with MESNa. Product structures and *m/z* values are shown as well.

6. Analytical data for the synthesized peptides

*6.1. CycloCFGPKA **1***

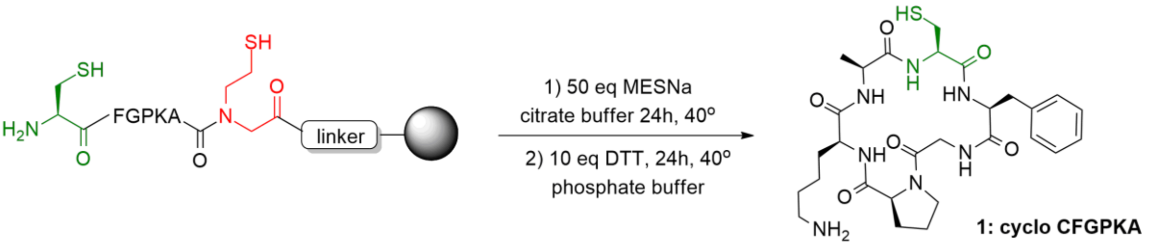

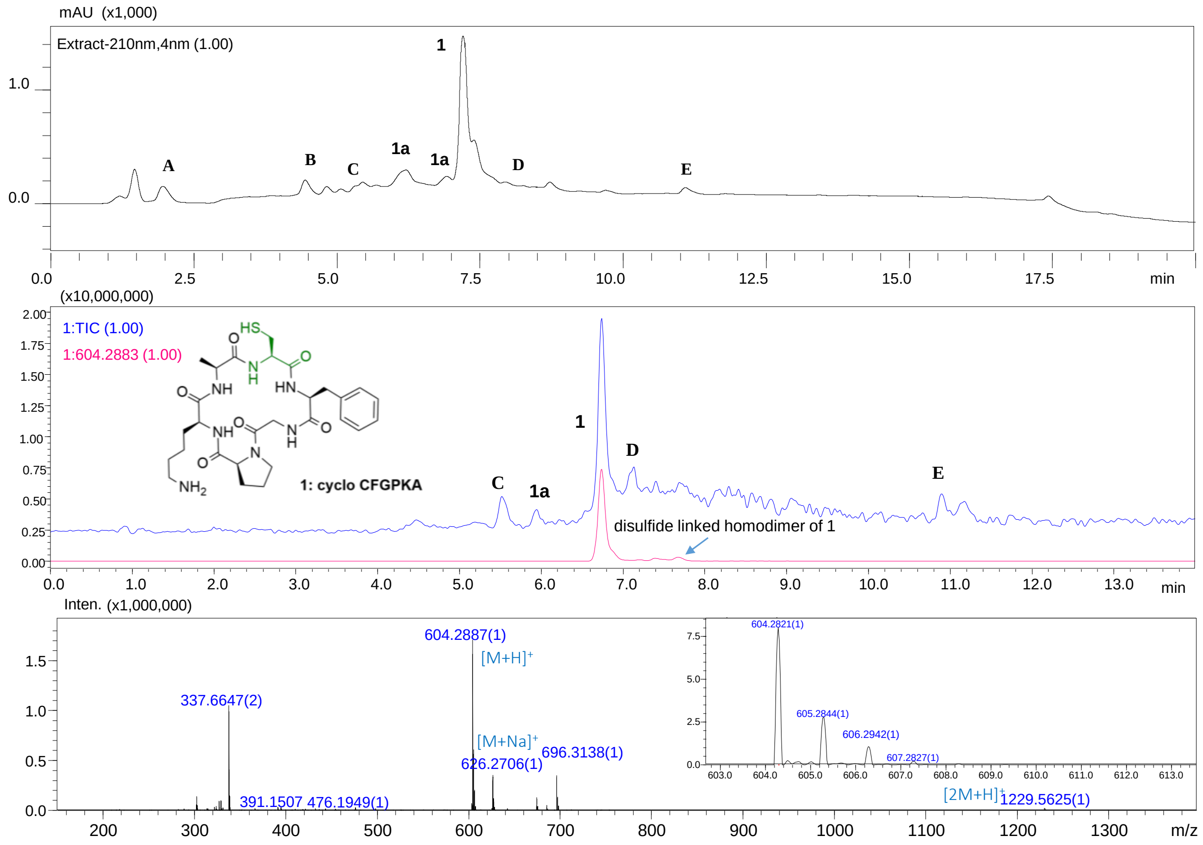

Nr	rt (min)	m/z Found	m/z Calcd	Charge state	Identification
A	2.0	594.2644	-	1+	not identified (n. i.)
B	4.5	626.2620	626.2731	1+	sodium adduct of 1: [M+Na]+
C	5.3	643.2646	-	1+	n. i.
1a	6.0	746.2526	746.2670	1+	MESNa thioester of linear 1
1	7.2	604.2883	604.2912	1+	1 - cycloCFGPKA
D	7.6	696.3021<br>744.2380	-<br>744.2513	1+<br>1+	n. i.<br>disulide-linked heterodimer of 1 with MESNa
E	11.1	700.2626	-	1+	n. i.

Fig. S 19 LC of desalted crude **1**, from the top: UV chromatogram at 210 nm with marked signals, MS chromatogram with TIC (total ion chromatogram) and XIC (extracted ion chromatogram) of **1** ($[M+H]^+$ Calcd = 604.2911; Found 604.2887), MS of the crude mixture with the zoomed-in isotopic pattern and table with marked signals; instrument ESI-IT-TOF, gradient: 5-65% B/A in 15 min.

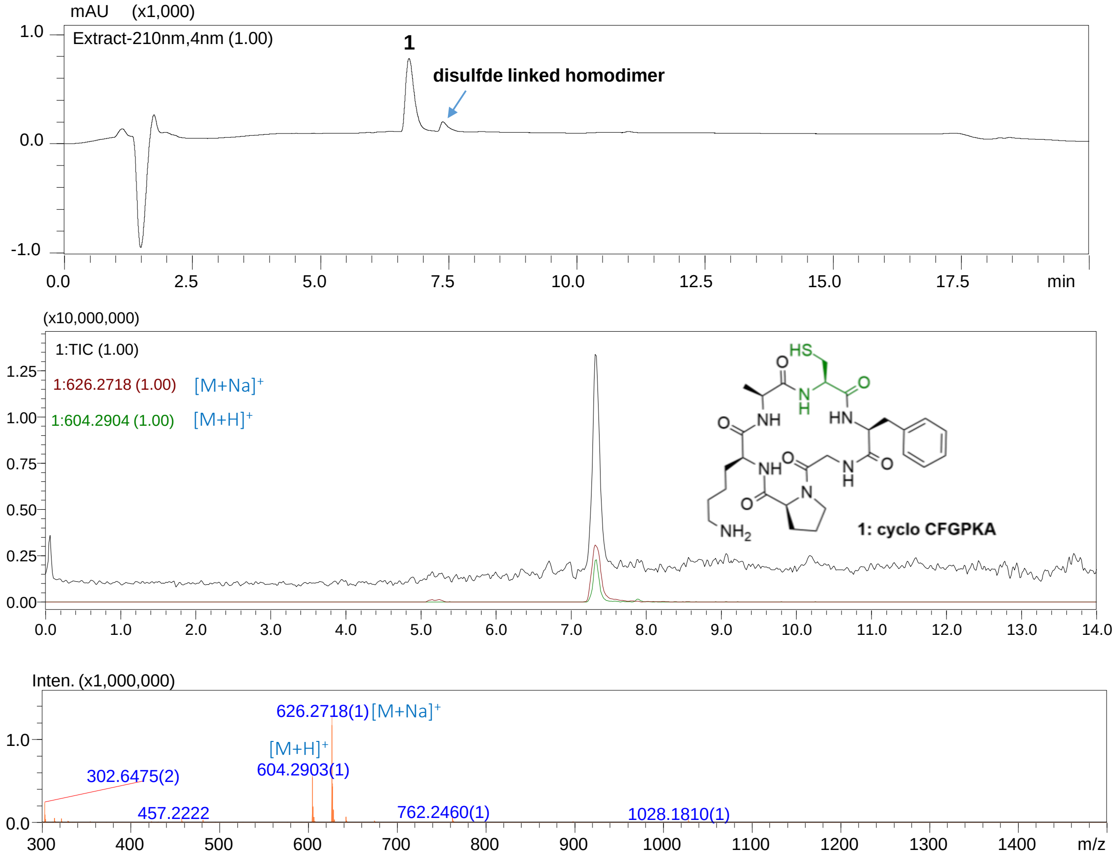

Fig. S 20 LC-UV-MS of purified **1**: upper part – UV chromatogram at 210 nm, middle part – MS chromatogram with TIC and XIC of **1** $[M+H]^+$ (604.2904) and its sodium adduct $[M+Na]^+$ (626.2718); lower part – MS spectrum at 7.2-7.5 min; instrument ESI-IT-TOF, gradient: 5-65% B/A in 15 min.

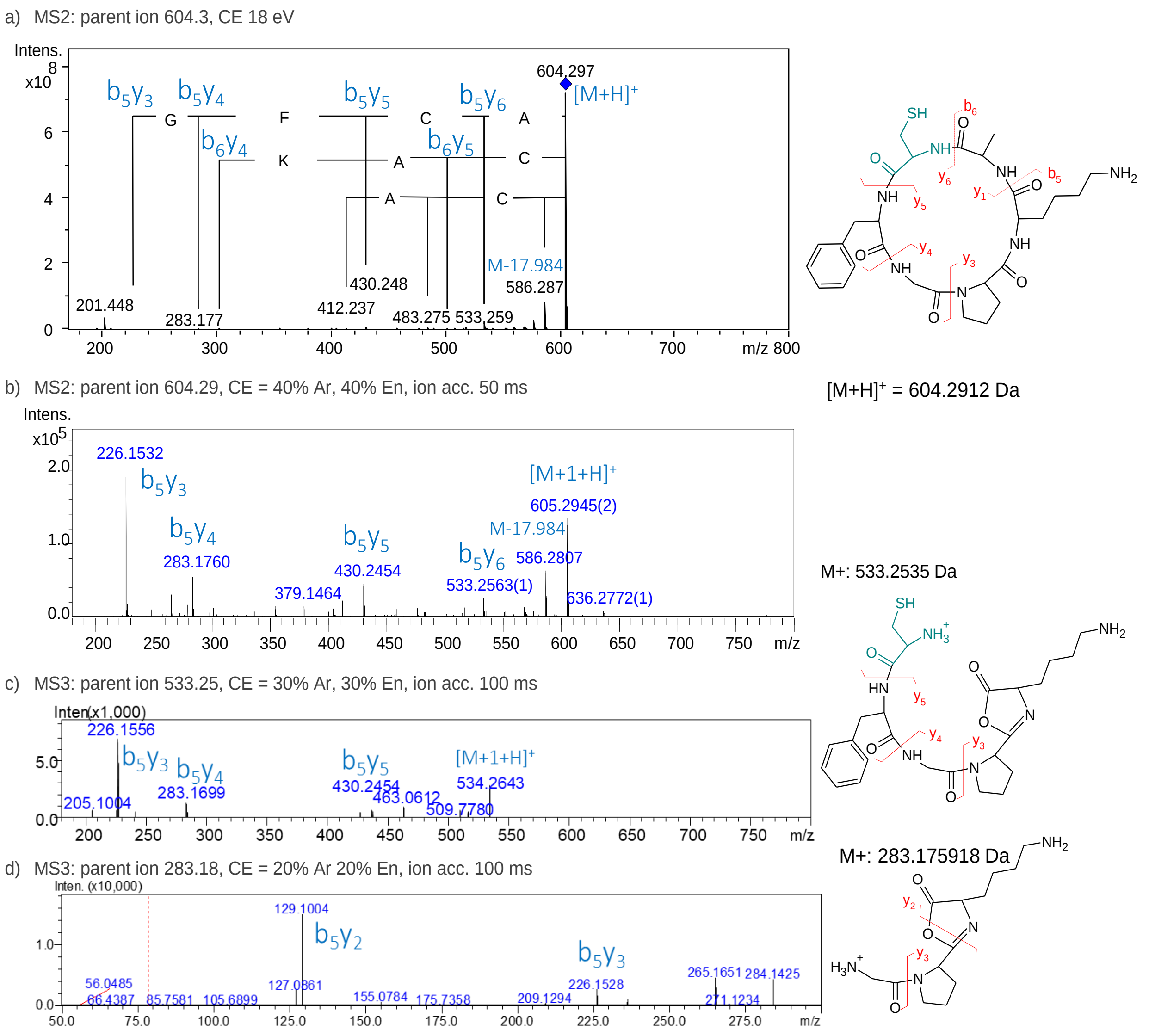

Fig. S 21 MS^n analysis of cyclo CFGPKA **1**: a) MS/MS recorded on ESI-FT-ICR with CID fragmentation at 18eV with the marked neutral losses and fragment ions. The presented structure shows the dissociated bonds and the dotted line shows the ring openings that lead to two fragmentation pathways; b) MS^2 recorded on ESI-IT-TOF instrument with CID fragmentation at 40% of maximum Energy (En) and 40% of maximum Argon beam (Ar); c) MS^3 of the b_5y_6 (533.25) from b); d) MS^3 of the b_5y_4 (283.17) from b).

1 mg of peptide **1** was dissolved in 1 ml of deionized water and incubated for several days at rt and measured on analytical HPLC (Thermo). The fractions were collected and identified by ESI-FT-ICR-MS.

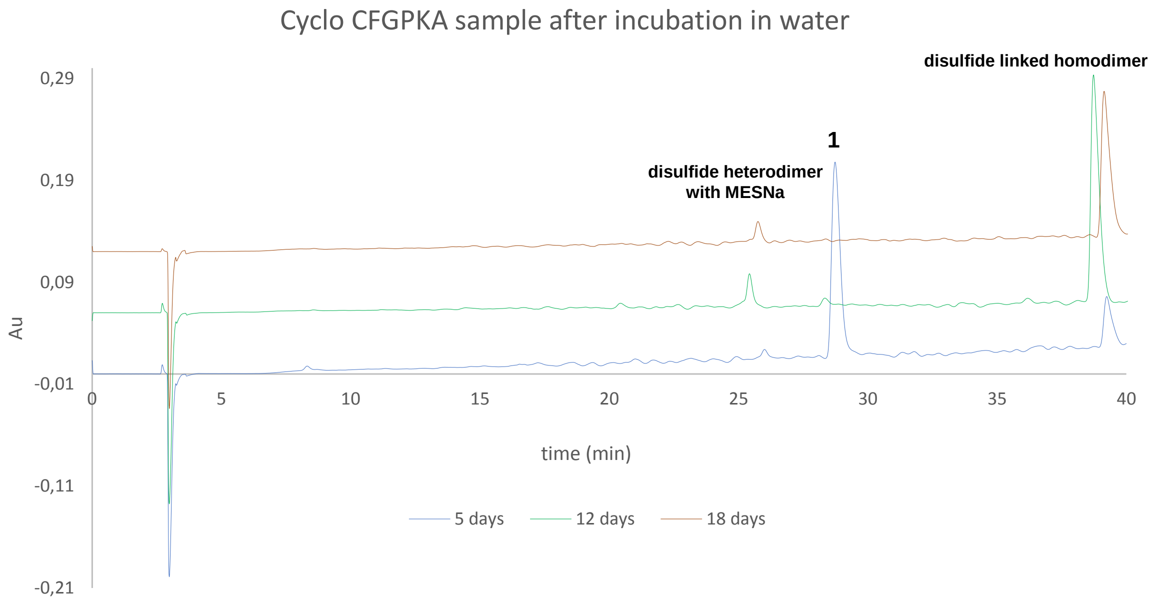

Fig. S 22 HPLC of **1** after incubation in water for 5, 12, and 18 days. A dimer with intermolecular S-S bridge was formed and its rt is 38,5-39,5 min; instrument Thermo, gradient: 0-80% B/A in 40 minutes.

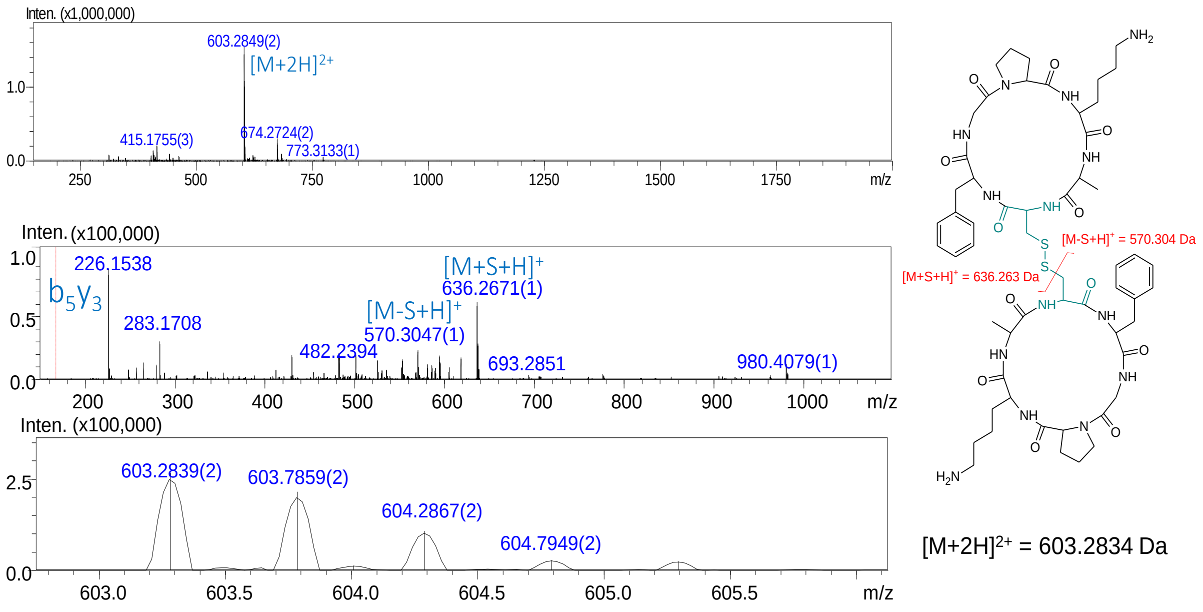

Fig. S 23 MS spectrum of oxidized, disulfide-linked **1** which structure is shown and its MS/MS (p.i. 603.28) recorded on ESI-IT-TOF instrument with CID at 40% of maximum Energy and 40% of maximum Argon beam and $[M+S+H]^+$ and $[M-S+H]^+$ fragment ions marked. The fragmentation pattern was characteristic for cyclic peptides and the fragmentation of disulfide bridge proceeds through C-S or S-S bond following literature[1,2].

---

[1] Mormann M.; Eble J.; Schwöppe C.; Mesters R. M.; Berdel W. E.; Peter-Katalinić J.; Pohlentz G. Fragmentation of intra-peptide and inter-peptide disulfide bonds of proteolytic peptides by nanoESI collision-induced dissociation. Anal Bioanal Chem 2008, 392, 831–838

[2] Goyder M. S.; Rebeaud F.; Pfeifer M. E.; Kálmán F. Strategies in mass spectrometry for the assignment of Cys-Cys disulfide connectivities in pro-teins. Expert Rev Proteomics, 2013, 10, 489-501

*6.2. 2-mercaptoethanesulfonate thioester of CFGPKA **1a***

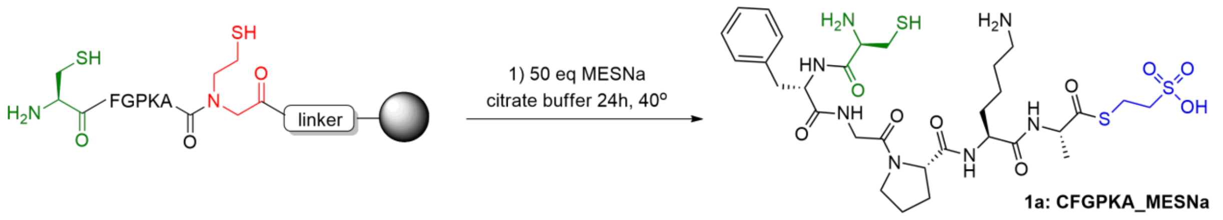

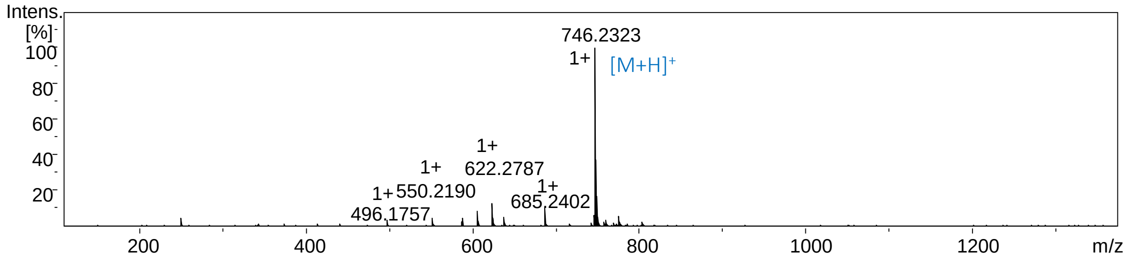

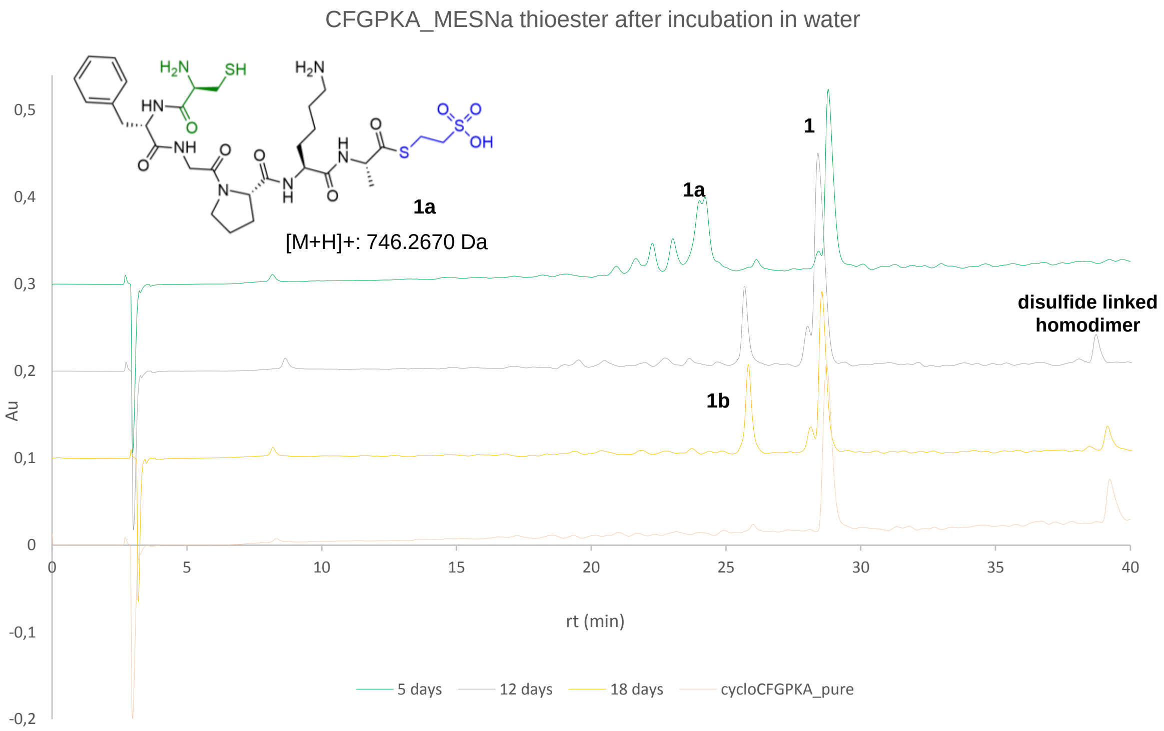

Fig. S 24 MS spectrum (FT-ICR) of **1a** (MESNa thioester of CFGPKA), which structure is shown, and HPLC chromatograms (Thermo) of the thioester incubated in water for 5, 12, and 18 days at rt. HPLC chromatogram of cyclo CFGPKA is shown for comparison. The thioester firstly undergoes as well to the native chemical ligation forming **1** (rt 28.5-29.0 min.) and to the hydrolysis giving the linear peptide **1b** (rt 25.5-26.0 min). Formed **1** undergoes the oxidation forming disulfide-linked homodimer (38.5-39.0 min); gradient: 0-80% B/A in 40 min.

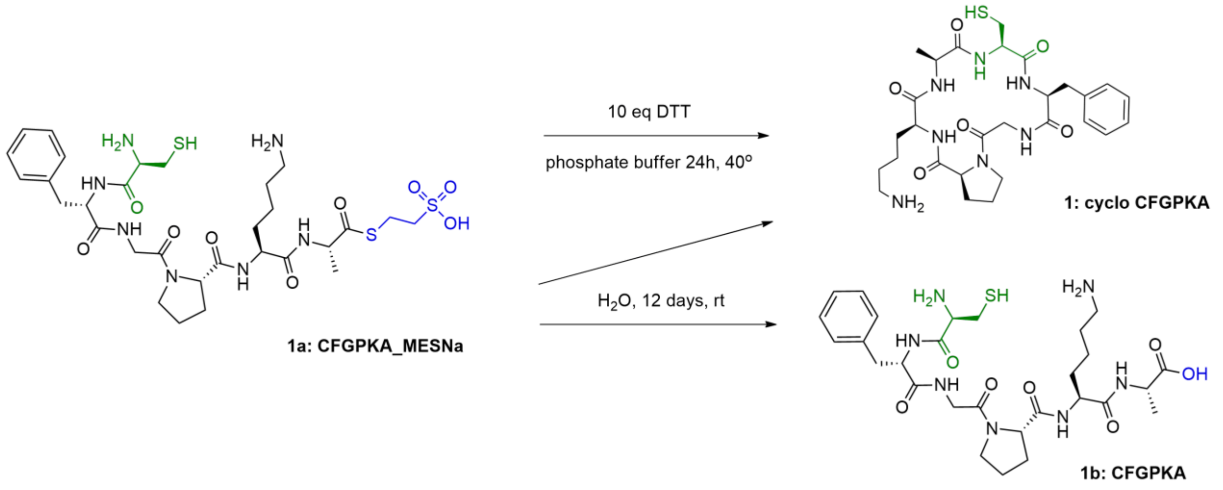

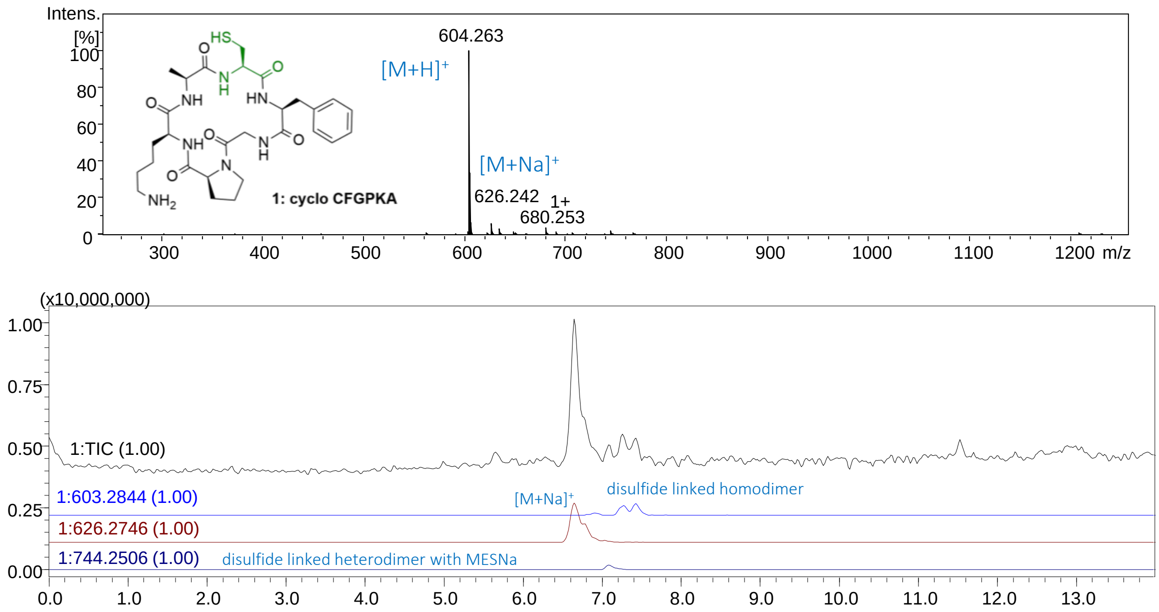

Fig. S 25 MS spectrum (ESI-FT-ICR, upper) of the desalted reaction mixture after the treatment of **1a** with 10 eq of DTT in phosphate buffer in 40° and its MS chromatogram (ESI-IT-TOF, lower) with marked TIC and XIC of oxidized **1** (disulfide-linked homodimer – 603.2844), sodium adduct of **1** $[M+Na]^+$ (626.2746) and its disulfide heterodimer with MESNa (744.2506). No hydrolyzed thioester was observed (**1b**); gradient: 5-65% B in 15', LC-ESI-IT-TOF instrument.

6.3. [Cys^5]-axinellin A ***2***

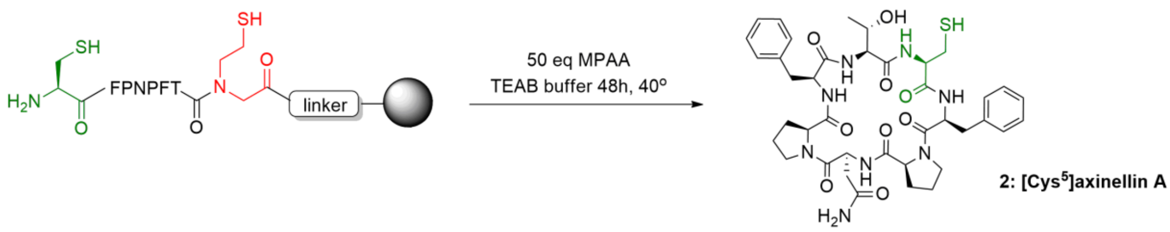

2: [Cys^5]axinellin A

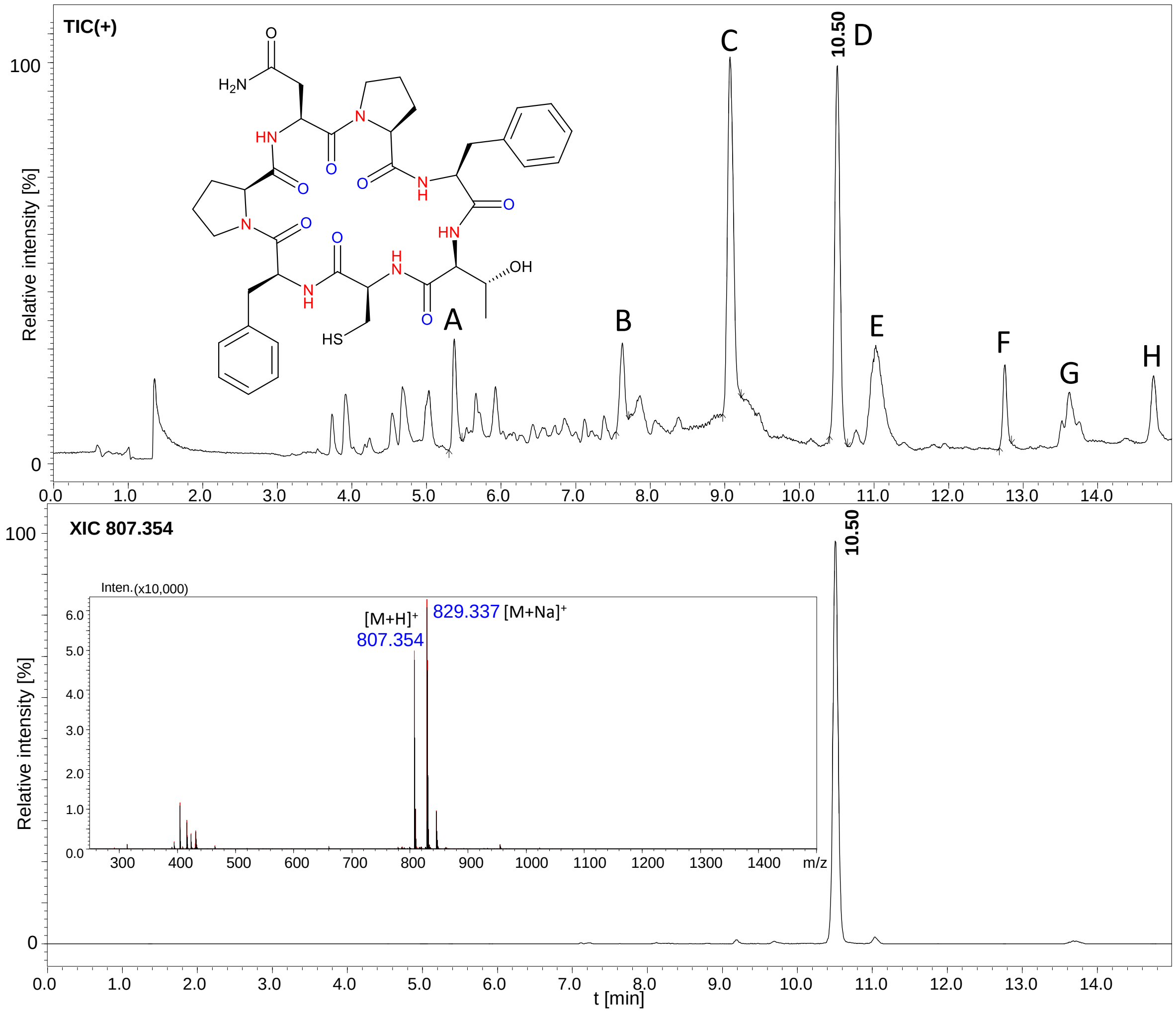

Name	rt (min)	m/z Found	m/z Calcd	Charge state	identification
A	5.36	393.211	-	1+	not identified (n.i.)
B	7.61	825.364	-	1+	not identified (n.i.)
C	9.01	357.024	-	1+	not identified (n.i.)
		402.043	-	1+	not identified (n.i.)
D	10.50	807.354	807.349	1+	[Cys^5]axinellin A
		829.337	829.332	1+	[Cys^5]axinellin A sodium adduct
E	10.99	924.379	-	1+	not identified (n.i.)
F	12.74	352.069	-	1+	not identified (n.i.)
		357.024	-	1+	not identified (n.i.)
G	13.62	327.010	-	1+	instrument impurity
I	14.73	354.2884	-	1+	not identified (n.i.)

Fig. S 26 LC-MS chromatogram of crude [Cys^5]axinellin A **2** obtained after liberation from solid support via tandem acyl shift and the subsequent native chemical ligation: upper chromatogram – Total Ion Current (TIC); lower chromatogram – extracted ion chromatogram (XIC) for m/z 807.354; gradient: 5-65% B in 15’, LC-ESI-IT-TOF instrument.

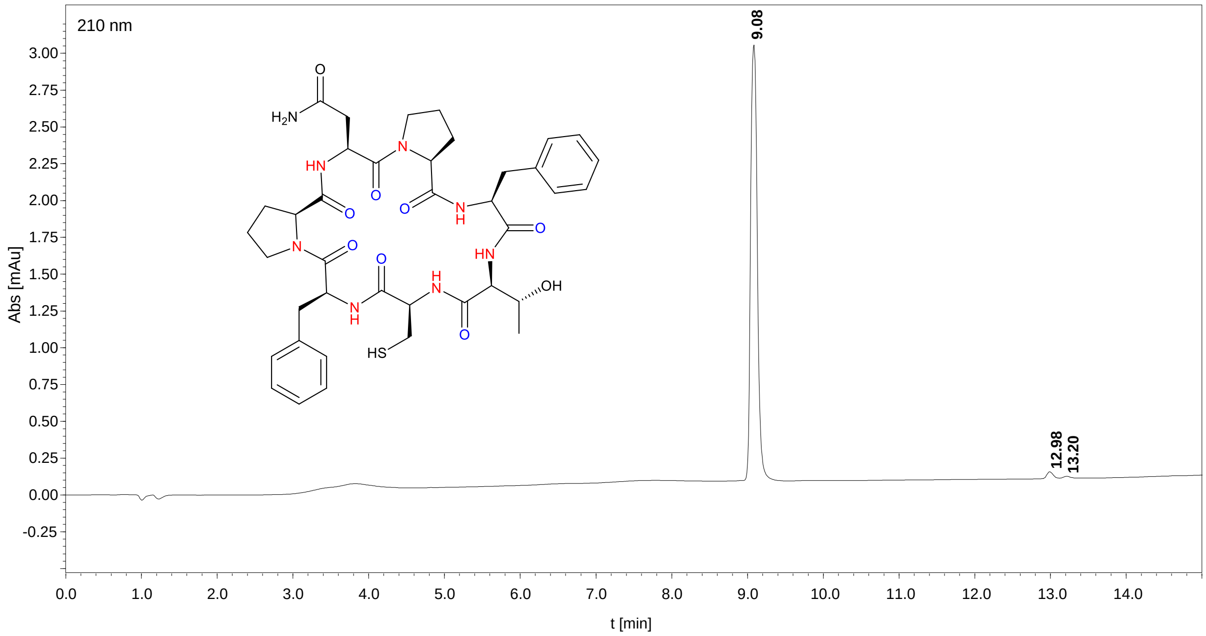

Fig. S 27 HPLC chromatogram of purified [Cys[5]]axinellin A **2** - detection at 210 nm (tiny signal at 12.98 min not identified) - gradient: 5-65% B in 15', Nexera XR LC-20AD

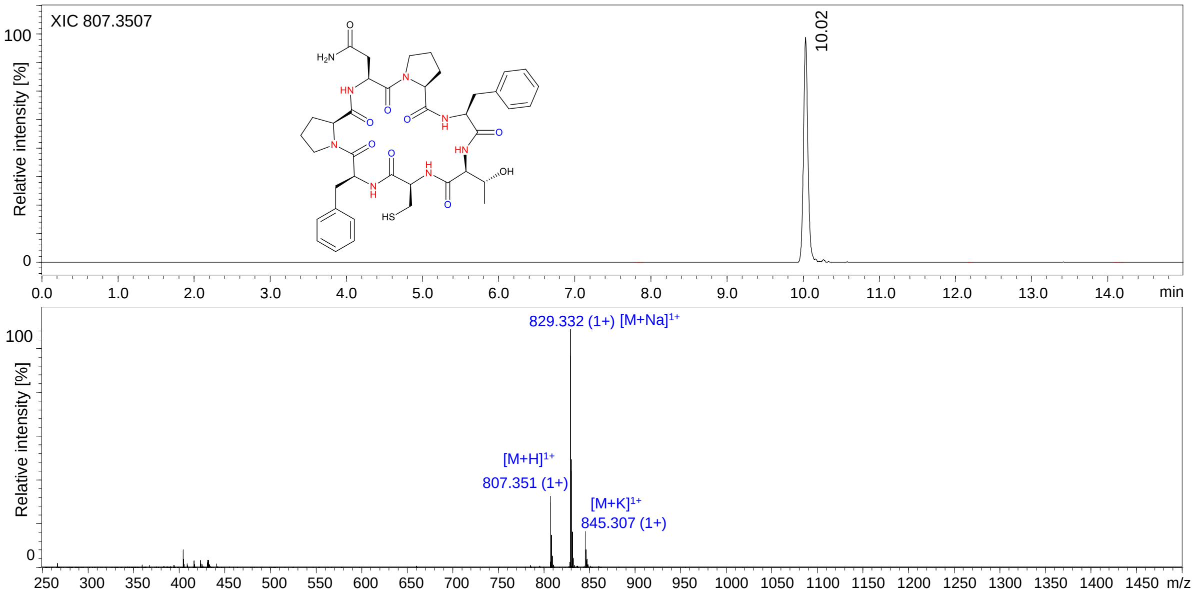

Fig. S 28 LC-MS chromatogram and ESI-MS spectrum acquired for purified [Cys[5]]axinellin A; LC-ESI-IT-TOF instrument

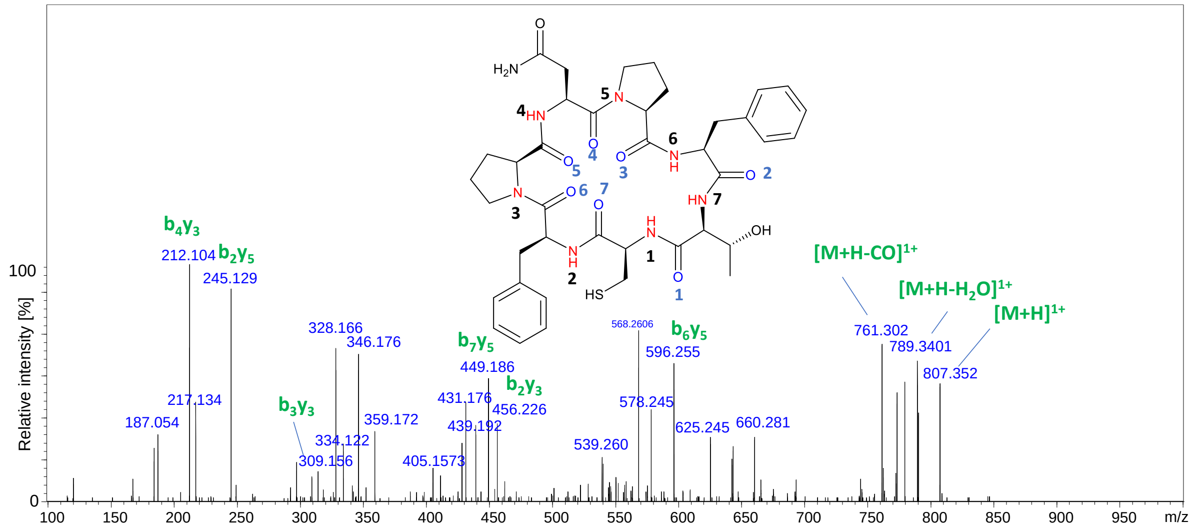

Fig. S 29 ESI-MS/MS fragmentation spectrum acquired for [Cys[5]]axinellin A **2** (*m/z* 807.351) – CE 25eV; ESI-FT-ICR instrument with CID fragmentation.

6.3.1. *[D-Thr[1],Cys[5]]axinellin A **2a** for enantiomerization study*

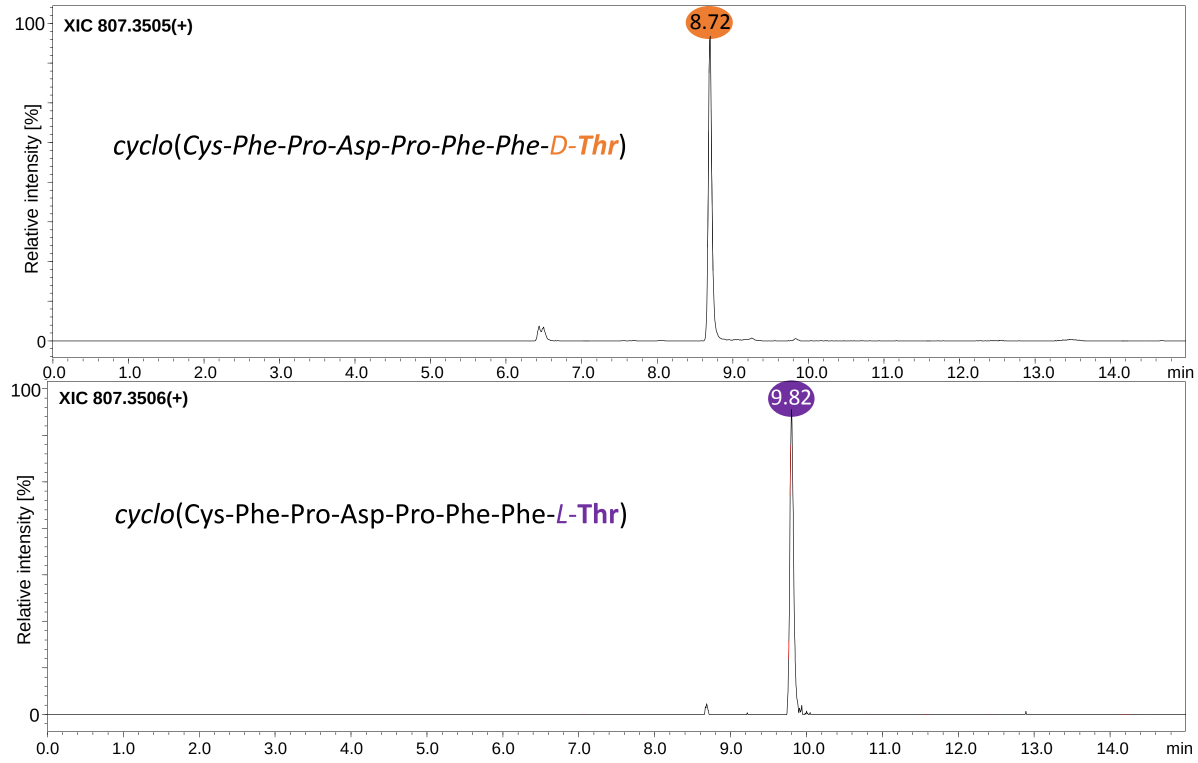

Fig. S 30 XIC comparison for [D-Thr[1],Cys[5]]axinellin A **2a** (upper) and [Cys[5]]axinellin A **2** (lower). Less than 1% enantiomerization is observed; gradient: 5-65% B in 15', LC-ESI-IT-TOF instrument.

*6.4. Cyclo CPKA **3***

6.4.1. Cyclization mediated by MESNa transthioesterification

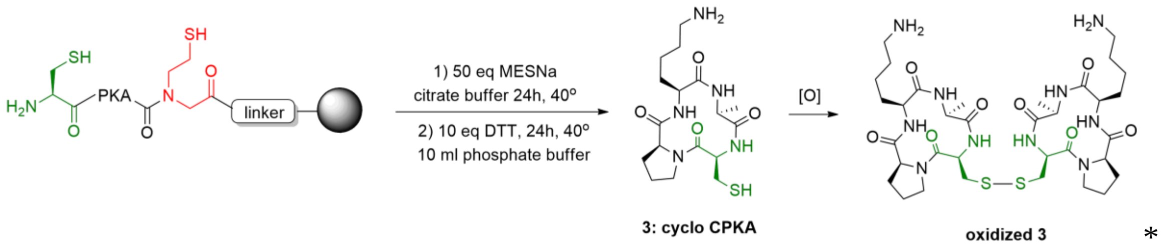

*Cyclic tetrapeptide 3 undergoes very fast oxidation forming disulfide-linked homodimer in HPLC conditions. LC-MS data are presented below for both forms.

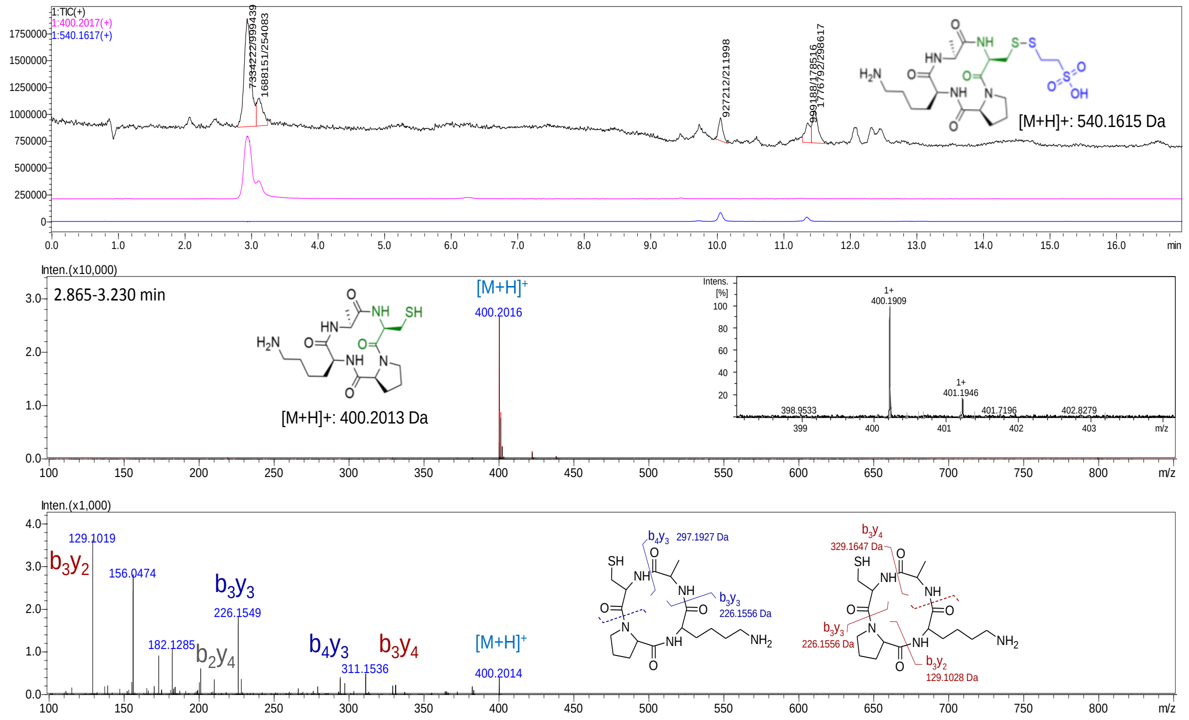

Fig. S 31 LC-MS/MS of purified **3**: upper part – MS chromatogram with TIC and XIC of 3 (400.2017) and its disulfide heterodimer with MESNa (shown on the scheme: *m/z* 540.1617); middle part – MS spectrum at 2.865-3.230 min with the zoomed-in isotopic pattern; lower part – MS/MS of parent ion: 400.2017, CE = 30 ± 5eV with the marked daughter ions; instrument LC-ESI-qTOF-MS, CID collision, gradient: 1% in 5', 1-10% in 5', 10% in 5', 10-100% in 3' B/A, oven 50°C.

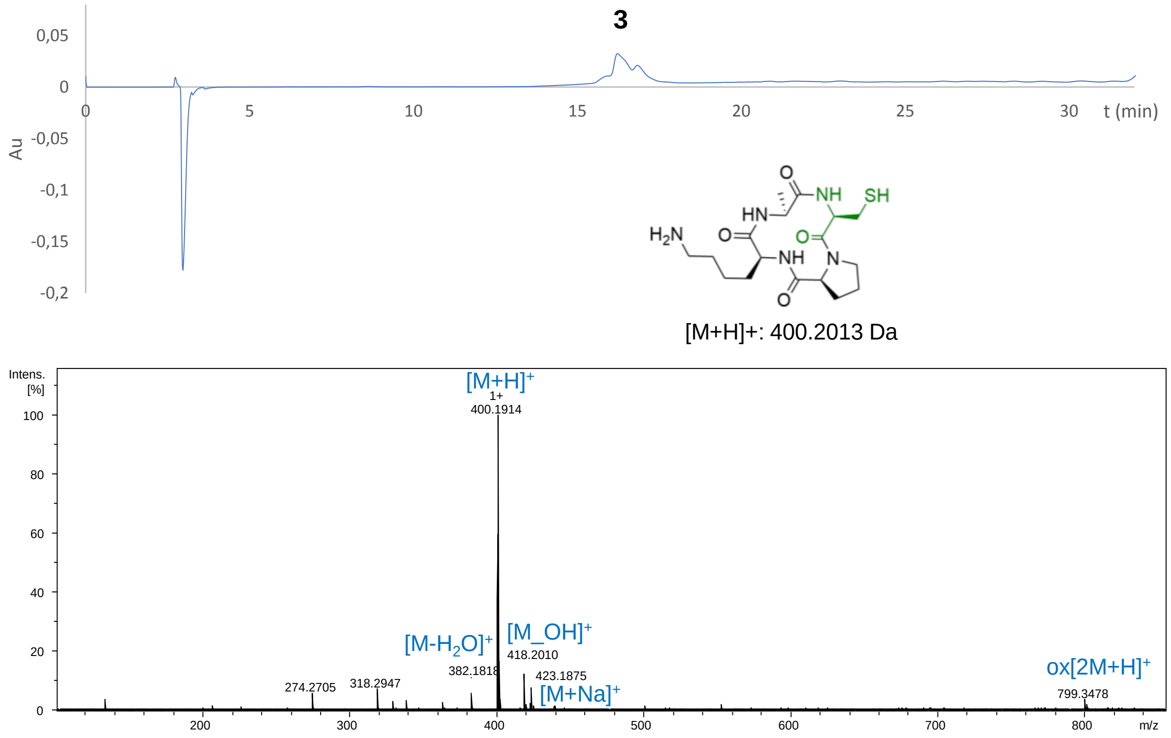

Fig. S 32 HPLC chromatogram (Thermo instrument, UV detection 210 nm, gradient: 1% in 8’, 1-10% in 10’, 10% in 10’, 10-100% in 6’) and MS spectrum (ESI-FT-ICR instrument) of purified **3**.

Analytical data for oxidized 3 – disulfide-linked heterodimer:

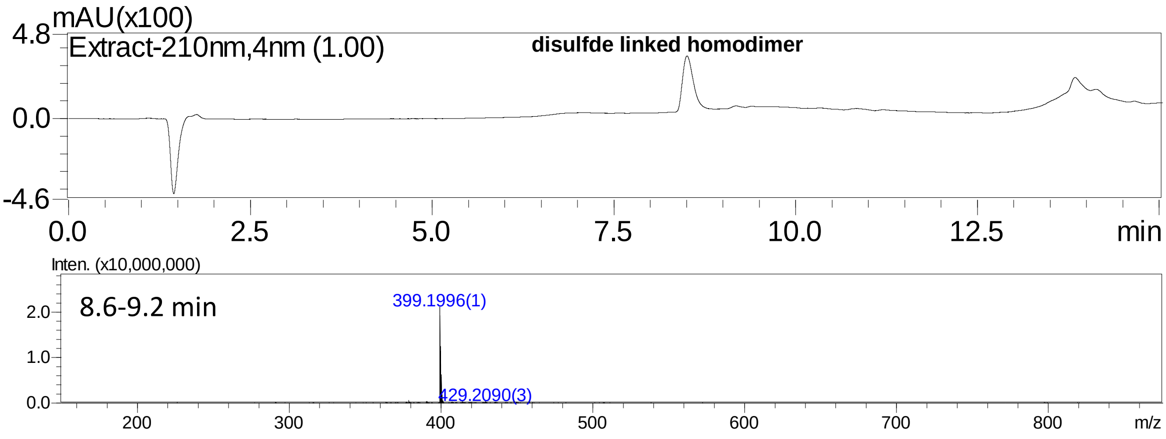

Fig. S 33 LC-UV-MS of purified **oxidized 3**: UV detection 210 nm, MS spectrum at 8.6-9.2 min, instrument ESI-LC-IT-TOF; gradient 1% in 2’, 1-10% in 5’, 10% in 3’, 10-100% in 8’ B/A, 50°C.

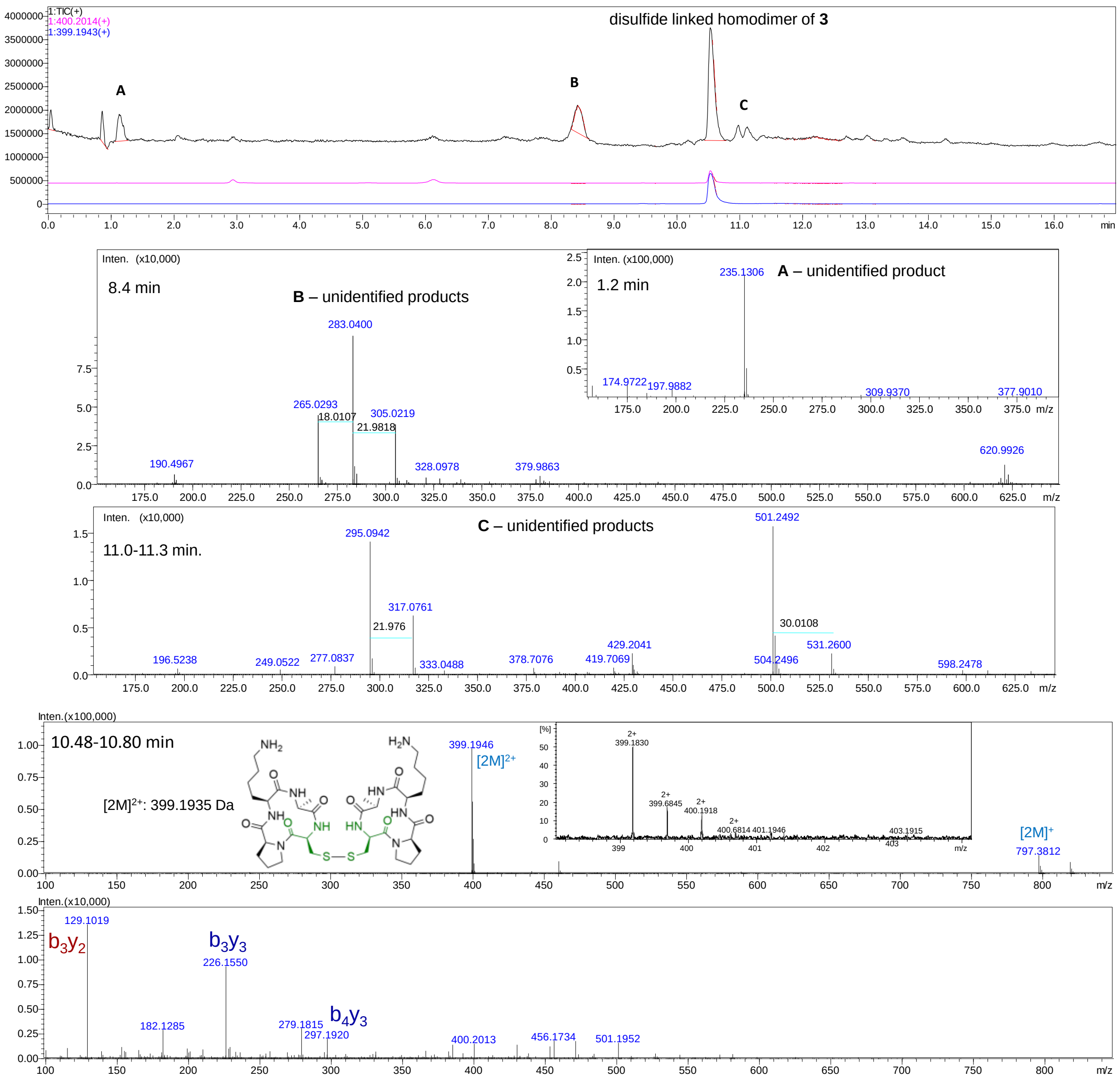

Fig. S 34 LC-MS/MS of purified **oxidized 3** (disulfide-linked homodimer): upper part – MS chromatogram with TIC and XIC of **oxidized 3** (399.1943); middle part – MS spectra of unidentified signals A, B, and C; lower part: MS spectrum at 10.48-10.80 min. with the zoomed-in isotopic pattern and MS/MS of parent ion: 399.1432, CE = 30 ± 5eV with the marked daughter ions; instrument LC-ESI-qTOF-MS, gradient: 1% in 5', 1-10% in 5', 10% in 5', 10-100% in 3' B/A, oven 50°C.

The remained resin, after cyclization reaction, was dried and treated with BrCN solution in TFA for 24h at rt, evaporated under the stream of nitrogen, and lyophilized.

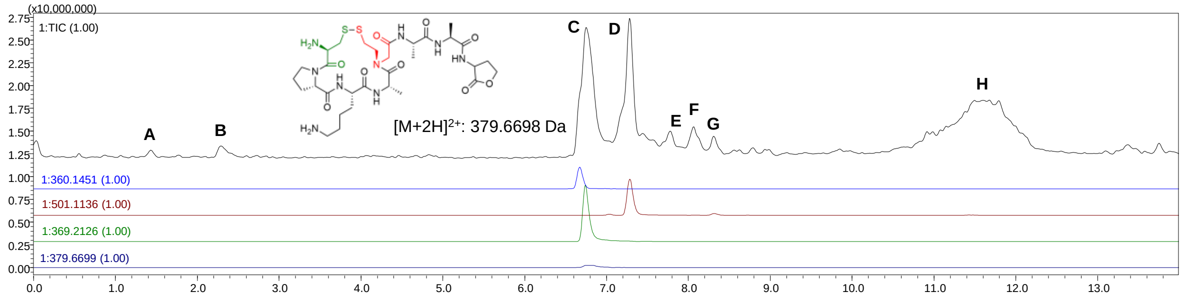

Name	rt (min)	m/z Found	m/z Calcd	charge state	identification
A	1.5	266.1108	-	1+	not identified (n. i.)
B	2.3	351.1628	-	1+	n. i
C	6.7	360.1451	360.1462	2+	disulfide linked homodimer [*N*-2-[thioethyl]glycine-AA-HL]$_2$
	6.8	369.2128	369.2132	1+	side-product of precursor synthesis: piperidine substitution with chloroacetic acid, $C_{17}H_{28}N_4O_5$ 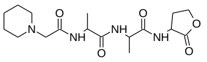
	6.8	379.6696	379.6698	2+	oxidised H-CPKA-*N*-2-[thioethyl]glycine-AA-HL with internal disulfide bond
D	7.3	501.1135	501.1142	1+	dilusfide linked heterodimer of *N*-2-[thioethyl]glycine-AA-HL with MESNa
E	7.8	386.0320	-	1+	n. i
F	8.1	520.6357	-	2+	n. i
G	8.4	500.1049	-	1+	n. i
H	11.0-12.0	-	-	5+, 4+	polymers from 441.2765 to 720.9377 with the charge 5+ and 8.807 Da mass difference and charge 4+ and 11.004 Da mass difference, respectively

Fig. S 35 LC-MS of the resin treated with BrCN, after the cyclization of peptide precursor **3**: TIC and XIC of: 360.1451 – [*N*-2-[thioethyl]glycine-AA-HL]$_2$ oxidized linker; 501.1136 – MESNa-*N*-2-[thioethyl]glycine-AA-HL (S-S bridged), 369.2128 – linker with piperidine substituted chloroacetic acid (side product from piperidine impurity); 379.6699 – unreacted peptide precursor CPKA-*N*-2-[thioethyl]glycine-AA-HL oxidized linker (with internal disulfide bridge), 5-65% B/A in 15’, rt, LC-ESI-IT-TOF instrument.

6.4.2. *Cyclization without any additional thiol*

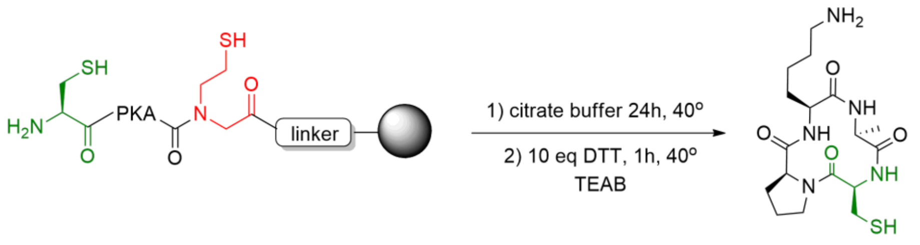

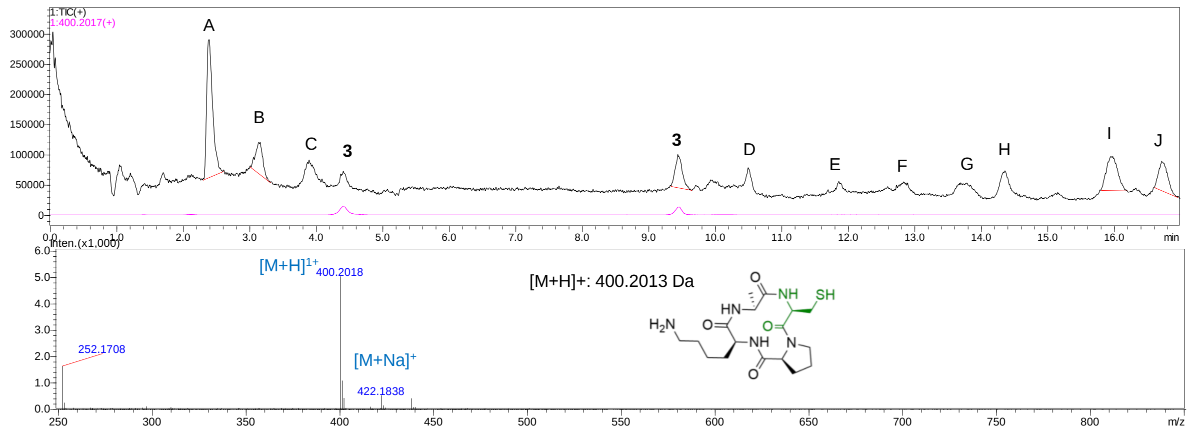

Name	rt (min)	m/z Found	m/z Calcd	charge state	identification
A	2.4	409.0198	-	1+	not identified (n. i.)
B	3.1	267.0630	-	1+	n. i.
C	3.9	252.1709	-	1+	n. i.
3	4.4	400.2018	400.2013	1+	**3** - cycloCPKA
D	10.5	418.2122	418.2119	1+	H-CPKA-OH (hydrolysed thioester)
E	11.9	283.0401<br>560.2189	-<br>-	1+<br>1+	n. i.<br>n. i.
F	12.9	305.1573<br>328.2332	-<br>-	1+<br>1+	n. i.<br>n. i.
G	13.8	333.1159	-	1+	n. i.
H	14.3	372.2595	-	1+	n. i.
I	15.9	377.1420	-	1+	n. i.
J	16.8	393.2098	-	1+	n. i.

Fig. S 36 LC-MS (LC-MS 9030, 1% in 5’, 1-10% in 5’, 10% in 5’, 10-100% in 3’, oven 60 C), MS spectrum of **3,** and signal identification of the crude product after the incubation of CPKA-*N*-2-[thioethyl]glycine-AAM-TentaGel NH2 in citrate buffer.

6.4.3. *Bicyclo CPKACPKA **3a***

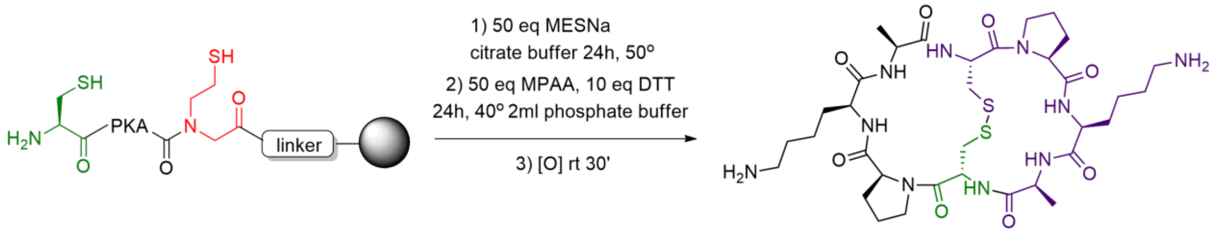

3a: bicyclo CPKACPKA

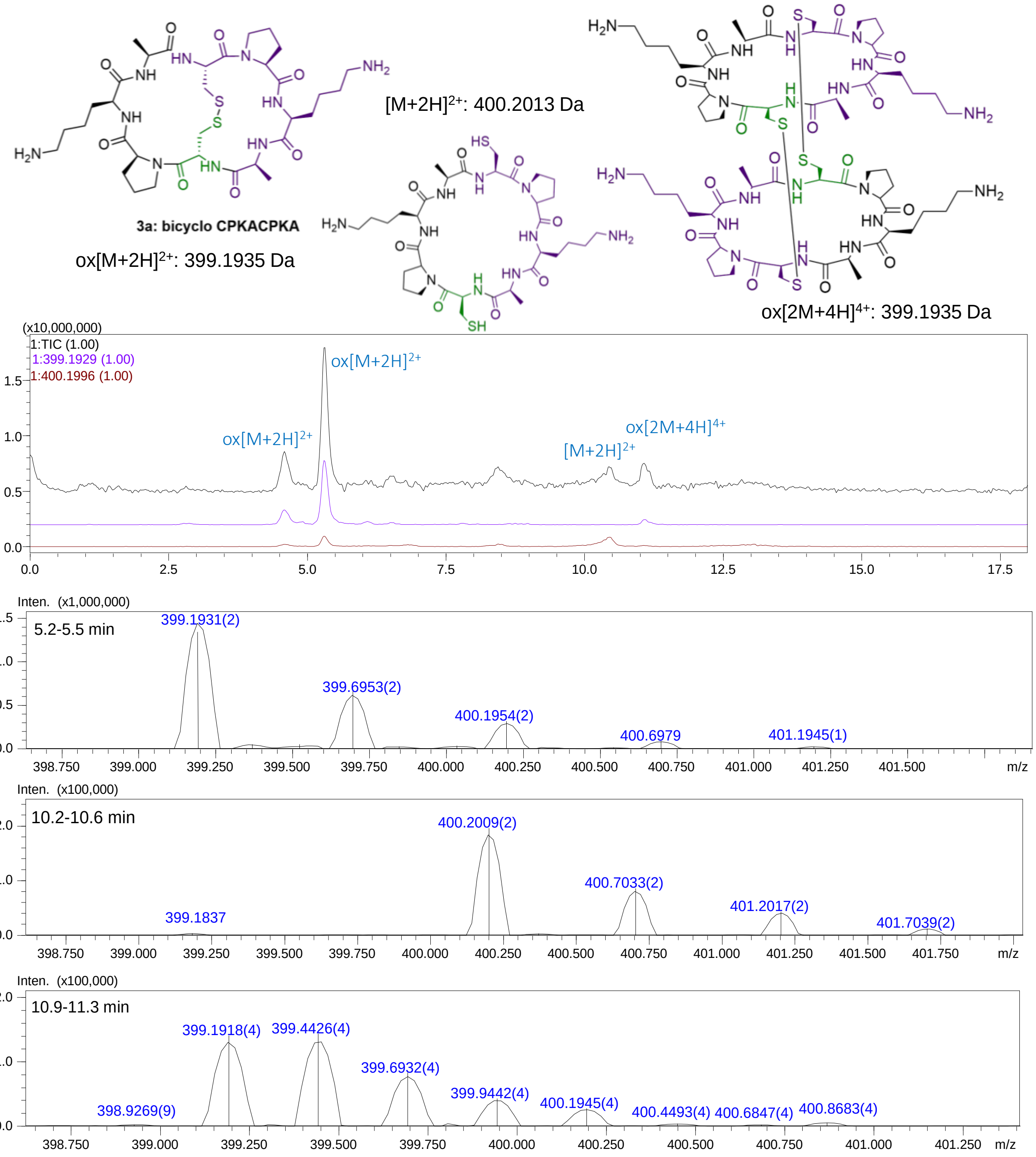

Fig. S 37 LC-MS (IT-TOF, 1% in 5', 5-10% in 10', 10% in 5', 10-100% in 3') of purified **3a** after air oxidation with the zoomed in isotopic patterns of the identified peptides presented above.

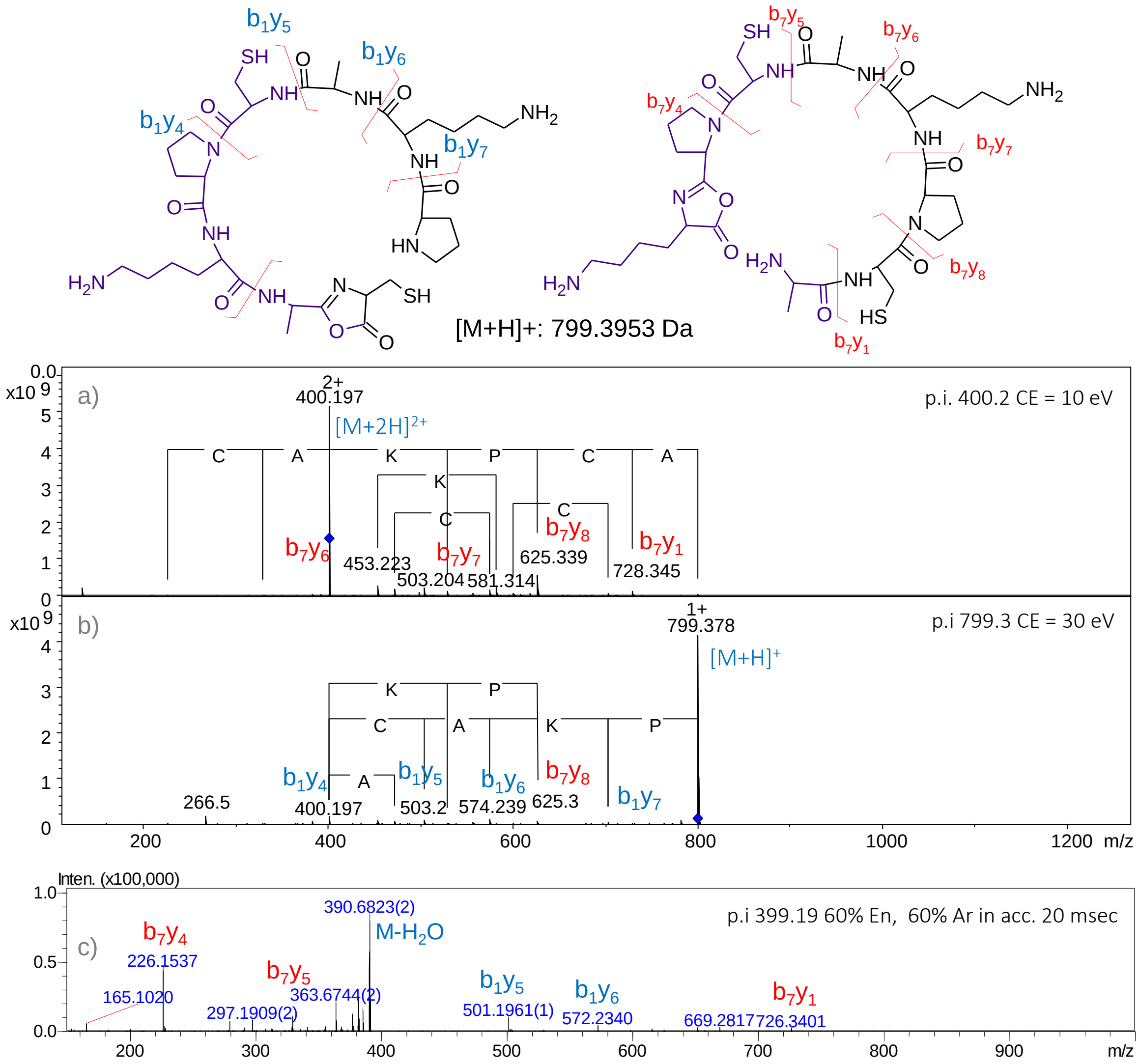

Fig. S 38 Fragmentation spectra of **3a** c) (p.i. 399.19 IT-TOF) and its reduced form with the parent ion corresponding to $[M+2H]^{2+}$ a) and $[M+H]^{+}$ b). Two fragmentation series are observed and their precursors are presented on the structures.

6.5. SFTI-1 ***4***

6.5.1. On-resin cyclization

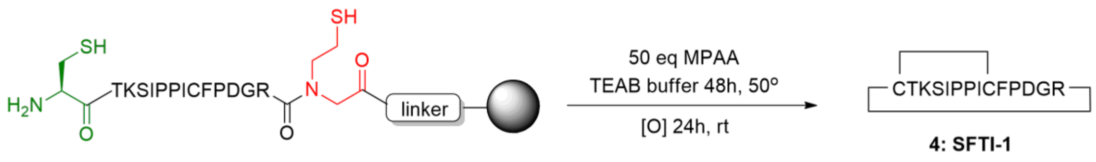

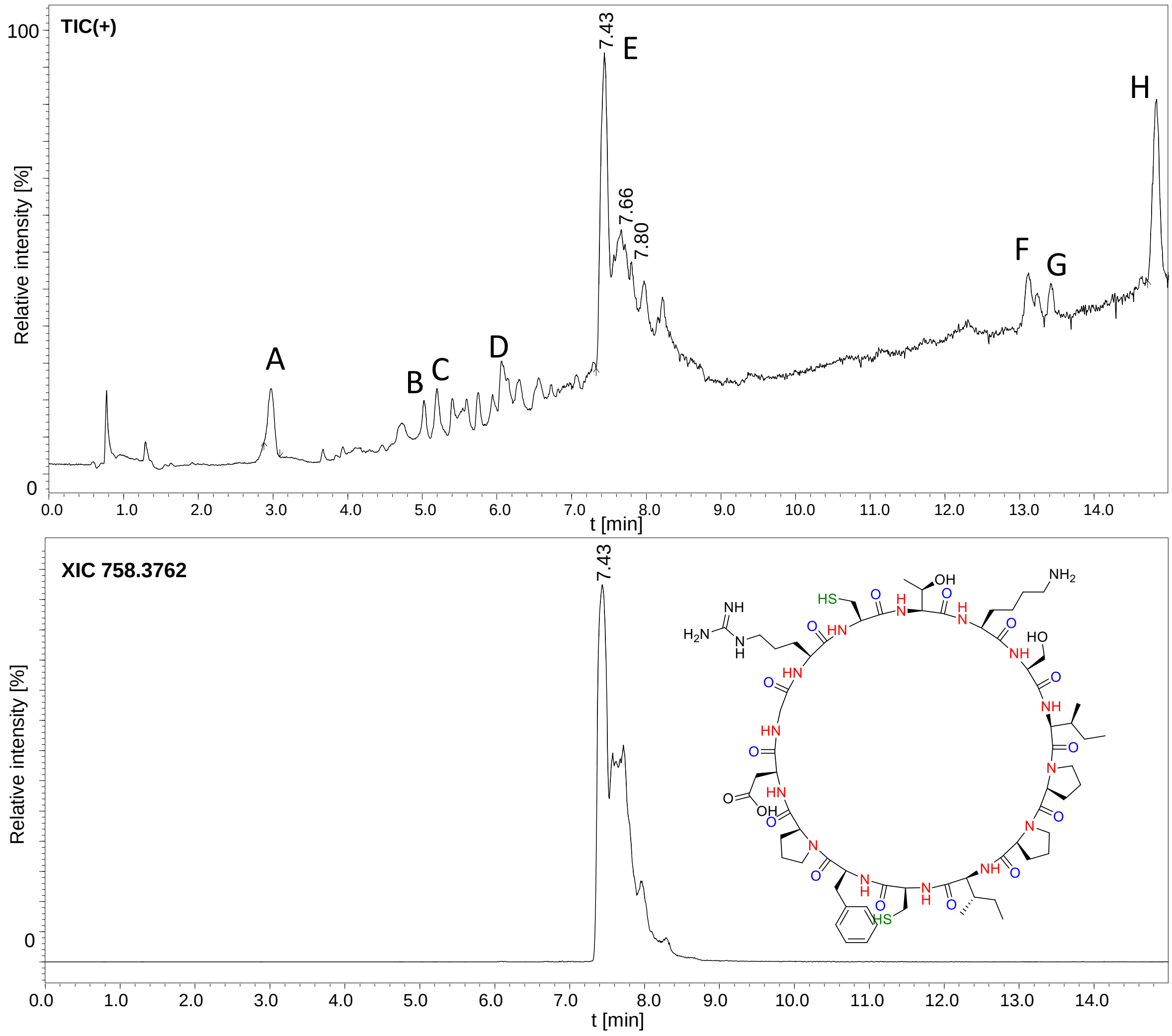

Name	rt (min)	m/z Found	m/z Calcd	Charge state	*identification*
A	2.97	362.9527	-	1+	not identified (n.i.)
B	5.03	393.2118	-	1+	not identified (n.i.)
C	5.21	377.1441	-	1+	not identified (n.i.)
D	6.07	597.9660	-	3+	not identified (n.i.)
E	7.36-8.19	758.3732	758.3762	2+	SFTI-1 (reduced)
F	13.08	327.0099	-	1+	instrument impurity
G	13.40	423.1698	-	1+	acetonitryle impurity
I	14.82	354.2884	-	1+	not identified (n.i.)

Fig. S 39 LC-MS chromatogram of crude and non-oxidized SFTI-1 obtained after liberation from solid support via tandem acyl shift and the subsequent native chemical ligation: upper chromatogram – Total Ion Current (TIC); lower chromatogram – extracted ion chromatogram for *m/z* = 758.3762.

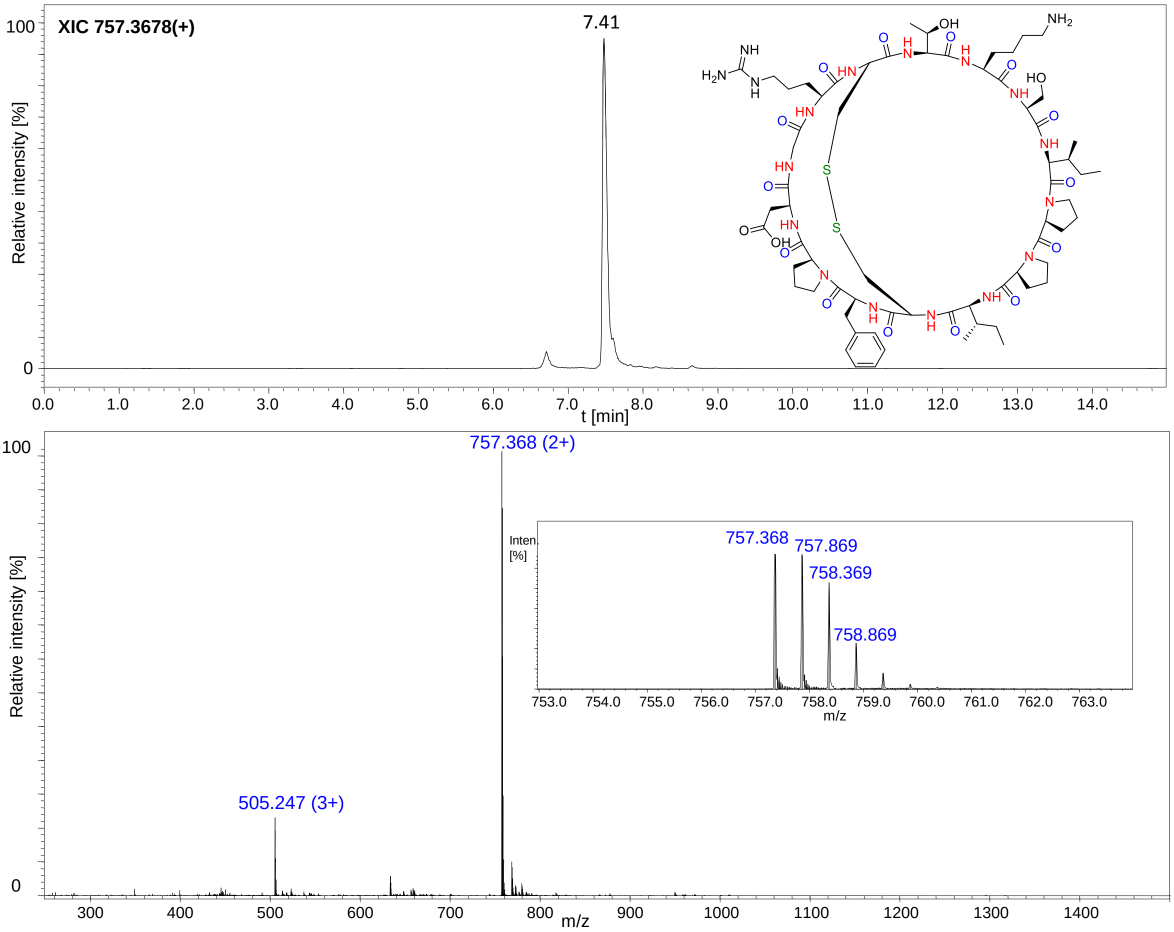

Fig. S 40 LC-MS chromatogram (XIC – 757.368 *m/z*) and ESI-MS spectrum acquired for purified SFTI-1 analogue.

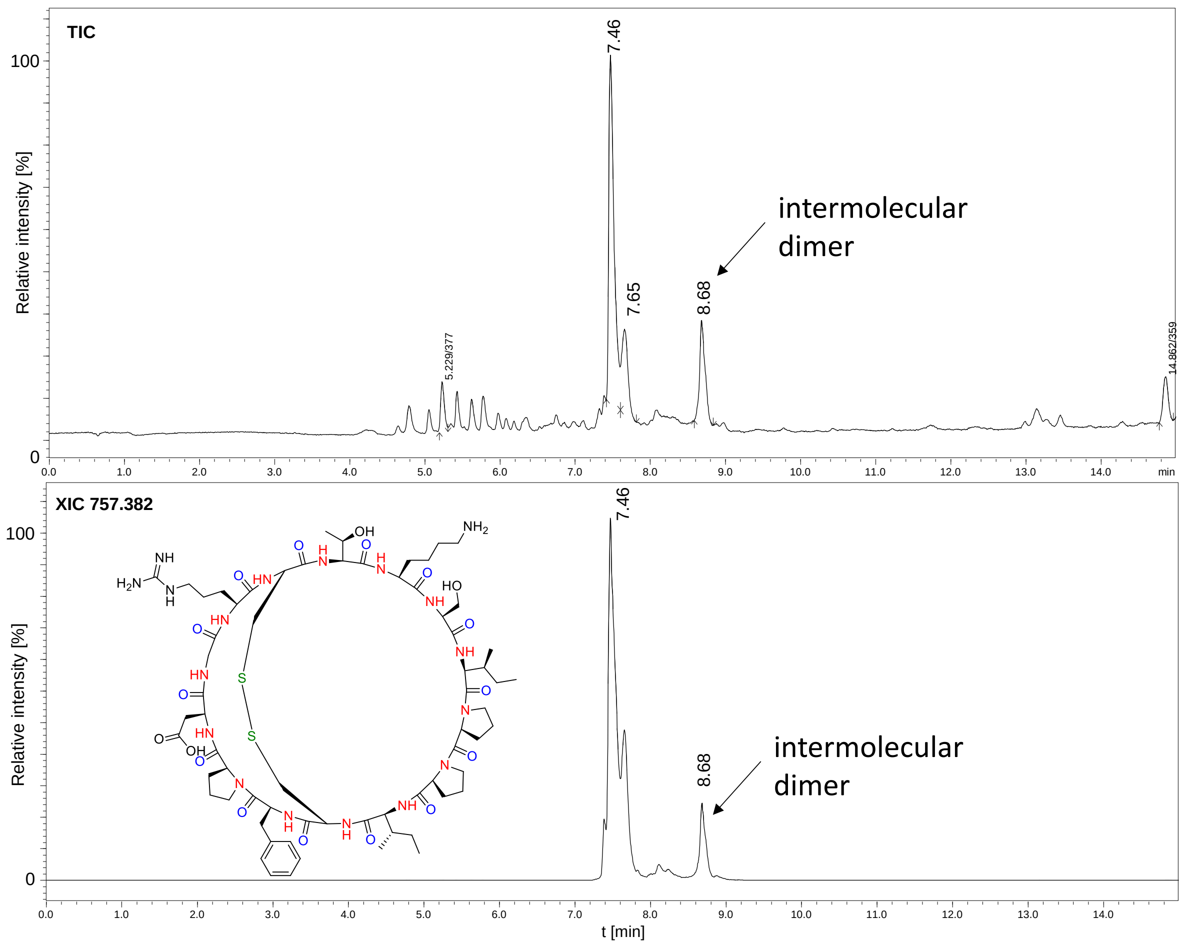

Fig. S 41 LC-MS chromatogram TIC and XIC (757.368 *m/z*) obtained for purified and oxidized SFTI-1.

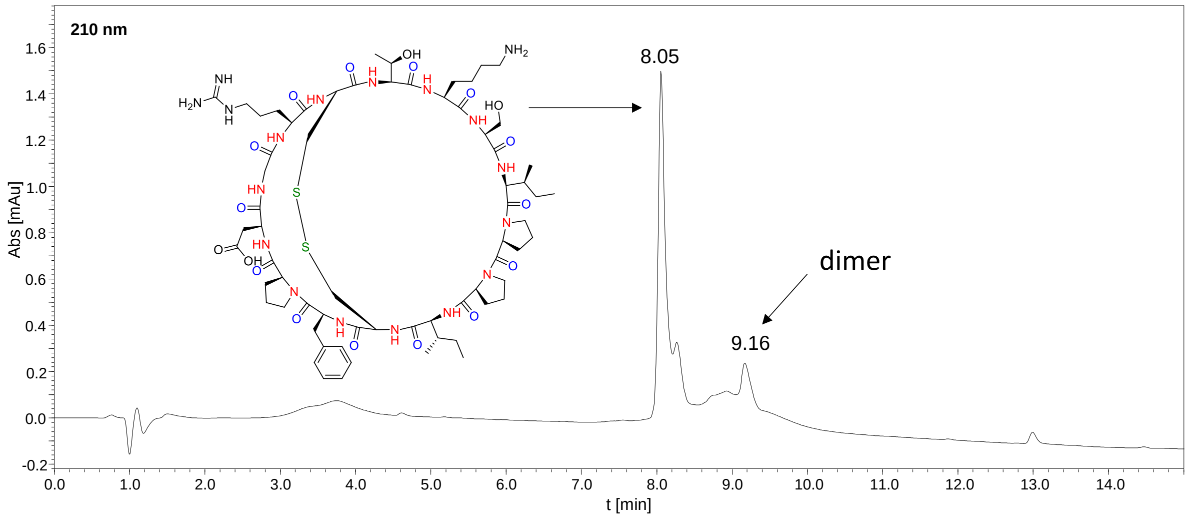

Fig. S 42 HPLC chromatogram of purified and oxidized SFTI-1 (detection at 210 nm).

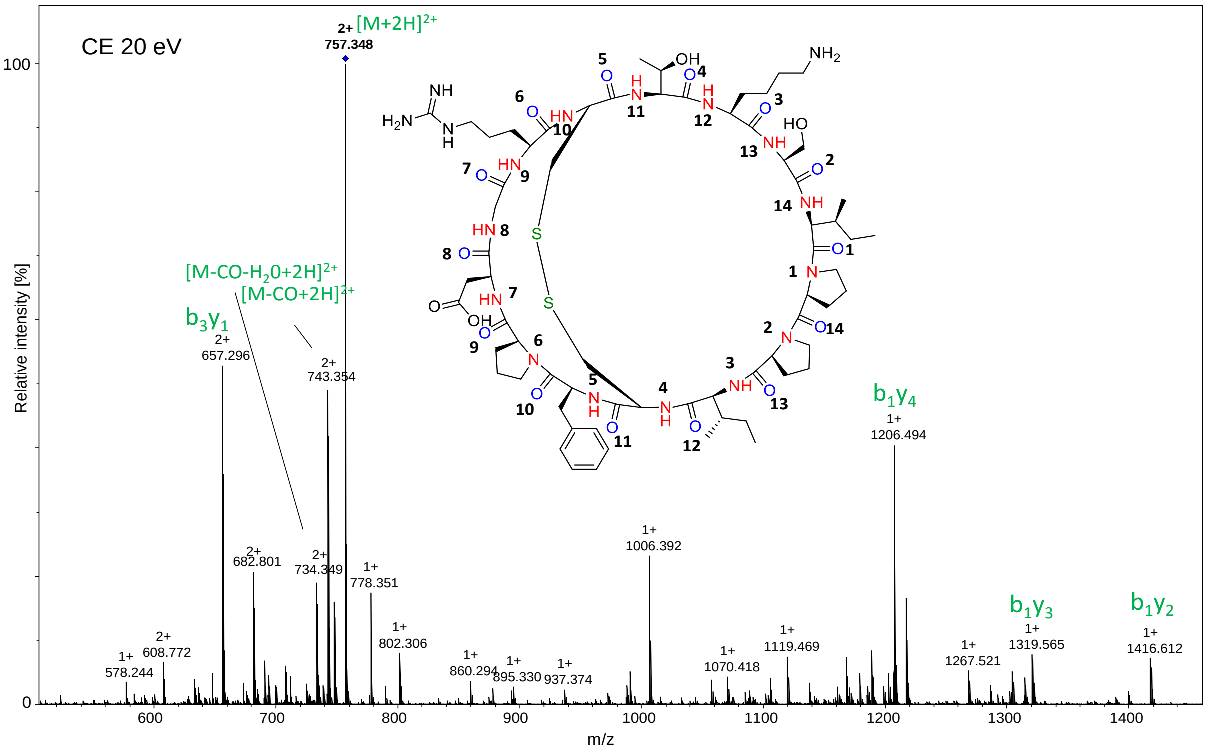

Fig. S 43 ESI-MS/MS fragmentation spectrum acquired for SFTI-1 (757.348 *m/z*) – CE 20eV.

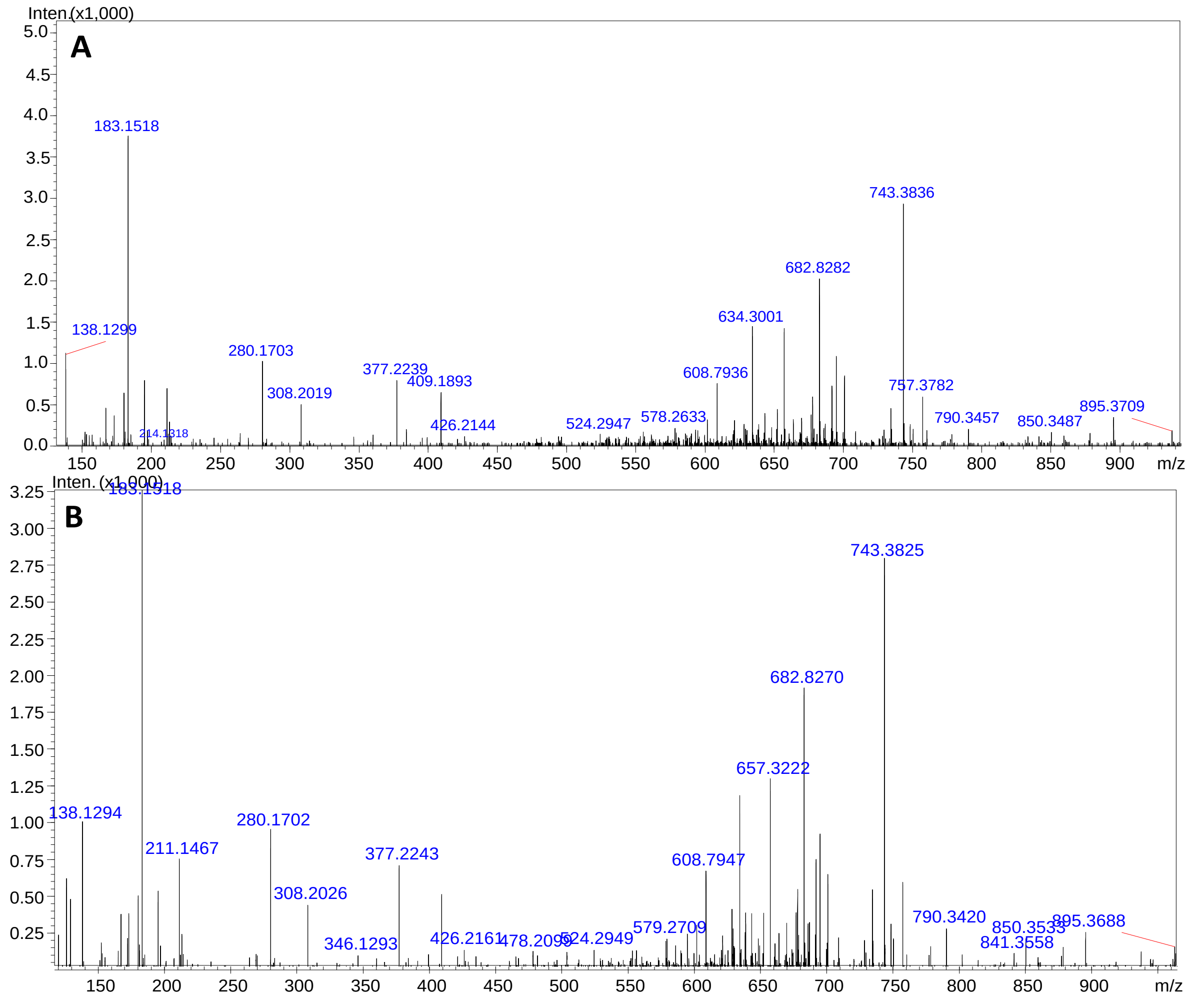

Fig. S 44 Comparison of ESI-MS/MS spectra acquired for the reference sample (A) and the synthetic SFTI-1 (B) on the LCMS9030 instrument.

6.5.2. Cyclization in solution

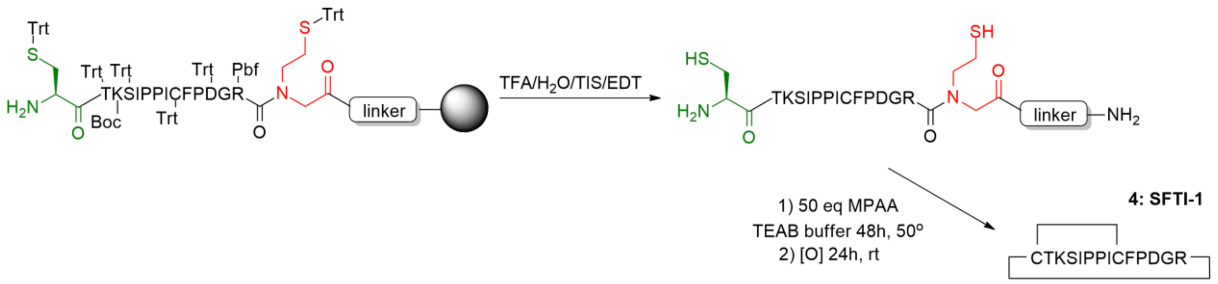

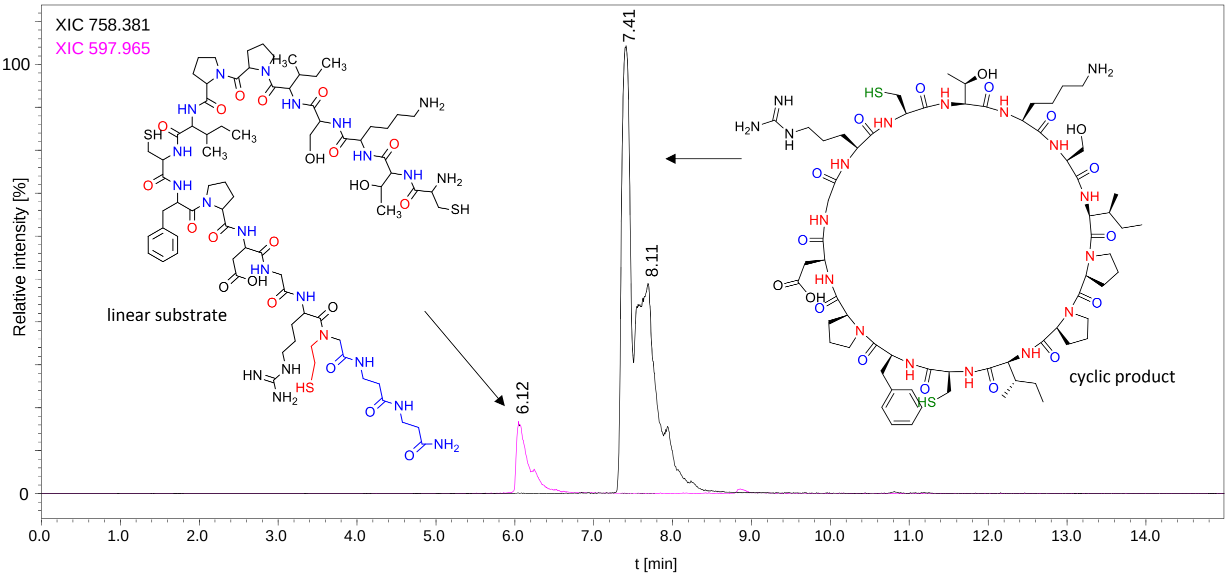

Fig. S 45 LC-MS chromatogram of crude and non-oxidized SFTI-1 obtained in solution via tandem acyl shift and the subsequent native chemical ligation: extracted ion chromatogram XIC for 758.382 (2+) *m/z* (cyclic product) and 597.965 (2+) *m/z* (linear precursor).

6.6. *Θ-defensin RTD-1* ***5***

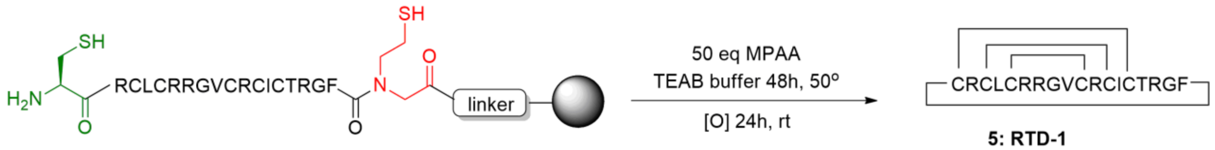

5: RTD-1

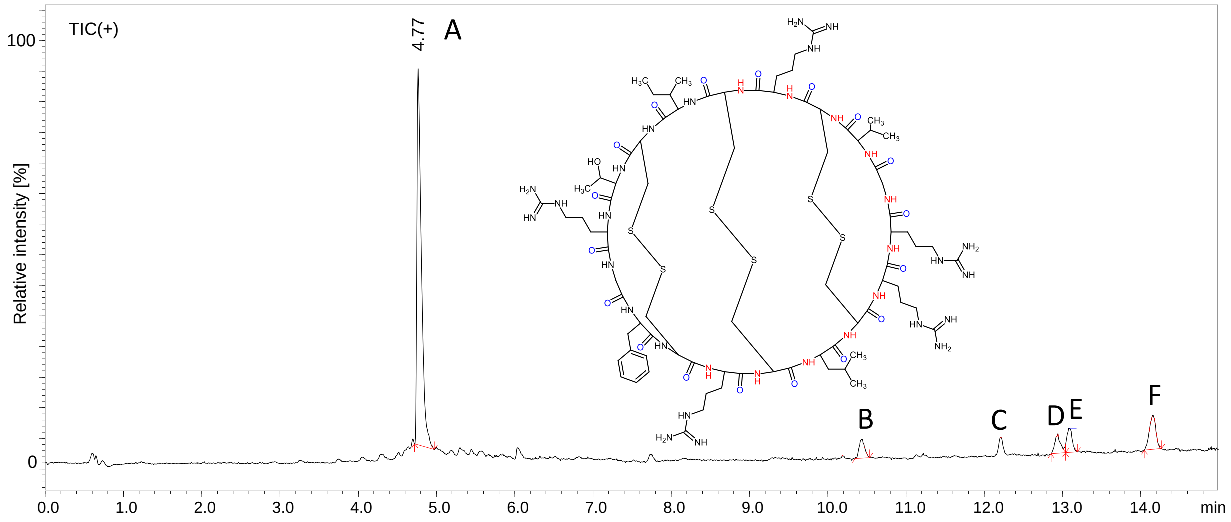

Name	rt (min)	m/z Found	m/z Calcd	Charge state	identification
A	4.77	520.9862	520.9864	4+	Ө-defensin
		416.9909	416.9907	5+	Ө-defensin
B	10.43	273.1284	-	1+	not identified (n.i.)
C	12.18	352.0676	-	1+	not identified (n.i.)
			-		
D	12.93	327.0085		1+	instrument impurity
E	13.07	423.1680	-	1+	acetonitryle impurity
F	12.74	359.2411	-	1+	not identified (n.i.)

Fig. S 46 LC-MS chromatogram (TIC) obtained for purified and oxidized Θ-defensin RTD-1.

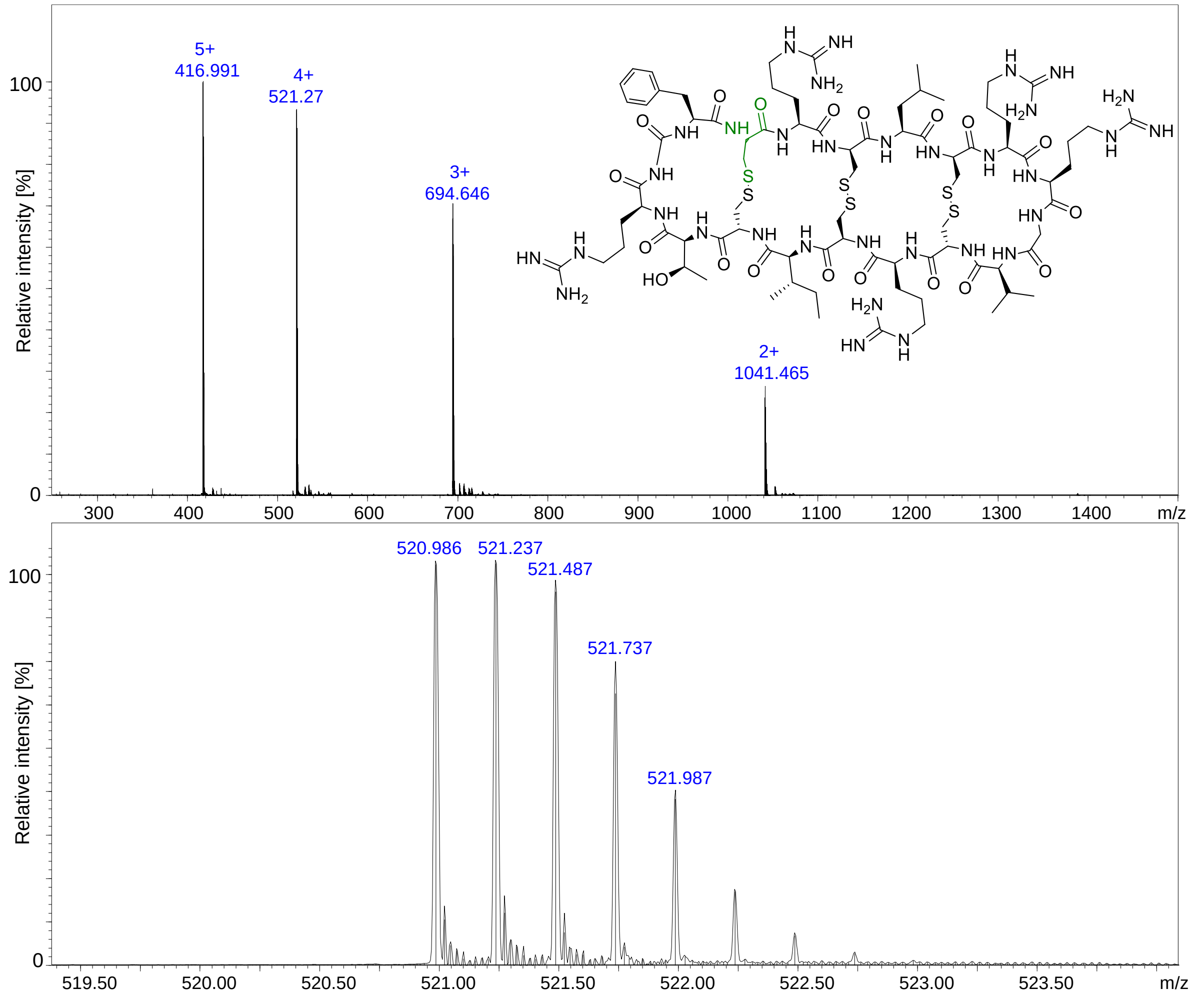

Fig. S 47 ESI-MS spectrum acquired for purified Θ-defensin RTD-1.(upper) and spectrum with the expanded 520.896 (4+) *m/z* signal (lower).

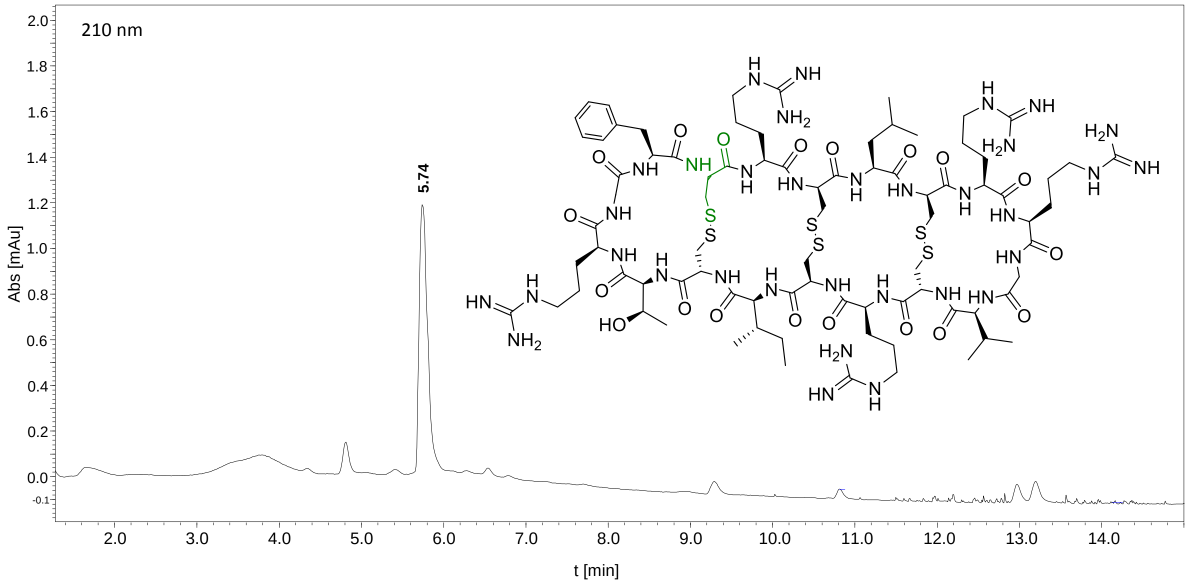

Fig. S 48 HPLC chromatogram of purified Θ-defensin RTD-1 (detection at 210 nm); Nexera XR LC-20AD
